# Multi-Omics Insights into Disulfidptosis-Related Genes Reveal RPN1 as a Therapeutic Target for Liver Cancer

**DOI:** 10.3390/biom14060677

**Published:** 2024-06-10

**Authors:** Yan He, Yue Hu, Yunsheng Cheng, Xutong Li, Chuanhong Chen, Shijie Zhang, Huihu He, Feng Cao

**Affiliations:** 1 Vascular Surgery, Department of General Surgery, The First Hospital of Anhui Medical University, Hefei 230001, China; 2 Pathology Department, Hefei Cancer Hospital, Chinese Academy of Sciences (CAS), Hefei 230000, China; huyue@cmpt.ac.cn; 3 Department of General Surgery, The Second Affiliated Hospital of Anhui Medical University, Hefei 230000, China; 4 Department of Infectious Diseases, The First Hospital of Anhui Medical University, Hefei 230001, China; 5 Department of General Surgery, The Fuyang Hospital of Anhui Medical University, Fuyang 236000, China; zsj161592@163.com; 6 Medical Faculty, University Hospital RWTH Aachen, 52074 Aachen, Germany

**Keywords:** disulfidptosis, pan-cancer, programmed cell death, liver cancer, ribophorin 1

## Abstract

Disulfidptosis, a newly identified mode of programmed cell death, is yet to be comprehensively elucidated with respect to its multi-omics characteristics in tumors, specific pathogenic mechanisms, and antitumor functions in liver cancer. This study included 10,327 tumor and normal tissue samples from 33 cancer types. In-depth analyses using various bioinformatics tools revealed widespread dysregulation of disulfidptosis-related genes (DRGs) in pan-cancer and significant associations with prognosis, genetic variations, tumor stemness, methylation levels, and drug sensitivity. Univariate and multivariate Cox regression and LASSO regression were used to screen and construct prognosis-related hub DRGs and predictive models in the context of liver cancer. Subsequently, single cell analysis was conducted to investigate the subcellular localization of *RPN1*, a hub DRG, in various solid tumors. Western blotting was performed to validate the expression of *RPN1* at both cellular and tissue levels. Additionally, functional experiments, including CCK8, EdU, clone, and transwell assays, indicated that *RPN1* knockdown promoted the proliferative and invasive capacities of liver cancer cells. Therefore, this study elucidated the multi-omics characteristics of DRGs in pan-cancer and established a prognostic model for liver cancer. Additionally, this study revealed the molecular functions of *RPN1* in liver cancer, suggesting its potential as a therapeutic target for this disease.

## 1. Introduction

Cancer is a major global public health concern. The latest global cancer statistics indicate that there were approximately 19.29 million new cases and 10 million deaths due to cancer in 2020, with a projected 47% increase in newly diagnosed cases to 28.4 million by 2040 [1]. Despite advancements in cancer diagnosis and treatment, liver cancer continues to have poor prognosis. The 5-year survival rate for liver cancer is below 20%, which is one of the lowest among all tumor types [2,3]. Alpha-fetoprotein (AFP) is a widely used biomarker for early screening and prognostic evaluation of hepatocellular carcinoma (HCC). However, the diagnostic accuracy of this marker is limited owing to its low sensitivity and specificity. Moreover, the expression of this marker is influenced by various cytokines and is significantly elevated in other diseases including acute viral hepatitis [4,5]. The lack of specific biomarkers has resulted in the diagnosis of advanced-stage liver cancer in most patients. Furthermore, in recent years, the COVID-19 pandemic has increased competition for medical resources, resulting in a higher incidence of advanced cancer and mortality [6]. This has further exacerbated the diagnostic and therapeutic burden of liver cancer. Therefore, identification of novel biomarkers, in-depth understanding of the molecular mechanisms underlying cancer initiation and progression, and exploration of potential therapeutic targets are urgently required.

Increasing evidence suggests that programmed cell death (PCD) mechanisms play a crucial role in the development of malignant tumors [7,8,9]. Disulfidptosis is a recently discovered mode of PCD characterized by the abnormal accumulation of disulfides, specifically cystine, in cells with high expression of solute carrier family 7 member 11 (SLC7A11), particularly under conditions of glucose deprivation. This phenomenon induces disulfide stress, leading to an increase in the number of disulfide bonds in the actin cytoskeleton. Consequently, disruption of cytoskeletal structure leads to cell death [10]. However, recent studies have revealed that SLC7A11 is aberrantly expressed in various cancers and is a major determinant of cancer cell dependency on glucose and glutamine [11]. Glucose transporter inhibitors suppress tumor cell proliferation by triggering disulfidptosis [12]. Additionally, ribophorin 1 (RPN1), another gene associated with disulfide-mediated apoptosis, encodes a key subunit of the oligosaccharyltransferase (OST) complex, which is crucial for N-linked glycosylation. Studies suggest that aberrant protein glycosylation can mediate tumor progression by activating the endoplasmic reticulum stress (ERS) signaling pathways [13,14]. Ding et al. demonstrated that knockdown of RPN1 leads to ERS activation, thereby inhibiting proliferation and invasion while promoting apoptosis in breast cancer (BC) cells [15]. These studies have indicated the close association between disulfide metabolism and various biological processes in cancer cells, including invasion, metastasis, immune evasion, and drug resistance [16,17]. These findings highlight the significant potential of disulfidptosis in cancer therapy. Several studies have extensively investigated the transcriptomic characteristics and potential functions of disulfidptosis in bladder [18], esophageal [19], and lung [20] cancers. However, the extent to which it contributes to liver cancer development has not been sufficiently studied. Moreover, these investigations have been limited to specific tumor types, with constraints such as small sample sizes and high heterogeneity.

Therefore, this study aimed to analyze the multi-omics characteristics of disulfidptosis in pan-cancer by integrating multiple cancer types, thereby providing a more comprehensive and in-depth analysis. This study provides valuable insights by accurately identifying disulfidptosis-related hub genes and comprehensively analyzing and confirming the specific functions of these genes in liver cancer using transcriptomic data, single cell data, and experimental validation. Additionally, the findings of this study improve our understanding of the potential role of disulfidptosis-related genes (DRGs) in pan-cancer and provide novel strategies for the diagnosis and treatment of liver cancer.

## 2. Methods and Material

### 2.1. Data Sources

We obtained 39 DRGs from the previously published literature (10) (Table S1). Subsequently, we downloaded fragments per kilobase per million mapped fragments (FPKM) expression data and clinical data for 10,327 samples of 33 cancer types from TCGA database. The tumor types and sample numbers are presented in Table S2. Additionally, we acquired copy number variation (CNV), single nucleotide variation (SNV), and methylation data from TCGA database. Gene and drug sensitivity data were downloaded from the Genomics of Drug Sensitivity in Cancer (GDSC) and Cancer Therapeutic Response Portal (CTRP) databases. Finally, we obtained transcriptomic datasets for liver cancer (GSE14520) and single cell datasets for various tumors, including lung adenocarcinoma (GSE123902), liver cancer (GSE138709), colon cancer (GSE188711), breast cancer (GSE180286), clear cell renal cell carcinoma (GSE156632), esophageal adenocarcinoma (GSE222078), gastric cancer (GSE183904), and pancreatic cancer (GSE154778) from the GEO database.

### 2.2. Patient Population and Tissue Specimens

Five patients with liver cancer, who underwent surgical treatment at the Hefei Cancer Hospital of the Chinese Academy of Sciences, were randomly selected for this study. Following surgical resection of the tumor tissues, the excised tissues were rinsed with sterile saline under aseptic conditions. Sections of the tumor and adjacent normal tissues, approximately 1–2 cm in diameter, were then placed in separate Eppendorf tubes and immediately transferred to liquid nitrogen for cryopreservation. This study was conducted in compliance with the guidelines, regulations, and principles outlined in the Declaration of Helsinki.

### 2.3. Differential Analysis of DRG Expression and Prognosis

The expression levels of DRGs in 10,327 samples were analyzed using the “ggboxplot” package in R and were visualized as boxplots. Additionally, we conducted Spearman’s correlation analysis of DRGs at the pan-cancer level using the “corplot” package. Based on the expression levels of DRGs, we performed differential expression analysis between tumor and normal samples using the “limma” and “pheatmap” packages, with the parameters set as |Log2 FC| > 1.5 and false discovery rate (FDR) < 0.05. Next, we extracted the expression profiles of the DRGs that correlated with clinical survival data. The patients were subsequently categorized into high- and low-expression groups based on median gene expression levels. Subsequently, log-rank tests for survival time were conducted using the “survival” and “survminer” packages and plotted Kaplan–Meier (KM) survival curves. Furthermore, we conducted Cox regression analysis of DRGs in 33 cancer types using clinical data. Forest plots were generated using the “forestplot” package.

### 2.4. Analysis of Genetic Variation in DRGs

We analyzed CNV data for the 33 types of cancer using GISTIC2.0 software. Subsequently, we analyzed and graphically represented the distribution of CNV types in pan-cancer DRGs using the “maftools” and “ggplot2” packages. We integrated the CNV data with the mRNA data of DRGs and used the “corplot” package to analyze the correlation between the CNV percentages of DRGs and their corresponding mRNA expression levels. Based on the CNV data, we categorized the patients into two groups: CNV-positive and CNV-negative. We further compared survival rates between the two groups, including disease-free interval (DFI) and overall survival (OS). We used the “limma” and “pheatmap” packages to analyze and visualize the percentage heatmap of SNVs in the DRGs. Subsequently, we used the “maftools” package to investigate the mutation patterns of DRGs in 33 cancer types and generated waterfall plots. Finally, we performed a univariate Cox regression analysis to examine the survival differences between wild-type and mutant-type patients for DRGs.

### 2.5. Analysis of Tumor Stemness and Methylation Characteristics

Tumor stemness score is a method for evaluating the differentiation potential of tumor cells. This approach typically involves the analysis of RNA expression (RNAss) and DNA methylation (DNAss). In this study, we assessed the individual correlations between RNAss, DNAss, and DRG mRNA expression levels using Spearman’s analysis. In addition, we selected 14 tumor and > 10 normal tissue samples. We analyzed differences in methylation levels using paired t-tests. The observed differences were adjusted using the FDR for *p* values. Subsequently, Spearman’s correlation analysis was performed to investigate the correlation between the mRNA expression of DRGs and their methylation levels. We then integrated the clinical data and categorized patients into high- and low-methylation groups. Cox regression analysis was conducted to explore differences in DFI, disease-specific survival (DSS), OS, and progression-free survival (PFS) between the two groups. We evaluated the risk factors associated with methylation levels using *p*-values and hazard ratios.

### 2.6. Gene Set Variation and Pathway Enrichment Analyses

To analyze the pathway enrichment of DRGs in pan-cancer, we first used the “GSVA” package to convert individual DRGs in the samples into a gene set expression matrix and calculate gene set variation analysis (GSVA) scores. Next, we used the Wilcoxon test and analysis of variance (ANOVA) to analyze the variations in GSVA scores across different tumor subtypes. Additionally, we used the Mann–Kendall trend test to examine the GSVA score trends across various tumor stages. Subsequently, we categorized the patients into high- and low-GSVA groups based on their median GSVA scores. We conducted Cox regression analysis on GSVA scores and plotted prognostic correlation graphs using the “survival” and “survminer” packages. Furthermore, following the method of Akbani et al. [21,22], we determined the pathway activity score (PAS) in tumor samples obtained from The Cancer Proteome Atlas (TCPA) database using reverse phase protein array (RPPA) technology. Next, we compared the differences in the PAS scores. Additionally, we investigated the correlation between the GSVA and PAS scores. We used the correlation coefficient to determine whether the DRGs had inhibitory or activating effects on tumor pathways.

### 2.7. Analysis of the Immune Microenvironment and Sensitivity to Antitumor Drugs

First, we used the “ESTIMATE” method to calculate tumor microenvironment (TME) scores in pan-cancer, including stromal, immune, and ESTIMATE scores, based on the expression levels of DRGs. Subsequently, we conducted Spearman’s correlation analysis using the “corplot” package to analyze and visualize the correlation between the mRNA expression levels of DRGs and TME scores. Next, we used the Kruskal–Wallis test to assess variations in DRG expression levels across six distinct immune subtypes. Additionally, we collected relevant data on commonly used chemotherapeutic drugs from the GDSC and CTRP databases. The correlation between DRG expression and the semi-inhibitory concentration (IC50) values of each drug [23] was calculated using the “pRRophetic” package.

### 2.8. Construction and Validation of a Prognostic Model for Liver Cancer

Initially, we performed Cox regression analysis on the 39 DRGs using the “rms” package. Next, we used the least absolute shrinkage and selection operator (LASSO) and multivariate Cox regression methods to analyze 18 DRGs that showed prognostic significance. The analyses identified signature genes and their corresponding regression coefficients, which were used to calculate the risk score (RS). The formula for RS calculation is as follows: RS = β1 × X1 + … + βn × Xn, where X1, …, Xn represent the expression levels of each included gene, and β1, …, βn represent their respective regression coefficients obtained from LASSO regression. Patients with liver cancer were categorized into high- and low-risk groups, according to the median RS. The “survminer” packages were used for survival analysis and visualization of KM curves in the two groups. We used the “survivalROC” package to generate ROC curves for the 1-, 3-, and 5-year survival rates of patients with liver cancer. The AUC was calculated to evaluate the accuracy of the model. Additionally, we repeated the above analysis using the GSE14520 dataset to validate the accuracy of the model. Finally, we integrated the basic information, clinical data, and RS of patients with liver cancer to construct a nomogram scoring system using the “rms” package to predict the survival time of patients with liver cancer. We further evaluated the consistency between actual values and model predictions by generating calibration curves using the “calibrate” function.

### 2.9. Single Cell Analysis

We analyzed the expression patterns of the key gene *RPN1* at the single cell level using eight common solid tumor single cell databases. First, we used the “Seurat” package for data filtering of single cell datasets. Cells were filtered based on gene expression counts ranging from 200 to 3500 and mitochondrial gene expression percentages of >20%. Next, the “FindVariableFeatures” function in the Seurat package was used to select the top 2000 genes with the highest variability for subsequent clustering. Canonical correlation analysis was used to remove batch effects from the data. Then, the “RunPCA” function was used to perform principal component analysis (PCA). Subsequently, we used “FindNeighbors,” “FindClusters,” and “FindAllMarkers” functions to perform cell clustering and sub-clustering to identify marker genes. Finally, we used the “singleR” package to annotate single cell subgroups. The “Dimplot,” “Featureplot,” and “Dotplot” functions were used to visually represent the expression patterns of *RPN1* across different tumor cell subgroups.

### 2.10. Cell Culture and Western Blotting

HepG2, Huh7, and LM3 HCC cells were cultured in RPMI-1640 medium supplemented with 2 mM L-glutamine and 10% fetal bovine serum (FBS). Cell and tissue lysates were prepared using RIPA buffer (Beyotime, Shanghai, China) containing 1% phenylmethanesulfonyl fluoride (PMSF) (Beyotime). Following centrifugation at 4 °C, the proteins were extracted and quantified using a BCA assay kit (Beyotime). Subsequent steps involved gel preparation, electrophoresis, and transfer, followed by membrane incubation with primary (RPN1, 1:5000, 12894-1-AP, Proteintech, Wuhan, China) and secondary (Anti-rabbit IgG, HRP-linked Antibody, 7074S, CST) antibodies. Immunoreactivity was visualized using ECL Plus (EMD Millipore, Billerica, MA, USA).

### 2.11. Immunohistochemistry

Fresh tissues were fixed in 4% paraformaldehyde and embedded in paraffin for sectioning. Antigen retrieval was performed using a citrate buffer and 3% hydrogen peroxide solution to remove endogenous peroxidases. After washing with phosphate-buffered saline, the sections were blocked with serum and incubated with primary (RPN1, 1:200, 12894-1-AP, Proteintech) and secondary antibodies (MaxVisionTM HRP-Polymer anti-Mouse/Rabbit IHC Kit, KIT-5020). A DAB chromogenic detection kit was used for staining, followed by counterstaining with hematoxylin, dehydration, and clearing. Stained sections were examined under a microscope.

### 2.12. Gene Knockdown Cell Lines

The sgRNA sequences for the target genes were designed. The enzyme digestion system was prepared for digestion of the vector (cas9 vector plasmid 5 μg + BsmB1 3 μL + FastAP 3 μL + buffer 6 μL + ddH_2_O). The products were recovered using the TSP601 DNA Gel Recovery Kit (Tsingke Biotech, Beijing, China). Next, sgRNA was ligated into the vector using T4 ligase. Following transformation and sequencing, plasmids were extracted using the TSP513 Endotoxin-Free Plasmid Maxi Kit (Tsingke Biotech, Beijing, China). Finally, knockdown cell lines were generated by transfection using Lipofectamine 2000 reagent (Beyotime, Shanghai, China).

### 2.13. Cell Viability Assay

HepG2 cells transfected with si-*RPN1* were seeded in a 96-well plate and incubated. Cell viability was assessed at various time intervals using the CCK-8 assay kit (MedChemExpress, Shanghai, China), and a curve was plotted to determine cell viability.

### 2.14. Clone Formation Tests

HepG2 cells transfected with si-*RPN1* were seeded in 6-well plates and incubated for 2 weeks. Cell colonies were observed, and subsequently washed and fixed using PBS and 4% paraformaldehyde, respectively. Crystal violet was used for staining.

### 2.15. EdU Assay

HepG2 cells transfected with si-*RPN1* were incubated with EdU for 3 h. The cells were then fixed with a 4% paraformaldehyde solution. Subsequently, the cells were treated with Apollo solution and 0.5% Triton X-100. DAPI solution was used for staining.

### 2.16. Cell Invasion Assay

Matrigel was applied evenly onto the Transwell chamber and subsequently incubated in a cell culture incubator for gelation. After the Matrigel solidified, cell suspensions were added to each transwell chamber. Following a 24-h incubation period, cells were fixed and stained with 4% paraformaldehyde and crystal violet, respectively.

### 2.17. Statistical Analyses

All statistical analyses and data integration were performed using R, GraphPad, and ImageJ software. Statistical significance was set at *p* < 0.05. Significance levels are denoted as * for *p* < 0.05, ** for *p* < 0.01, and *** for *p* < 0.001.

## 3. Results

### 3.1. Correlation between Dysregulation of DRG Expression and Prognosis

**Figure 1:**
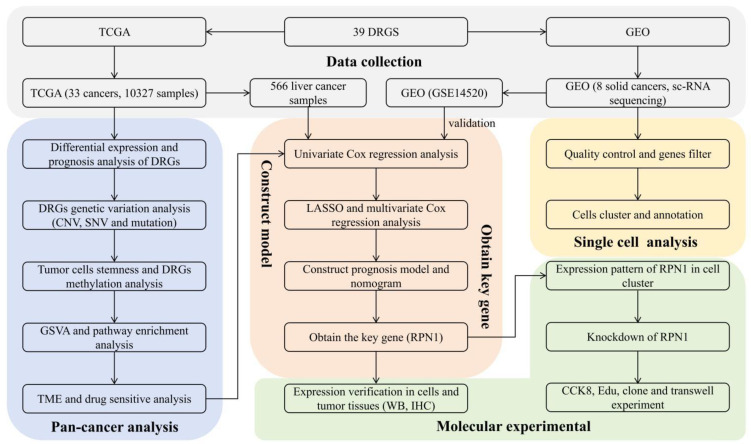
The flowchart of the study.

**Figure 2:**
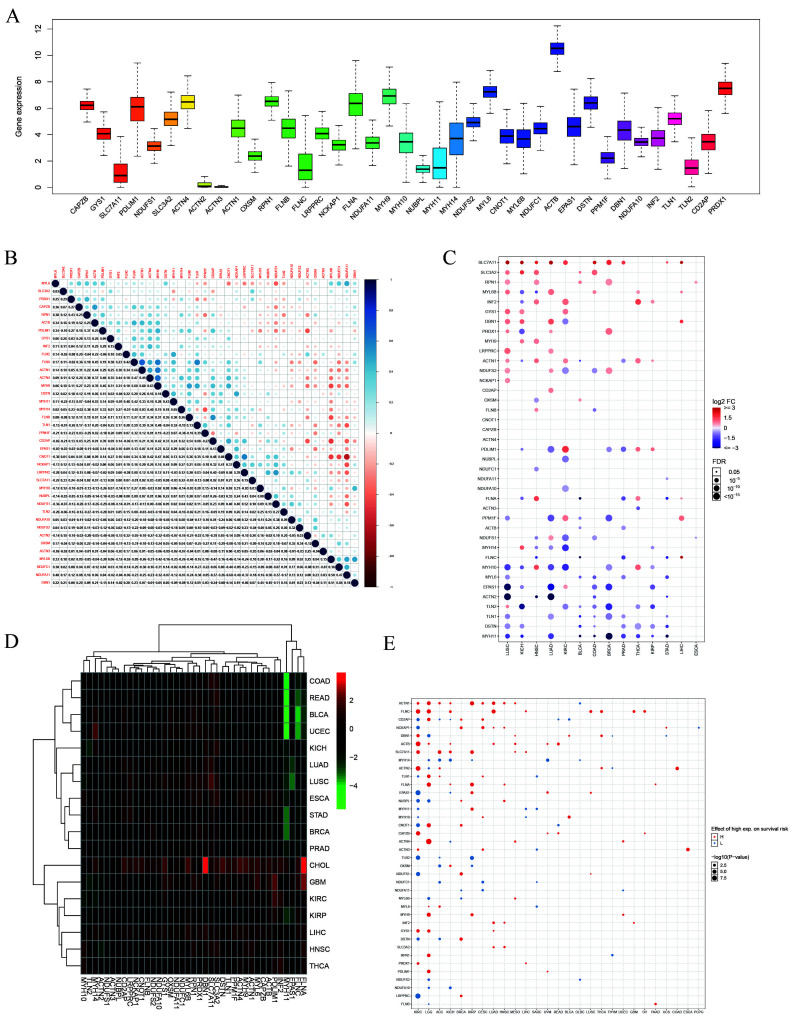
Differential expression and prognostic features of DRGs. (**A**) Expression profiles of DRGs in various cancers. (**B**) Correlation analysis between DRGs, with blue and red indicating positive and negative correlation, respectively. (**C**) Differential mRNA expression of DRGs in normal and tumor tissues. (**D**) Heatmap displaying aberrant expression of DRGs in 18 tumor types, with red and green indicating upregulation and downregulation, respectively. (**E**) Prognostic analysis of DRGs. The size of the dots represents the significance level (*p*-value). Red and blue dots indicate higher and lower prognostic risk, respectively, when the gene is highly expressed.

A flowchart of the study is presented in Figure 1. This study investigated the expression patterns of DRGs across 33 tumor types. In the context of cancer, *ACTB*, *MYH9*, and *MYL6* exhibited elevated expression levels, whereas *SLC7A11*, *ACTN2*, and *ACTN3* exhibited decreased expression levels (Figure 2A). Significant correlations were observed among the DRGs (Figure 2B). *NDUFA11* was strongly negatively correlated with *CNOT1* (r = −0.57, *p* < 0.01), whereas *FLNA* was strongly positively correlated with *ACTN1* (r = 0.64, *p* < 0.01). These findings suggested potential synergistic interactions between these DRGs in tumor regulation. Differential expression analysis revealed that *SLC7A11*, *SLC3A2*, and *RPN1* were significantly upregulated in LUSC, KICH, and HNSC tumor tissues, respectively. In contrast, *TLN2*, *DSTN*, and *MYH11* were significantly downregulated in LUSC, LUAD, and BRCA tumor tissues, respectively (Figure 2C). Heatmap analysis revealed that *DBN1*, *FLNA*, and *MYH11* exhibited the most significant variation in their expression in various cancers. Specifically, *DBN1* and *FLNA* were highly expressed in CHOL tissues, whereas *MYH11* was downregulated in READ, BLCA, and UCEC tissues (Figure 2D). Prognostic correlation analysis revealed a significant association between DRG expression and patient prognosis in various cancer types. For instance, in KIRC, ACC, and KICH, patient survival time was significantly negatively correlated with *SLC7A11* expression and positively correlated with *MYH14* expression (*p* < 0.05) (Figure 2E). Furthermore, the results of Cox regression analysis confirmed a correlation between DRGs and prognosis. *GYS1* exhibited a significant negative correlation with prognosis in KIRC (HR: 1.09–1.93, *p* < 0.05), LUAD (HR: 1.06–1.95, *p* < 0.05), and MESO (HR: 1.13–3.04, *p* < 0.05) (Figure S1).

### 3.2. Genetic Variation in DRGs

**Figure 3:**
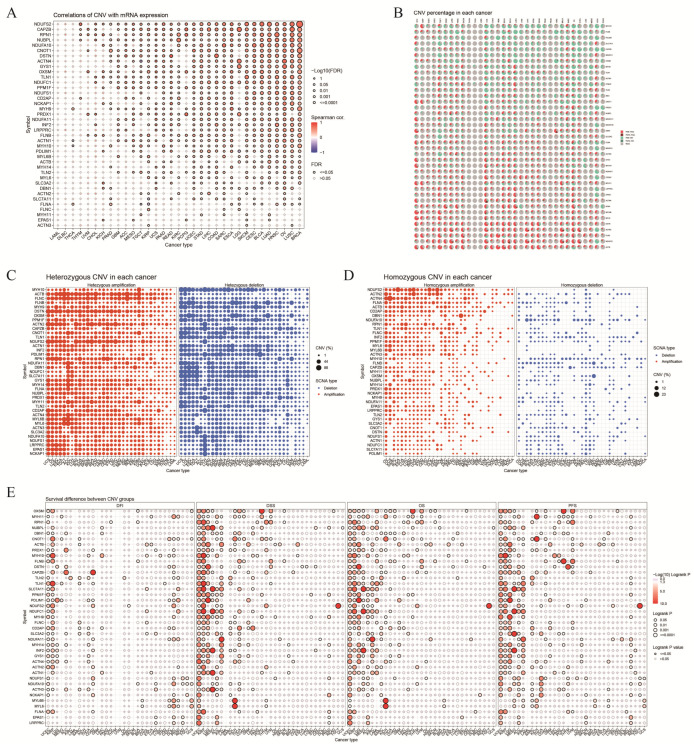
CNV features of the DRGs. (**A**) Correlation between the percentage of CNVs and DRGs mRNA expression in various cancers, with the size and color intensity of the dots representing the *p*-value and the correlation coefficient, respectively. (**B**) Proportions of CNVs in DRGs across various cancers, where Hete Amp, Hete Del, Homo Amp, Homo Del, and None indicate heterozygous amplification, heterozygous deletion, homozygous amplification, homozygous deletion, and no CNV, respectively. (**C**) Proportions of heterozygous amplification and deletions in DRGs across various cancers. (**D**) Proportions of homozygous amplification and deletions in the DRGs across various cancers. (**E**) Prognostic differences between the CNV and wild-type DRG groups. HR values > 1 indicate a risk factor, whereas values < 1 indicate a protective factor. OS: overall survival, DSS: disease-specific survival, DFI: disease-free interval, PFS: progression-free survival.

We conducted a comprehensive analysis of CNV and SNV in the DRGs to investigate their genetic variation characteristics in various cancer types. A strong correlation was observed between DRG expression and CNV in READ, LUSC, OV, HNSC, and LIHC tumors. In READ, *DSTN* expression was significantly correlated with CNV (r = 0.83, *p* < 0.05) (Figure 3A). Furthermore, we investigated the types of CNV and observed that CNV primarily manifested as heterozygous amplification and deletion as well as homozygous amplification and deletion (Figure 3B). In TGCT, *ACTB* showed heterozygous amplification and deletion rates of 81.3% and 1.3%, respectively. In GBM, *PDLIM1* exhibited heterozygous amplification and deletion rates of 0 and 88.2%, respectively (Figure 3C). Homozygous variations were less frequent compared to heterozygous variations. For example, in UCS, *ACTN4* had homozygous amplification and deletion rates of 23.1% and 0%, respectively. In KIRC, *OXSM* exhibited homozygous amplification and deletion rates of 0.19% and 11.2%, respectively (Figure 3D). Additionally, in the prognostic analysis, we identified significant correlations between the CNV status of DRGs and patients’ DPI, DSS, OS, PFS, and other prognostic indicators in several tumors, including UCEC, KIRP, and LGG (log-rank *p*-value < 0.05) (Figure 3E).

**Figure 4:**
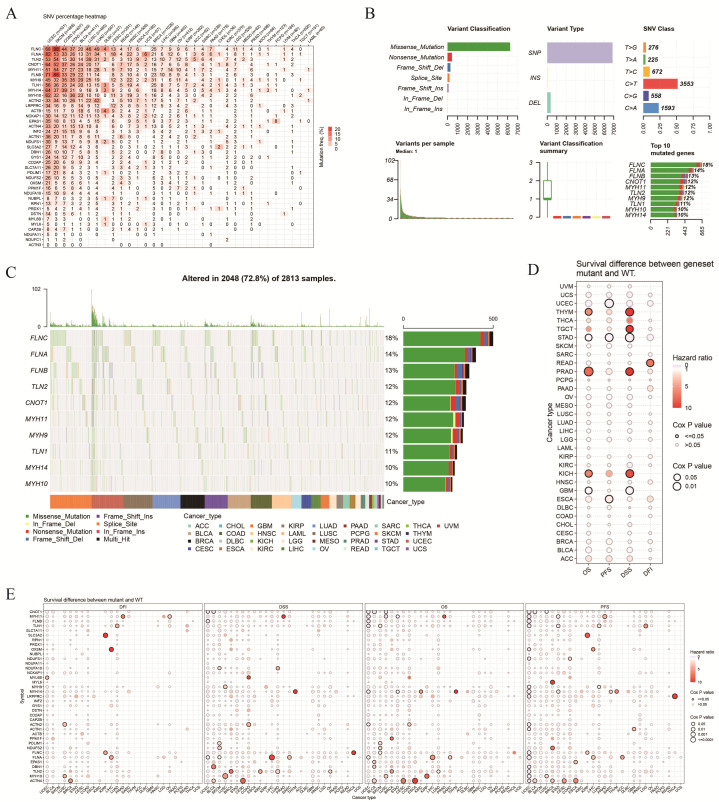
Single nucleotide variation (SNV) features of DRGs. (**A**) Percentage of single nucleotide variations (SNVs) in DRGs across various types of cancers. (**B**) Genetic variation features of DRGs in different types of cancers. (**C**) Waterfall plot showing the top 10 DRGs with the highest mutation frequencies across various types of cancers. (**D**) Correlation between DRGs mutations and prognosis. (**E**) Prognostic differences between patients with SNV mutations and wild-type (WT) patients of DRGs in various types of cancers.

SNV analysis revealed widespread SNVs in the DRGs across various cancers, with UCEC, SKCM, and COAD showing the highest mutation rates (Figure 4A). The DRGs exhibited varying SNV frequencies. For example, in UCEC, *FLNA* had the highest SNV frequency (approximately 98%), whereas *ACTN3* showed minimal variation. Subsequent analysis revealed that the primary classifications of SNVs in the DRGs were missense and nonsense mutations, with SNP being the most common type. These mutations primarily involved cytosine-to-thymine and adenine-to-guanine transitions. The top ten DRGs with the highest mutation frequencies were *FLNC* (18%), *FLNA* (14%), *FLNB* (13%), *TLN2* (12%), *CNOT1* (12%), *MYH11* (12%), *MYH9* (12%), *TLN1* (11%), *MYH14* (10%), and *MYH10* (10%) (Figure 4B,C). Single-factor Cox regression analysis indicated that mutations in the DRGs may significantly affect patient prognosis. Prognostic analysis also revealed significant differences in patient outcomes between the mutant and wild-type groups for certain genes, including *FLNA*, *ACTN2*, and *MYH14* (Figure 4D,E).

### 3.3. Correlation between DRGs and Cell Stemness and Methylation

**Figure 5:**
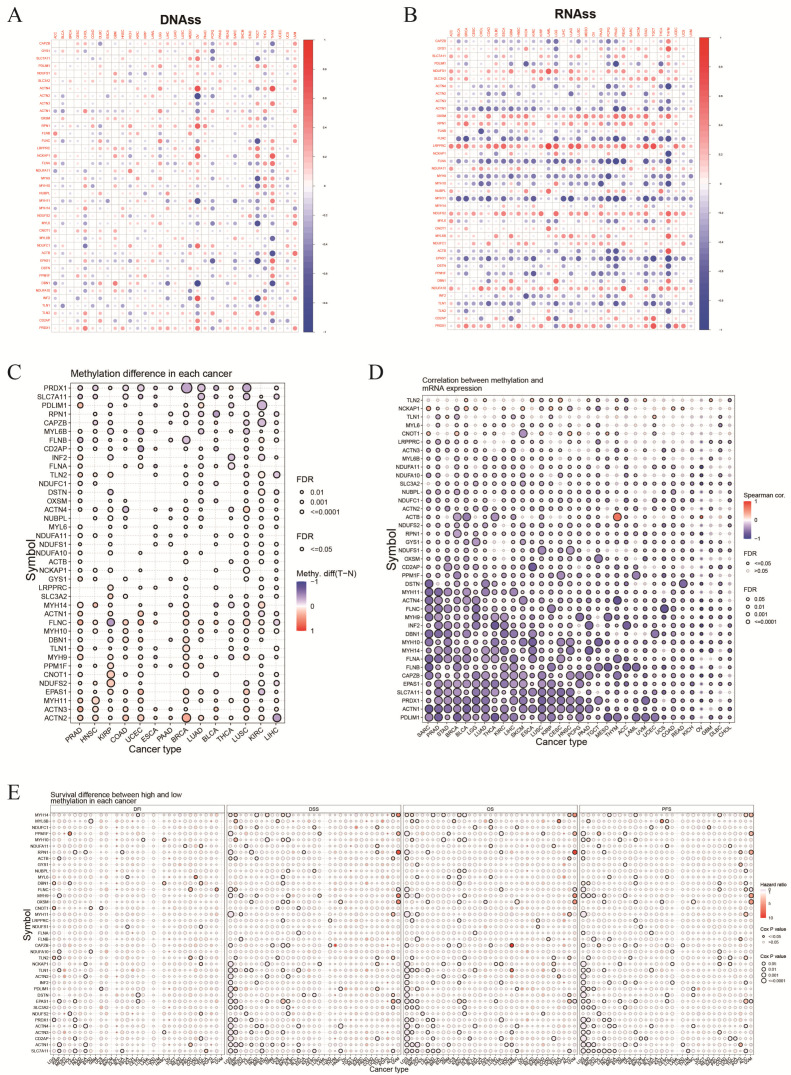
Correlation of DRGs with cell stemness and methylation. (**A**,**B**) Correlation between DRGs mRNA expression and DNAss (DNA methylation) and RNAss (RNA methylation) levels, with red and blue indicating positive and negative correlation, respectively. (**C**) Differential methylation of DRGs between tumor and normal tissues, with red and blue dots indicating increased and decreased methylation in tumors, respectively, and color intensity representing the level of difference. (**D**) Correlation between methylation and mRNA expression levels of DRGs. (**E**) Relationship between the degree of methylation of DRGs and prognosis in various cancer types.

A significant correlation was observed between DRG expression and the DNAss and RNAss scores in various types of tumors, including OV, TGCT, and THCA. In particular, *ACTN4* (r = 0.71, *p* < 0.05), *DBN1* (r = −0.82, *p* < 0.05), and *ACTN2* (r = −0.79, *p* < 0.05) showed the strongest correlation with OV cell stemness (Figure 5A). The RNAss scores in multiple tumors were positively correlated with *LRPPRC*, *RPN1*, and *OXSM* genes and negatively correlated with *ACTN1*, *FLNC*, and *FLNA* genes (*p* < 0.05) (Figure 5B). Additionally, methylation-related analysis showed that genes such as *PRDX1*, *SLC7A11*, and *PDLIM1* had low methylation levels in various cancers, whereas *ACTN2* and *ACTN3* exhibited higher methylation levels (Figure 5C). Moreover, methylation levels were negatively correlated with the mRNA expression of DRGs. An exception was observed in THYM (r = 0.67, *p* < 0.05) and OV (r = 0.72, *p* < 0.05), where *ACTB* expression was positively correlated with methylation levels (Figure 5D). Survival analysis revealed a considerable relationship between the methylation levels of DRGs and the prognosis of patients with tumors. In UVM, high *RPN1* methylation was significantly correlated with DSS (*p* < 0.05, HR = 8.63), OS (*p* < 0.05, HR = 9.53), and PFS (*p* < 0.05, HR = 4.35) of patients, indicating that it is a prognostic risk factor (Figure 5E).

### 3.4. GSVA Score and Enrichment Pathways of the DRGs

GSVA was conducted to determine the GSVA scores for comparing tumor and normal tissues. The results indicated significant differences in the GSVA scores across the 14 tumor types. Except for LIHC, the GSVA scores in BLCA, BRCA, COAD, and ESCA were significantly lower than those in the normal tissues (*p* < 0.05) (Figure S2A). Correlation analysis of tumor-regulating pathways revealed negative correlations between GSVA scores and several pathways, including apoptosis, DNA damage, and the hormone estrogen receptor (ER), across multiple tumors. In contrast, GSVA scores were positively correlated with pathways, including the epithelial–mesenchymal transition (EMT), PI3K/AKT, RAS/MAPK, receptor tyrosine kinase (RTK), and TSC/mTOR signaling pathways (Figure S2B). In TGCT, GSVA scores showed the most significant negative correlation with ER (r = −0.79, *p* < 0.05) and DNA damage (r = −0.73, *p* < 0.05), suggesting the potential involvement of DRGs in these tumor-regulating pathways. Moreover, significant variations in GSVA scores were observed among different molecular subtypes of BLCA, BRCA, GBM, HNSC, LUSC, and STAD (Figure S2C). GSVA scores increased with tumor stage progression, except for ACC and HNSC (Figure S2D), indicating dynamic changes in DRG levels during tumor progression. Similarly, GSVA scores were significantly correlated with prognosis, specifically in BLCA, where they were correlated with DFI (HR = 1.10, *p* < 0.05), DSS (HR = 1.24, *p* < 0.05), OS (HR = 1.47, *p* < 0.05), and PFS (HR = 1.49, *p* < 0.05) (Figure S2E).

### 3.5. TME Score and Antitumor Drug Sensitivity

**Figure 6:**
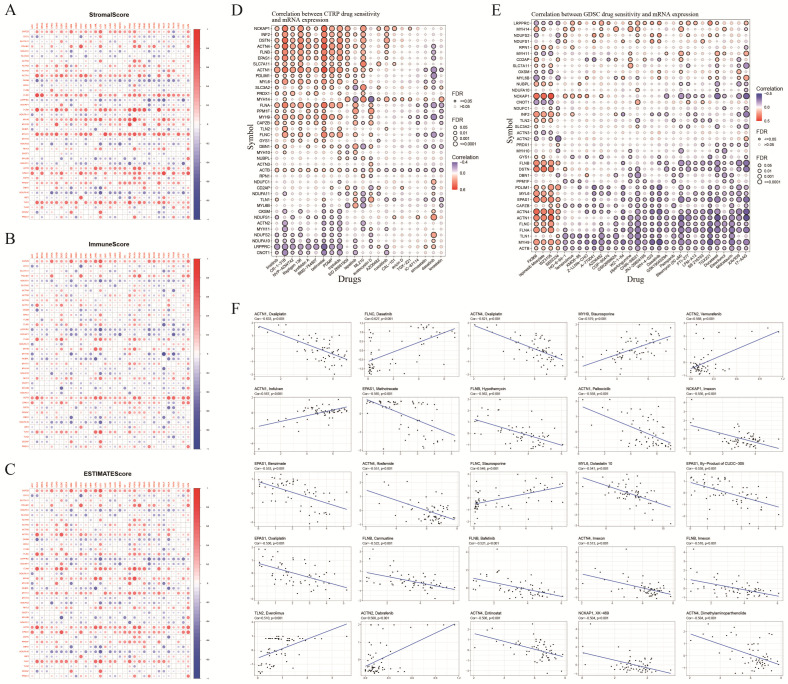
TME score and drug sensitivity analysis. (**A**–**C**) Correlation analysis between mRNA expression of DRGs and stromal, immune, and ESTIMATE scores. (**D**,**E**) Correlation analysis between IC50 values of antitumor drugs and DRGs based on CTPR and GDSC databases, where red and blue indicate positive and negative correlation, respectively. (**F**) Top 25 antitumor drugs with the highest correlation with DRGs.

TME correlation analysis revealed significant correlations between most DRGs in pan-cancer and stromal, immune, and ESTIMATE scores. Stromal score analysis indicated strong correlations between *MYH11*, *FLNA*, *TLN1*, *CAPZB*, and *ACTB* (Figure 6A). Additionally, in the immune and ESTIMATE scores, *CAPZB*, *FLNA*, and *ACTB* showed strong positive correlations, particularly in LGG, BLCA, and DLBC (Figure 6B,C). These findings suggest that DRGs contribute to TME heterogeneity and potentially regulate immune cell infiltration in tumor tissues. Furthermore, we conducted antitumor drug sensitivity analysis to identify potential targeted drugs for DRGs. The findings indicated a positive correlation between the DRGs and IC50 values for CR-1-31B, repligen 136, and belinostat in the CTRP database. *NCKAP1*, *DSTN*, *ACTN4*, *FLNB*, and *EPAS1* were identified as potential target genes (Figure 6D). In the GDSC database, *NCKAP1* positively correlated with the IC50 value of WZ3105, and negatively correlated with that of 17-AGG. *ACTN1* and *ACTN4* were both positively correlated with FK866, ispinensib mesylate, and WZ3105, and negatively correlated with 17-AGG (Figure 6E). Figure 6F presents the top 25 target genes and drugs based on the absolute values of correlation coefficients, including *ACTN1* with Oxaliplatin (r = −0.633, *p* < 0.05), *FLNC* with Dasatinib (r = 0.627, *p* < 0.05), and *ACTN4* with Oxaliplatin (r = −0.621, *p* < 0.05) (Figure 6F).

### 3.6. Prognostic Model and RPN1 Expression in Cell Subsets

**Figure 7:**
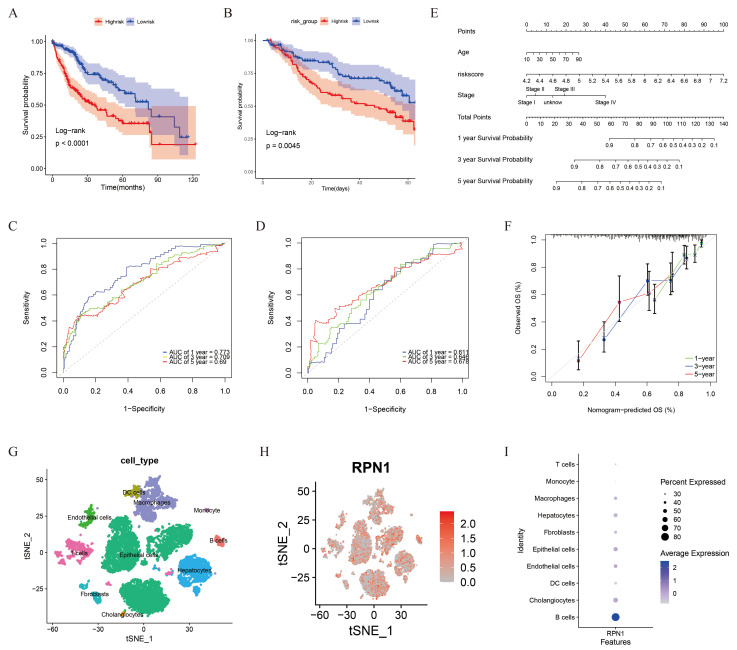
Construction of hepatocellular carcinoma prognostic model and single cell analysis. (**A**) Survival analysis between high- and low-risk groups in TCGA database. (**B**) Survival analysis between high- and low-risk groups in the GSE14520 dataset. (**C**) ROC curve for RS predicting OS in TCGA dataset. (**D**) ROC curve of the GSE14520 dataset. (**E**) Nomogram predicting patient OS. (**F**) Calibration curve to evaluate nomogram accuracy. (**G**) t-SNE plot of dimension reduction and clustering in the GSE138709 dataset. (**H**) Feature plot showing the expression of *RPN1* in GSE138709. (**I**) Dotplot displaying the expression of *RPN1* in the different subgroups of GSE138709.

To investigate the prognostic ability of DRGs in predicting the outcomes of patients with liver cancer, we developed a disulfidptosis model for LIHC. Cox regression analysis identified 18 DRGs that were significantly associated with OS (Table S3). LASSO and multivariate Cox regression analyses identified five independent DRGs and their corresponding coefficients that were significantly associated with prognosis (Figure S3 and Table S4). Survival analysis revealed a significant difference in OS between the two groups (HR: 0.41, 95% CI: 0.289–0.593, *p* < 0.001) (Figure 7A). This finding was validated using the GSE14520 dataset (HR: 0.54, 95% CI: 0.351–0.832, *p* < 0.01) (Figure 7B). ROC curves were generated to evaluate the sensitivity of RS in predicting the 1-, 3-, and 5-year OS in the test (TCGA dataset) and validation (GSE14520 dataset) groups. The AUC values for the test group were 0.773 (95% CI: 0.697–0.830), 0.709 (95% CI: 0.631–0.778), and 0.690 (95% CI: 0.597–0.776), respectively, while those for the validation group were 0.611 (95% CI: 0.499–0.716), 0.646 (95% CI: 0.517–0.693), and 0.678 (95% CI: 0.504–0.739), respectively (Figure 7C,D). Age, RS, and stage were identified as significant risk factors affecting the prognosis of patients with liver cancer (*p* < 0.05). Based on these findings, we developed a prognostic scoring system to accurately predict the outcomes of patients with liver cancer (Figure 7E). The calibration diagram further validated the accuracy of the system model (Figure 7F).

Furthermore, our analysis of this model revealed that RPN1 demonstrated promising prognostic capabilities in pan-cancer and liver cancer. Therefore, we further analyzed single cell data from multiple solid tumors. These findings highlight the ability to stratify liver cancer tissue into ten distinct cell subtypes based on well-established marker genes. Endothelial cells expressed ENG and vWF; macrophages expressed CD168 and CD63; epithelial cells expressed EPCAM and KRT19; monocytes expressed CD14; hepatocytes expressed APOC3, FABP1, and APOA1; cholangiocytes expressed FYXD2, TM4SF4, and ANXA4; fibroblasts expressed ACTA2 and COL1A2; T cells expressed CD2, CD3D, and CD3E; B cells expressed CD79A and MS4A1; and dendritic cells expressed CLEC9A and CD1C (Figure 7G). RPN1 was predominantly expressed in B cells within liver cancer tissue (Figure 7H,I). However, its expression pattern varied among different tumors. RPN1 was primarily expressed in colon-cancer-associated macrophages. RPN1 expression was higher in endothelial cells from lung adenocarcinoma, renal clear cell carcinoma, esophageal adenocarcinoma, and gastric cancer. Conversely, in breast and pancreatic cancers, RPN1 expression was higher in epithelial cells (Figure S4A–G). RPN1 may contribute to the progression and immune response of tumors by affecting proliferation, migration, and immune regulation of specific cell subtypes.

### 3.7. RPN1 Knockdown Promoted LIHC Cell Proliferation and Invasion

**Figure 8:**
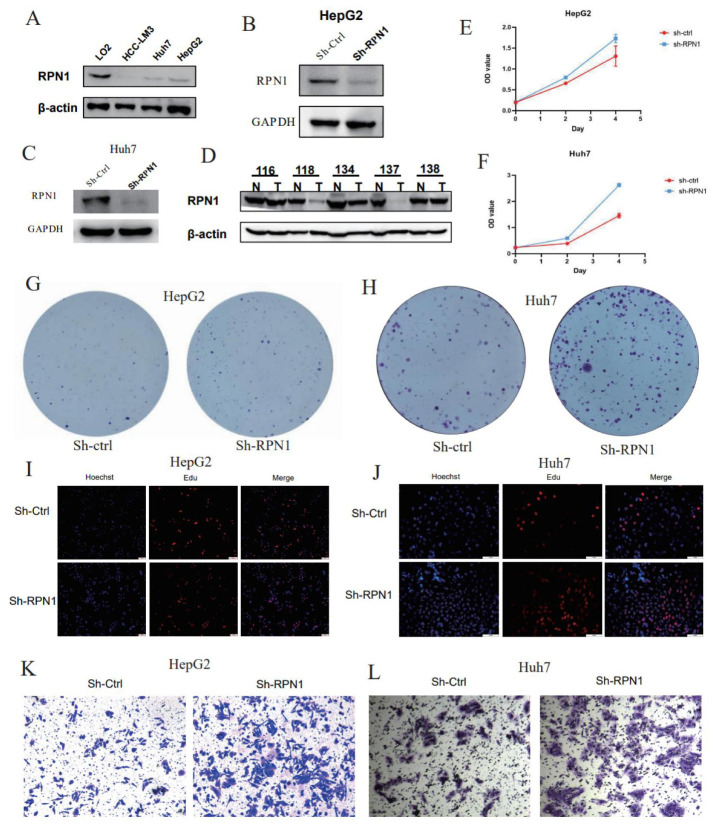
Expression and functional experiments of RPN1 in hepatocellular carcinoma. (**A**) Western blotting analysis of RPN1 levels in LM3, Huh7, HepG2 cell lines. (**B**,**C**) Western blot to demonstrate the knockdown efficiency of RPN1 in HepG2 and Huh 7 cell lines. (**D**) Western blotting analysis of RPN1 levels in tumor and normal tissues. (**E**,**F**) Cell proliferation assay of HepG2 and Huh 7 cell lines transfected with control or si-RPN1 using CCK8. (**G**,**H**) Colony formation assay of HepG2 and Huh 7 cell lines transfected with control or si-RPN1. (**I**,**J**) Edu assay to assess cell proliferation. (**K**,**L**) Transwell assay to evaluate cell metastasis. scale bar = 50 μm.

We conducted a series of experiments to investigate the molecular function of RPN1 in liver cancer. First, we measured RPN1 expression in three LIHC cell lines and found that RPN1 was downregulated in tumor cells compared to normal cells (Figure 8A). Western blot analysis revealed relatively lower RPN1 expression in tumor tissues than in adjacent normal tissues (Figure 8D). Immunohistochemistry (IHC) results indicated cytoplasmic localization of RPN1, supporting its established function as an N-oligosaccharyl transferase in the endoplasmic reticulum (Figure S5A). Next, we successfully knocked down *RPN1* in HepG2 and Huh 7 liver cancer cells (Figure 8B,C). CCK8 experiments demonstrated that *RPN1* knockdown increased the proliferation of HepG2 and Huh 7 liver cancer cells (*p* < 0.05) (Figure 8E,F). The sh-*RPN1* group exhibited a significantly higher number of cell colonies than the control group in colony formation assays, indicating that *RPN1* knockdown enhanced cell proliferation and colony-forming abilities (*p* < 0.05) (Figure 8G,H and Figure S5C,F). EdU staining results showed a significant increase in the percentage of EdU-positive cells in the sh-*RPN1* group compared to the control group (*p* < 0.05) (Figure 8I,J and Figure S5B,E). Transwell assays revealed a significant increase in cell migration across the porous membrane in the sh-*RPN1* group compared to the control group, suggesting that *RPN1* knockdown promoted cell invasion (*p* < 0.05) (Figure 8K,L and Figure S5D,G). These findings suggest that RPN1 may be involved in regulating liver cancer progression and could serve as a promising target for therapeutic interventions in liver cancer.

## 4. Discussion

Several studies have demonstrated the significant involvement of various forms of PCD, including ferroptosis, pyroptosis, and cuproptosis, in the initiation, progression, and immune response of tumors [24,25,26]. However, disulfidptosis, a novel mode of PCD, has not been extensively explored. Therefore, gaining a comprehensive understanding of its molecular characteristics and functions in cancer, specifically liver cancer, is crucial. Therefore, this study comprehensively analyzed the multi-omics characteristics of disulfidptosis in a pan-cancer context, identified hub DRGs, and investigated and validated the specific functions of these critical genes in liver cancer.

Aberrant gene expression and genetic variation are the primary factors contributing to tumor cell dysfunction. Similarly, disulfidptosis activation is dependent on the dysregulated expression of DRGs, particularly the overexpression of *SLC7A11* [11]. In this study, we identified several DRGs with dysregulated expression across all cancer types and found a significant association between these DRGs and tumor prognosis. Furthermore, these DRGs exhibited significant CNVs and SNVs in tumor tissues. Studies suggest that these genetic alterations, such as missense mutations and single-nucleotide substitutions, regulate gene expression and cancer progression [27,28]. This phenomenon was also evident in the present study. FLN family members (*FLNC*, *FLNA*, and *FLNB*) had the highest mutation frequency, primarily characterized by cytosine-to-thymine conversion. This implies that they may serve as potential biomarkers or therapeutic targets for tumors. Ghaffari et al. [29] identified significantly amplified *BIRC5,* due to CNV, as a potential biomarker for early breast cancer screening. Additionally, single-nucleotide polymorphisms (SNPs) have a significant influence on tumor treatment responses. Raida et al. [30] found that polymorphisms in dihydropyrimidine dehydrogenase (*DPYD*) could cause severe toxicity to 5-fluorouracil in cancer patients.

Furthermore, DNA methylation plays a crucial role in regulating gene expression and cancer progression. High methylation of CpG islands in DNA promoters or enhancers typically leads to transcriptional silencing, resulting in negative regulation [31]. This is consistent with the findings of this study, where a significant negative correlation was observed between methylation and the expression of DRGs in pan-cancer. In thymic carcinoma, *ACTB* expression was positively correlated with methylation levels. This could be due to the increased methylation of other DNA regions or the potential rarity of *ACTB* as a tissue-specific gene. For example, reduced methylation of a family of cancer/testis (CT) antigen MAGE promoters in colorectal cancer leads to abnormal gene expression [32]. In addition, DNA methylation has been implicated in the initiation and progression of cancer and is strongly associated with patient prognosis. A study on breast cancer revealed that methylation sites and levels were positively correlated with tumor size, grade, lymph node invasion, and triple-negative status [33]. Its primary mechanism of action in promoting cancer progression is by affecting the methylation status of genes involved in cancer promotion, inhibition, and cell adhesion, thus regulating chromosomal stability and gene expression [34]. In contrast to genetic mutations, epigenetic modifications are reversible. Therefore, DNA demethylation is an important therapeutic strategy for cancer treatment. Azacitidine, for example, has been shown to decrease DNA methylation levels by inhibiting DNA methyltransferases (DNMTs), thereby inhibiting disease progression [35]. However, this mechanism primarily occurs during DNA replication and transcription, resulting in significant side effects that limit its effectiveness in solid tumors [36]. The antisense oligonucleotide MG98 has shown promising results in clinical trials by selectively targeting DNMTs at the translational level in solid tumors [37].

Although the relationship between disulfidptosis and tumor cell glucose metabolism has been established, its association with TME remains elusive. TME encompasses a range of immune cells, including both innate and adaptive cells, which play crucial roles in the immune response against tumors through various mechanisms. Metabolic reprogramming in the TME fulfills the energy demands for tumor cell proliferation and migration while also activating intrinsic and systemic immune responses outside the TME [38]. *SLC7A11* is a key gene involved in disulfidptosis. Studies have shown that it regulates cellular glucose and glutamine uptake, thereby activating the mTOR/AKT signaling pathway and promoting tumor progression [39]. This suggests that disulfidptosis may be involved in the immune response within tumor tissues. Our findings also revealed significant variations in the gene set variation scores of DRGs across multiple tumors. Pathway enrichment analysis primarily identified the apoptosis, EMT, PI3K/AKT, ROS/MAPK, and mTOR/TSC signaling pathways. This finding provides additional evidence supporting the role of disulfidptosis in regulating immune responses and tumor progression mechanisms. Wang et al. [40] identified two immune checkpoint inhibitors (ICGs), cluster of differentiation 276 (CD276) and CD80, which are closely related to breast cancer progression and immune response. Additionally, in pan-cancer, beta-actin (*ACTB*) showed the highest immune and ESTIMATE scores. ACTB is a critical protein involved in cytoskeletal contraction during disulfidptosis and has traditionally been used as a reference gene. However, increasing evidence suggests that *ACTB* is overexpressed in various cancers and is involved in tumor invasion and immune regulation. *ACTB* is associated with multiple immune cells, checkpoints, and modulators. *ACTB* knockdown inhibited the migration and invasion of HNSC cells by activating the NF-κB and Wnt/β-catenin signaling pathways [41]. Despite these findings, a comprehensive understanding of the specific immunological function of disulfidptosis in tumors requires further exploration.

In addition, disulfidptosis has shown promising potential for the treatment of certain tumors. The prognostic model developed in this study, based on DRGs and applied to patients with liver cancer, demonstrated a high level of accuracy in predicting patient outcomes. This finding is consistent with the results of Yang et al. [42], who identified DRGs as independent prognostic markers for liver cancer. *SLC7A11* and *LRPPRC* have been identified as key DRGs that influence liver cancer prognosis. In contrast, our regression analysis results revealed a significant association between RPN1 expression and the prognosis of patients with liver cancer. Moreover, in vitro studies have shown that *RPN1* downregulation increases proliferation and invasion of liver cancer cells. This suggests that RPN1 may function as a promising biomarker and therapeutic target in liver cancer.

RPN1, also known as ribophorin 1, is an essential constituent of the OST complex, which is crucial for OST-mediated N-linked glycosylation [43]. N-linked glycosylation occurs during the translocation of proteins to the endoplasmic reticulum. It plays a vital role in maintaining protein stability and in ensuring proper folding [44,45]. Abnormal RPN1 expression can reduce glycosylation levels, causing protein misfolding and increased ERS [46]. Studies have demonstrated that prolonged ERS can induce a transition in cellular conditions from promoting survival to favoring cell death [47]. This process relies on downstream signaling molecules associated with ERS, including PERK, IRE1a, and ATF6. Wang et al. [48] found that IRE1a can activate the JNK signaling pathway to suppress the anti-apoptotic protein BCL2, thereby promoting tumor cell apoptosis. ATF6 promotes apoptosis and disrupts the interaction between cAMP response element-binding protein (CREB) and CREB-regulated transcription coactivator 2 (CRTC2), thereby inhibiting glucose output in liver cells [49]. Interestingly, in glucose- and glutamine-starved tumor cells, RPN1 triggers disulfidptosis and inhibits the hexosamine biosynthetic pathway. This inhibition blocks the synthesis of uridine diphosphate N-acetylglucosamine and ATP, which further limits proper protein folding and exacerbates ERS [50,51]. This suggests a potential association between disulfidptosis and ERS during tumor progression. However, the mechanisms underlying the interaction between disulfidptosis and ERS remain unclear. RPN1 may function as a hub that connects these two processes.

## 5. Conclusions

This study presents a comprehensive analysis of the overall transcriptional and genetic changes associated with disulfidptosis in pan-cancer. Additionally, the correlation between these changes and patient prognosis was investigated. The multi-omics characteristics of DRGs, including their expression, genetic variations, modifications, immune infiltration, TME, and drug sensitivity, were analyzed. Moreover, the significance of *RPN1* as a critical DRG and its molecular function in liver cancer were identified and validated. Furthermore, the potential mechanisms by which RPN1 mediates disulfidptosis and tumor progression were elucidated. Therefore, this study contributes to a deeper understanding of the specific mechanisms of disulfidptosis in cancer and presents a promising therapeutic target for HCC treatment.

## Data Availability

The datasets generated and/or analyzed during the current study are available in the TCGA (https://portal.gdc.cancer.gov/, accessed on 1 December 2023) and Gene Expression Omnibus (GEO) (https://www.ncbi.nlm.nih.gov/geo/, accessed on 1 December 2023).

## Supplementary Materials

The following supporting information can be downloaded at: https://www.mdpi.com/article/10.3390/biom14060677/s1, Figure S1: Forest plot showing the results of univariate Cox regression analysis for DRGs in 33 types of cancers; Figure S2: GSVA Scores and Enriched Pathways of DRGs; Figure S3: LASSO regression analysis and partial likelihood deviance of the prognostic hub genes; Figure S4: Expression characteristics of RPN1 in different cell subtypes of lung adenocarcinoma; Figure S5: A: Immunohistochemistry detection of RPN1 in hepatocellular carcinoma tissues and normal tissues; Table S1: 39 disulfidptosis related genes; Table S2: 33 types of tumors and sample size; Table S3: 18 disulfidptosis genes associated with OS in LIHC; Table S4: Signature genes and their LASSO regression coefficients.

## Abbreviations

TIME: Tumor immune microenvironment; DRGs: Disulfidptosis-related genes; EMT: Epithelial–mesenchymal transition; RPN1: Ribophorin 1; AFP: Alpha-fetoprotein; HCC: Hepatocellular carcinoma; PCD: Programmed cell death; SLC7A11: Solute carrier family 7 member 11; TCGA: The Cancer Genome Atlas; CNV: Copy number variation; SNV: Single nucleotide variation; GDSC: Genomics of Drug Sensitivity in Cancer; CTRP: Cancer Therapeutic Response Portal; GEO: Gene Expression Omnibus; DFI: Disease-free interval; OS: Overall survival; DSS: Disease-specific survival; PFS: Progression-free survival; DNAss: DNA methylation; TCPA: The Cancer Proteome Atlas; TME: Tumor microenvironment; LASSO: Least absolute shrinkage and selection operator; ROC: Receiver operating characteristic; AUC: Area under the curve; PCA: Principal component analysis; SNP: Single nucleotide polymorphisms; DPYD: Dihydropyrimidine dehydrogenase; DNMT: DNA methyltransferases; ICGs: Immune checkpoint inhibitors; CD276: Cluster of differentiation 276; OST: Oligosaccharyltransferase; ERS: Endoplasmic reticulum stress; CREB: cAMP response element-binding protein; CRTC2: CREB-regulated transcription coactivator 2.
