# Supplementary material for: Multi-Omics Insights into Disulfidptosis-Related Genes Reveal RPN1 as a Therapeutic Target for Liver Cancer
Source: Biomolecules. 2024 Jun 10;14(6):677. doi: 10.3390/biom14060677 (PMC11201601; doi:10.3390/biom14060677)
Supplement: Supplementary file 1 [file biomolecules-14-00677-s001.zip › Supplementary tables and figure legends.pdf]

Pan-cancer analysis reveals the multi-omics characteristics of the disulfidptosis-related gene set and identifies RPN1 as a novel therapeutic target for liver cancer

Yan He[1†], Dong Liu[2†], Xutong Li[3], Yunsheng Cheng[4], Katharina Joechle[2], Yue Hu[5], Huihu He[6*], Feng Cao[2*]

Supplementary table and figure legends

Table S1: 39 disulfidptosis related genes
Table S2: 33 types of tumors and sample size
Table S3: 18 disulfidptosis genes associated with OS in LIHC
Table S4: Signature genes and their LASSO regression coefficients.
Figure S1: Forest plot showing the results of univariate Cox regression analysis for DRGs in 33 types of cancers.
Figure S2: GSVA Scores and Enriched Pathways of DRGs. A: Differential GSVA scores between tumor and normal samples in 14 cancer types. B: Correlation between GSVA scores of DRGs and enriched pathways; * $p < 0.05$, # $p < 0.01$. C: Differential GSVA scores among different tumor subtypes. D: Trends in GSVA scores with tumor stage changes in different cancers. E: Differences in survival between patients with high and low GSVA scores.
Figure S3: LASSO regression analysis and partial likelihood deviance of the prognostic hub genes.
Figure S4: Expression characteristics of RPN1 in different cell subtypes of lung adenocarcinoma (A), colon cancer (B), breast cancer (C), clear cell renal cell carcinoma (D), esophageal adenocarcinoma (E), gastric cancer (F), and pancreatic cancer (G).
Figure S5: A: Immunohistochemistry detection of RPN1 in hepatocellular carcinoma tissues and normal tissues. B-D:Comparison of edu positive cells, clone number and invaded cells in HepG2 cells between control group and si-RPN1 group (*: $p < 0.05$; ** $p < 0.01$). E-F:omparison of edu positive cells, clone number and invaded cells in Huh7 cells between control group and si-RPN1 group (*: $p < 0.05$; ** $p < 0.01$).
