# Supplementary material for: Multi-Omics Insights into Disulfidptosis-Related Genes Reveal RPN1 as a Therapeutic Target for Liver Cancer
Source: Biomolecules. 2024 Jun 10;14(6):677. doi: 10.3390/biom14060677 (PMC11201601; doi:10.3390/biom14060677)
Supplement: Supplementary file 1 [file biomolecules-14-00677-s001.zip › Table S1.pdf]

Table S1 39 disulfidptosis related genes

ID	Encoding protein
ACTB	Actin beta
ACTN1	Actinin alpha 1
ACTN2	Actinin alpha 2
ACTN3	Actinin alpha 3
ACTN4	Actinin alpha 4
CAPZB	Capping actin protein of muscle Z-line subunit beta
CD2AP	CD2 associated protein
CNOT1	CCR4-NOT transcription complex subunit 1
DBN1	Drebrin 1
DSTN	Destrin, actin depolymerizing factor
EPAS1	Endothelial PAS domain protein 1
FLNA	Filamin A
FLNB	Filamin B
FLNC	Filamin C
GYS1	Glycogen synthase 1
INF2	Inverted formin 2
LRPPRC	Leucine rich pentatricopeptide repeat containing
MYH10	Myosin heavy chain 10
MYH11	Myosin heavy chain 11
MYH14	Myosin heavy chain 14
MYH9	Myosin heavy chain 9
MYL6	Myosin light chain 6
MYL6B	myosin light chain 6B
NCKAP1	NCK associated protein 1
NDUFA10	NADH:ubiquinone oxidoreductase subunit A10
NDUFA11	NADH:ubiquinone oxidoreductase subunit A11
NDUFC1	NADH:ubiquinone oxidoreductase subunit C1
NDUFS1	NADH:ubiquinone oxidoreductase core subunit S1
NDUFS2	NADH:ubiquinone oxidoreductase core subunit S2
NUBPL	NUBP iron-sulfur cluster assembly factor
OXSM	3-oxoacyl-ACP synthase
PDLIM1	PDZ and LIM domain 1
PPM1F	Protein phosphatase, Mg2+/Mn2+ dependent 1F
PRDX1	Peroxiredoxin 1
RPN1	Ribophorin I
SLC3A2	Solute carrier family 3 member 2
SLC7A11	Solute carrier family 7 member 11
TLN1	Talin 1
TLN2	Talin 2
