# Supplementary material for: Multi-Omics Insights into Disulfidptosis-Related Genes Reveal RPN1 as a Therapeutic Target for Liver Cancer
Source: Biomolecules. 2024 Jun 10;14(6):677. doi: 10.3390/biom14060677 (PMC11201601; doi:10.3390/biom14060677)
Supplement: Supplementary file 1 [file biomolecules-14-00677-s001.zip › Table S2.pdf]

Table S2 33 types of tumors and sample size

Tumor abbreviation	Full name	Tumor sample size	Normal sample size
ACC	Adrenocortical carcinoma	79	0
BLCA	Bladder urothelial carcinoma	411	19
BRCA	Breast invasive carcinoma	1104	113
CESC	Cervical squamous cell carcinoma and endocervical adenocarcinoma	306	3
CHOL	Cholangiocarcinoma	36	9
COAD	Colon adenocarcinoma	471	41
ESCA	Esophageal carcinoma	168	11
GBM	Glioblastoma multiforme	168	5
HNSC	Head and neck squamous cell carcinoma	502	44
KICH	Kidney chromophobe	65	24
KIRP	Kidney renal papillary cell carcinoma	289	32
KIRC	Kidney renal clear cell carcinoma	535	72
LAML	Acute myeloid leukemia	151	0
LGG	Lower grade glioma	529	0
LIHC	Liver hepatocellular carcinoma	374	50
LUAD	Lung adenocarcinoma	526	59
LUSC	Lung squamous cell carcinoma	501	49
DLBC	Lymphoid neoplasm diffuse large B-cell lymphoma	48	0
MESO	Mesothelioma	86	0
OV	Ovarian serous cystadenocarcinoma	379	0
PAAD	Pancreatic adenocarcinoma	178	4
PCPG	Pheochromocytoma and paraganglioma	183	3
PRAD	Prostate adenocarcinoma	499	52
READ	Rectum adenocarcinoma	167	10
SKCM	Skin cutaneous melanoma	471	1
SARC	Sarcoma	267	2
STAD	Stomach adenocarcinoma	375	32
TGCT	Testicular germ cell tumors	156	0
THCA	Thyroid carcinoma	510	50
THYM	Thymoma	119	2
UCS	Uterine carcinosarcoma	56	0
UCEC	Uterine corpus endometrial carcinoma	548	35
UVM	Uveal melanoma	80	0
