# Supplementary material for: Multi-Omics Insights into Disulfidptosis-Related Genes Reveal RPN1 as a Therapeutic Target for Liver Cancer
Source: Biomolecules. 2024 Jun 10;14(6):677. doi: 10.3390/biom14060677 (PMC11201601; doi:10.3390/biom14060677)
Supplement: Supplementary file 1 [file biomolecules-14-00677-s001.zip › Table S3.pdf]

Table S3 18 disulfidptosis genes associated with OS in LIHC

Gene	HR	95%CI lower	95% CI upper	*P* value
SLC7A11	1.461	1.229	1.737	0.000
PRDX1	1.646	1.294	2.093	0.000
LRPPRC	1.983	1.405	2.799	0.000
RPN1	2.116	1.396	3.206	0.000
ACTN3	3.113	1.579	6.135	0.001
FLNC	1.209	1.075	1.360	0.002
INF2	1.463	1.150	1.860	0.002
CAPZB	1.616	1.163	2.245	0.004
NCKAP1	1.522	1.134	2.043	0.005
CD2AP	1.434	1.114	1.846	0.005
MYL6B	1.300	1.078	1.569	0.006
DBN1	1.200	1.044	1.379	0.010
GYS1	1.338	1.047	1.710	0.020
DSTN	1.337	1.027	1.739	0.031
ACTB	1.383	1.028	1.861	0.032
NDUFS2	1.404	1.020	1.932	0.038
SLC3A2	1.295	1.014	1.653	0.038
ACTN1	1.287	1.012	1.636	0.040
