# Supplementary material for: Multi-Omics Insights into Disulfidptosis-Related Genes Reveal RPN1 as a Therapeutic Target for Liver Cancer
Source: Biomolecules. 2024 Jun 10;14(6):677. doi: 10.3390/biom14060677 (PMC11201601; doi:10.3390/biom14060677)
Supplement: Supplementary file 1 [file biomolecules-14-00677-s001.zip › Table S4.pdf]

Table S4 Signature genes and their LASSO regression coefficients.

Gene	Coefficient
MYL6B	0.004
PRDX1	0.282
RPN1	0.22
SLC7A11	0.207
ACTN3	0.877
FLNC	0.006
LRPPRC	0.282
INF2	0.206
