# Supplementary figures and images for: Multi-Omics Insights into Disulfidptosis-Related Genes Reveal RPN1 as a Therapeutic Target for Liver Cancer
Source: Biomolecules. 2024 Jun 10;14(6):677. doi: 10.3390/biom14060677 (PMC11201601; doi:10.3390/biom14060677)

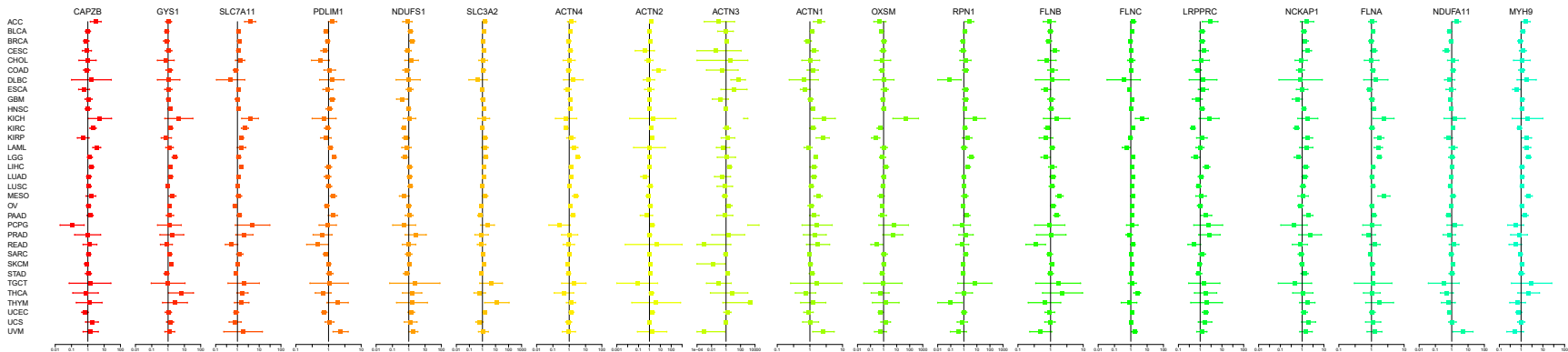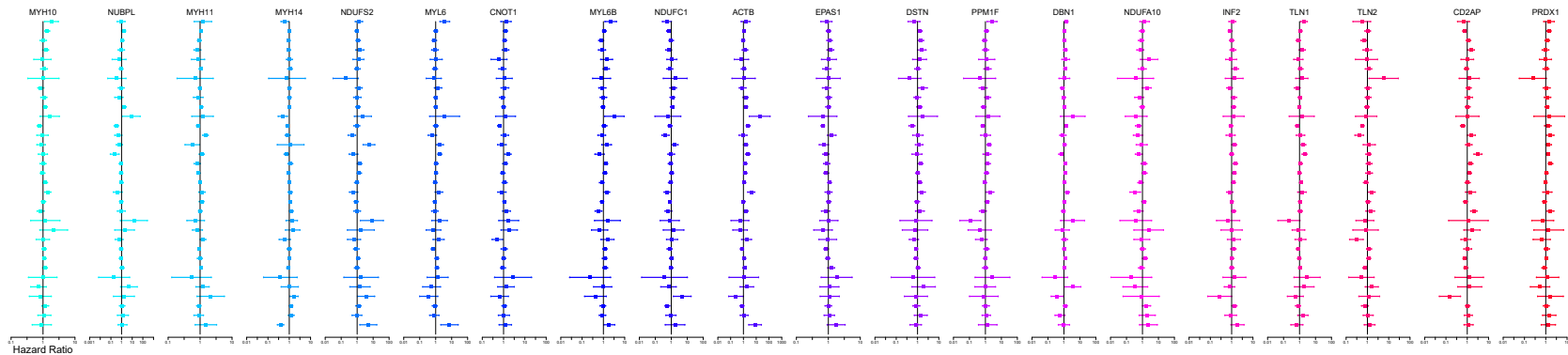

Supplement: Supplementary file 1 [file biomolecules-14-00677-s001.zip › Figure S1.pdf]

A

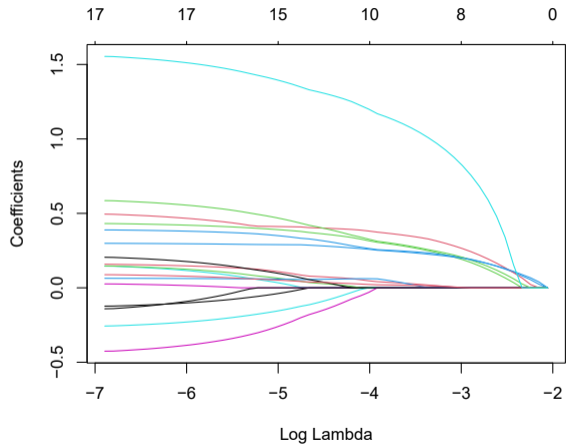

B

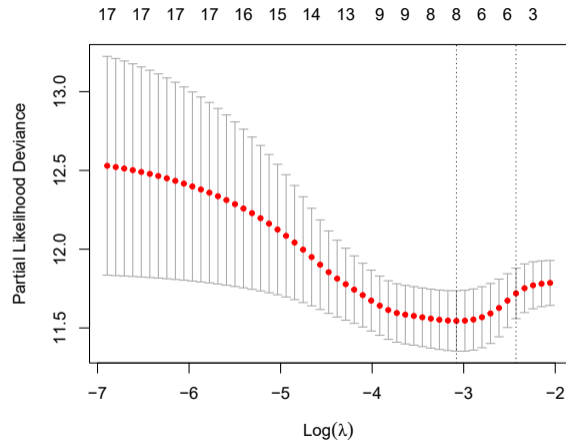

Supplement: Supplementary file 1 [file biomolecules-14-00677-s001.zip › Figure S3.pdf]

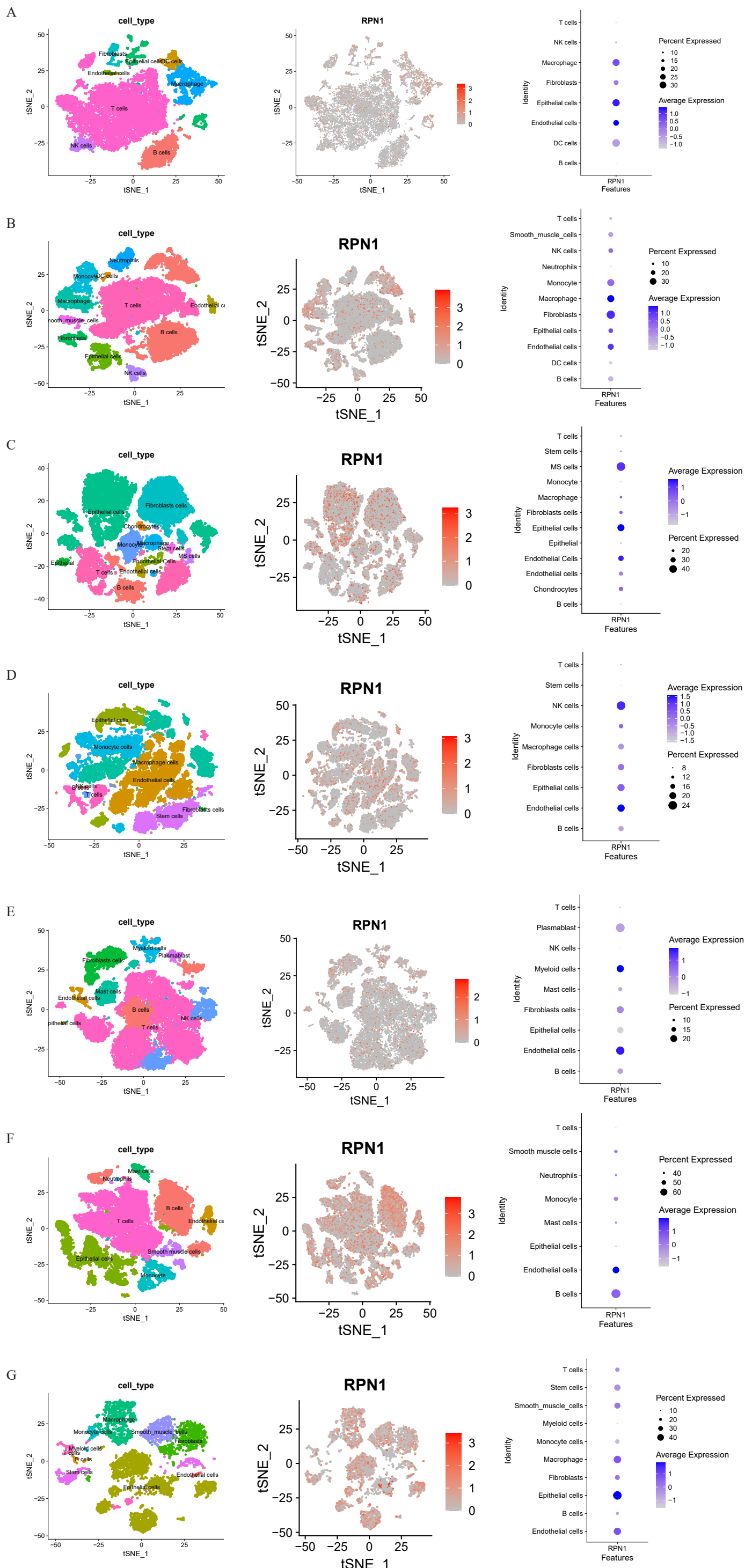

Supplement: Supplementary file 1 [file biomolecules-14-00677-s001.zip › Figure S4.pdf]

# A

## Normal

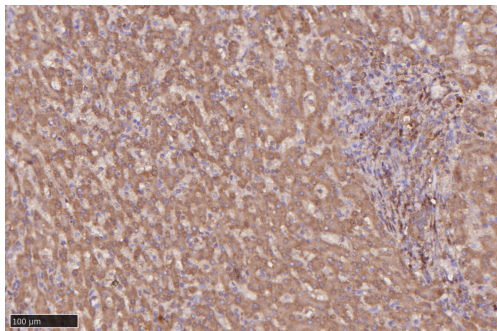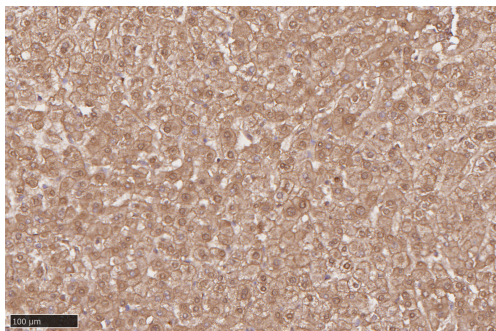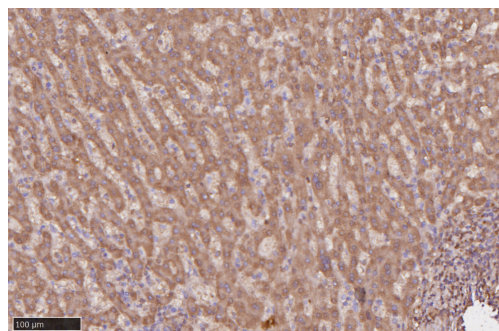

## Tumor

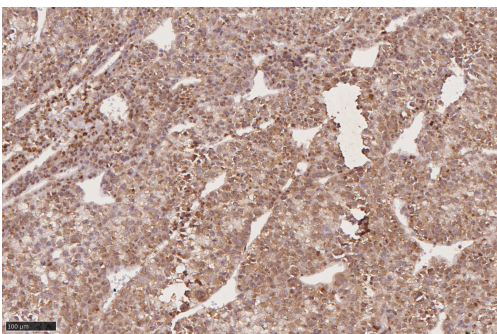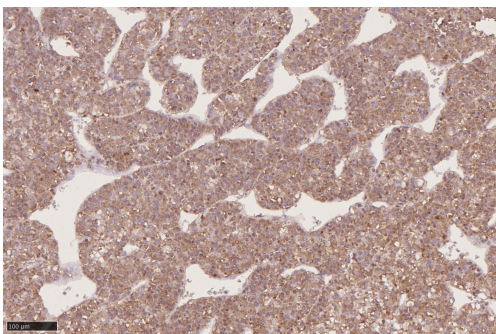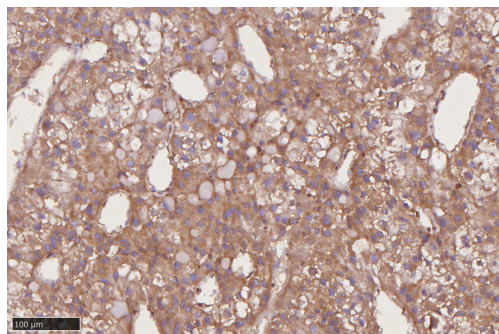

# B

## HepG2

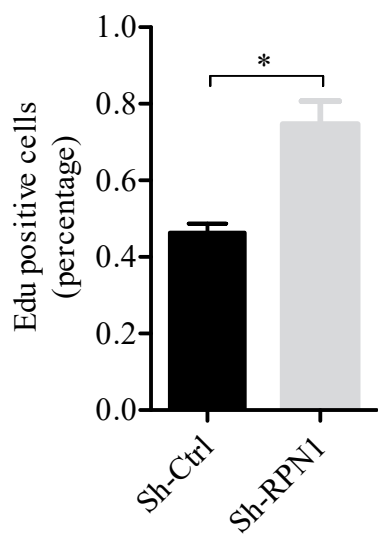

# C

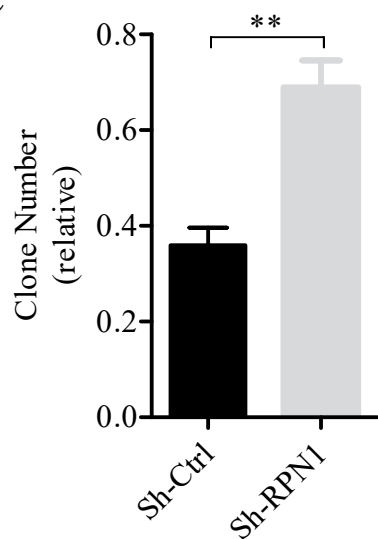

# D

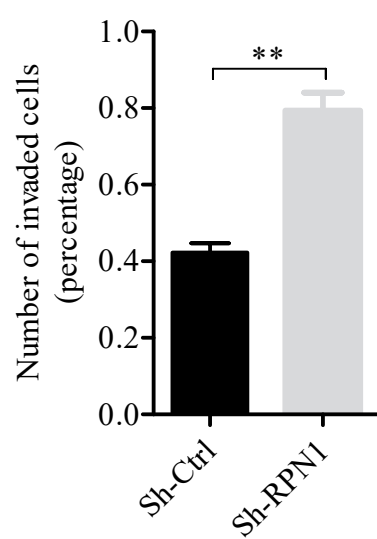

# E

## Huh7

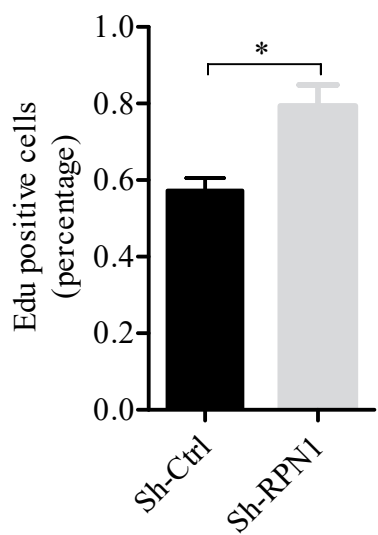

# F

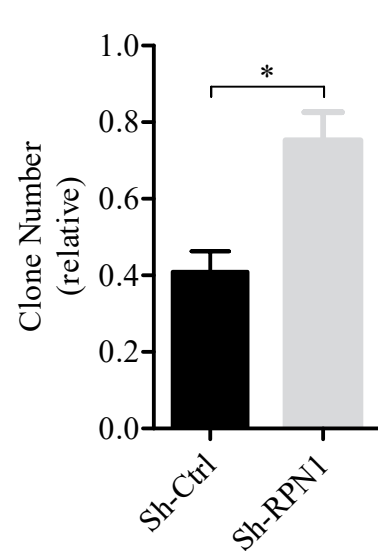

# G

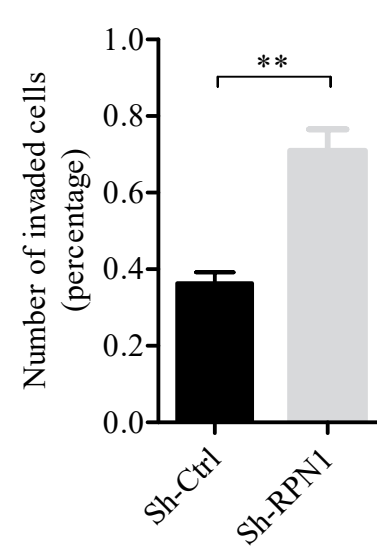

Supplement: Supplementary file 1 [file biomolecules-14-00677-s001.zip › Figure S5.pdf]

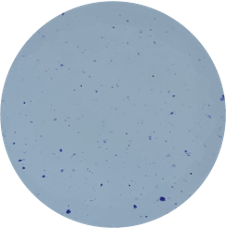

Supplement: Supplementary file 1 [file biomolecules-14-00677-s001.zip › Raw data/HepG/clone/sh-Ctrl.png]

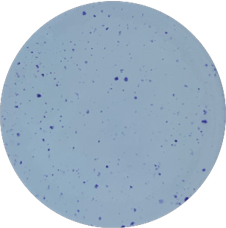

Supplement: Supplementary file 1 [file biomolecules-14-00677-s001.zip › Raw data/HepG/clone/sh-RPN1.png]

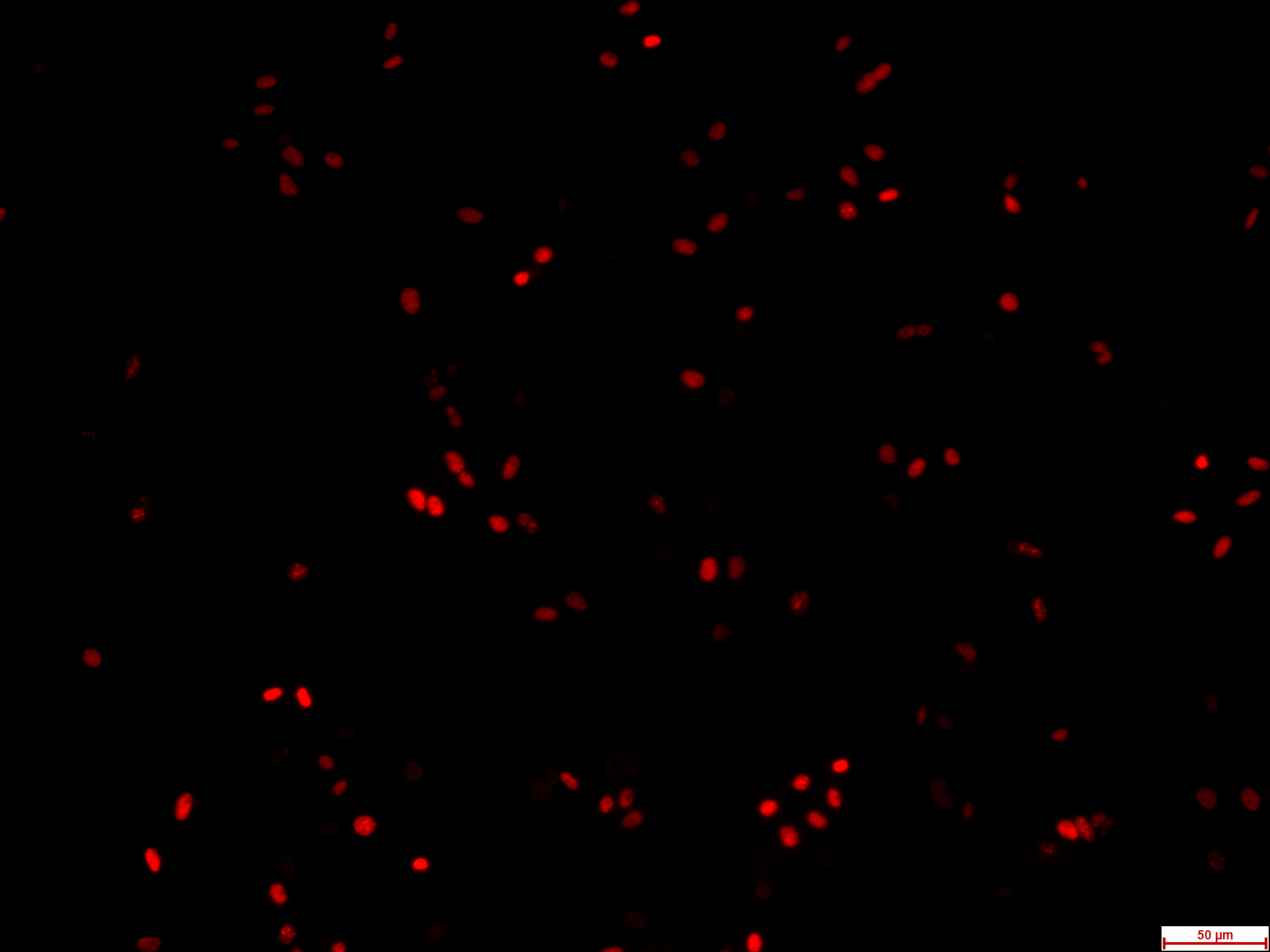

Supplement: Supplementary file 1 [file biomolecules-14-00677-s001.zip › Raw data/HepG/EDU/23.7.10-edu/PK/1.tif]

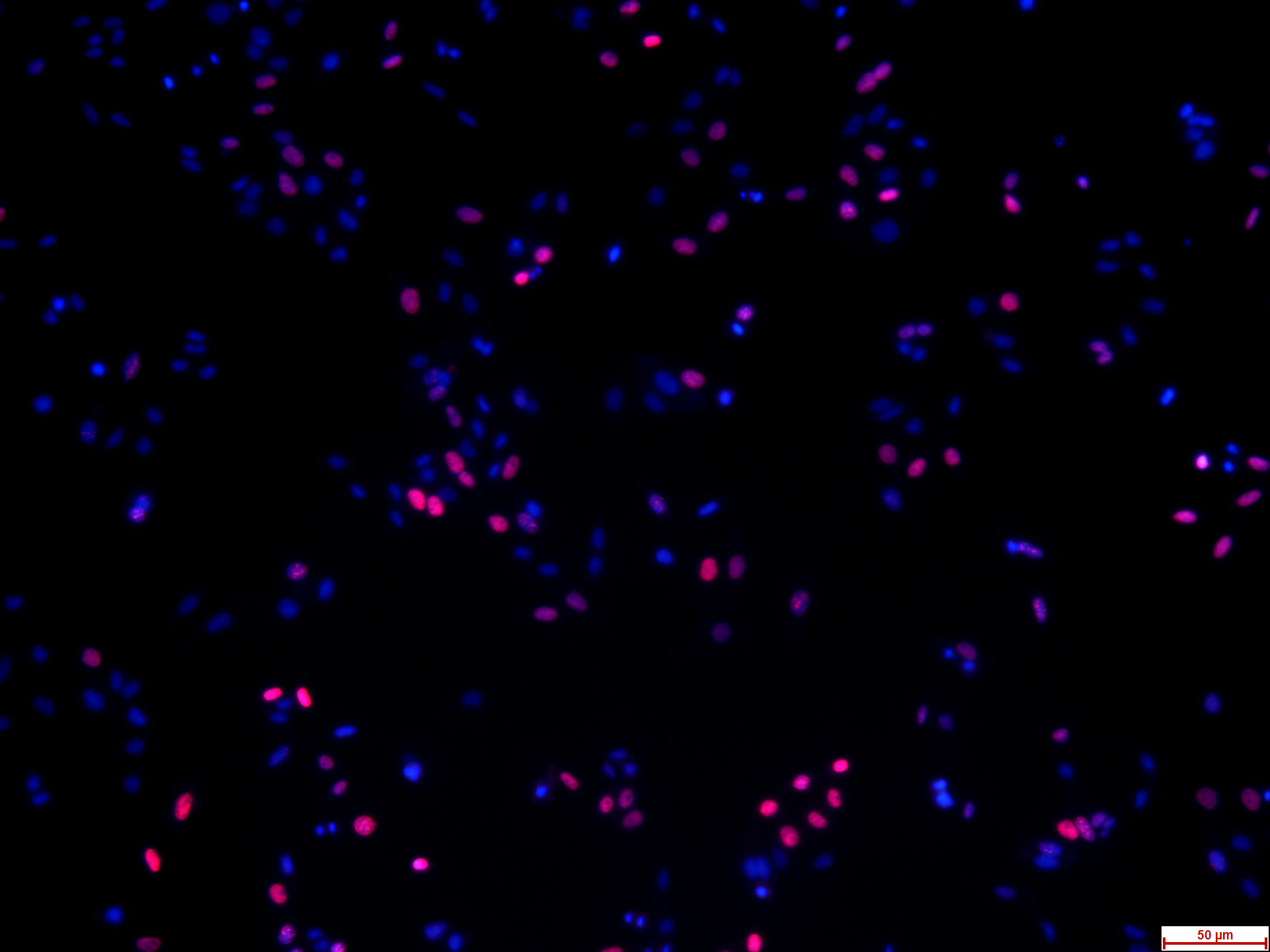

Supplement: Supplementary file 1 [file biomolecules-14-00677-s001.zip › Raw data/HepG/EDU/23.7.10-edu/PK/2.tif]

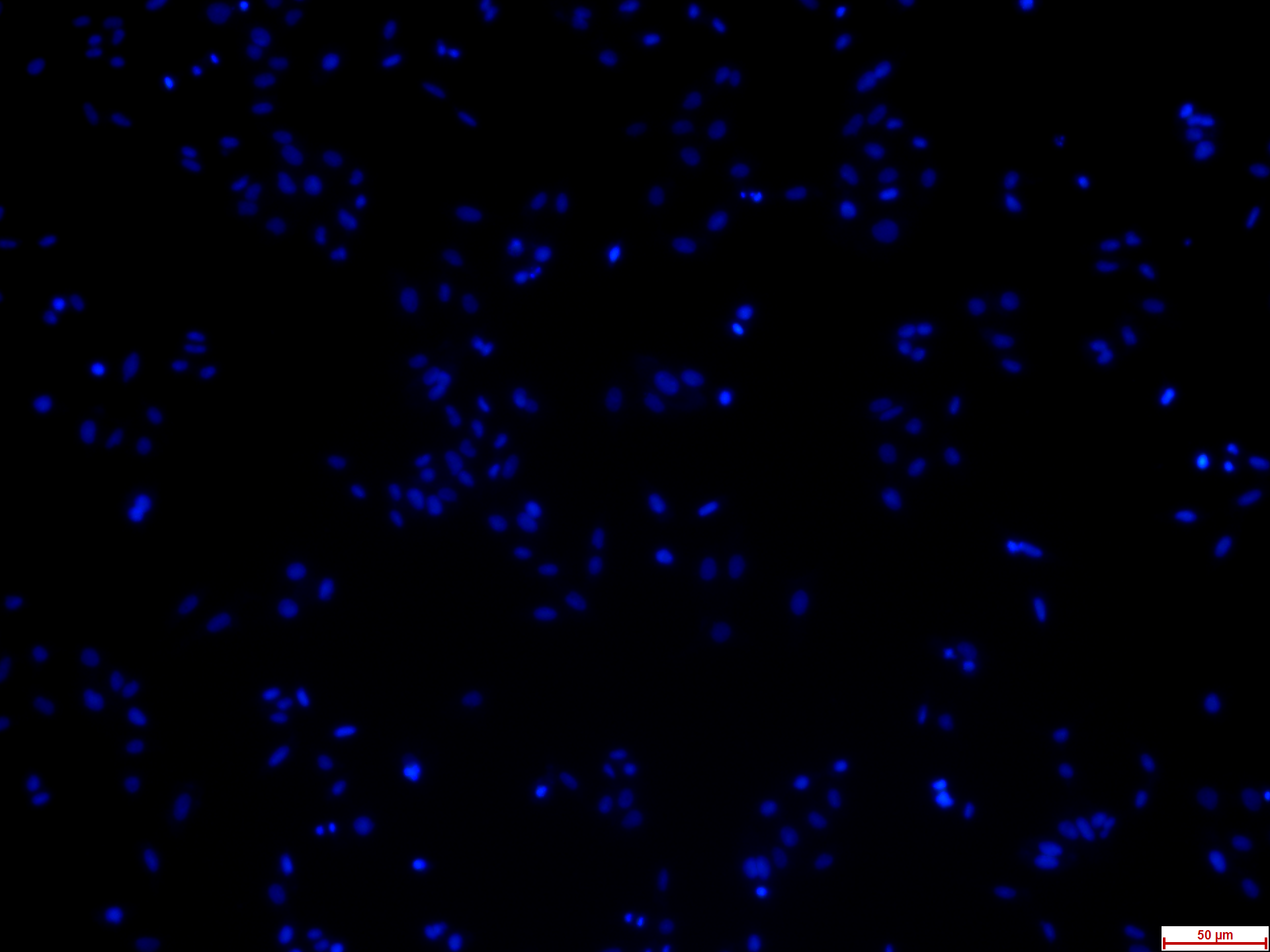

Supplement: Supplementary file 1 [file biomolecules-14-00677-s001.zip › Raw data/HepG/EDU/23.7.10-edu/PK/PLKO.1-2.tif]

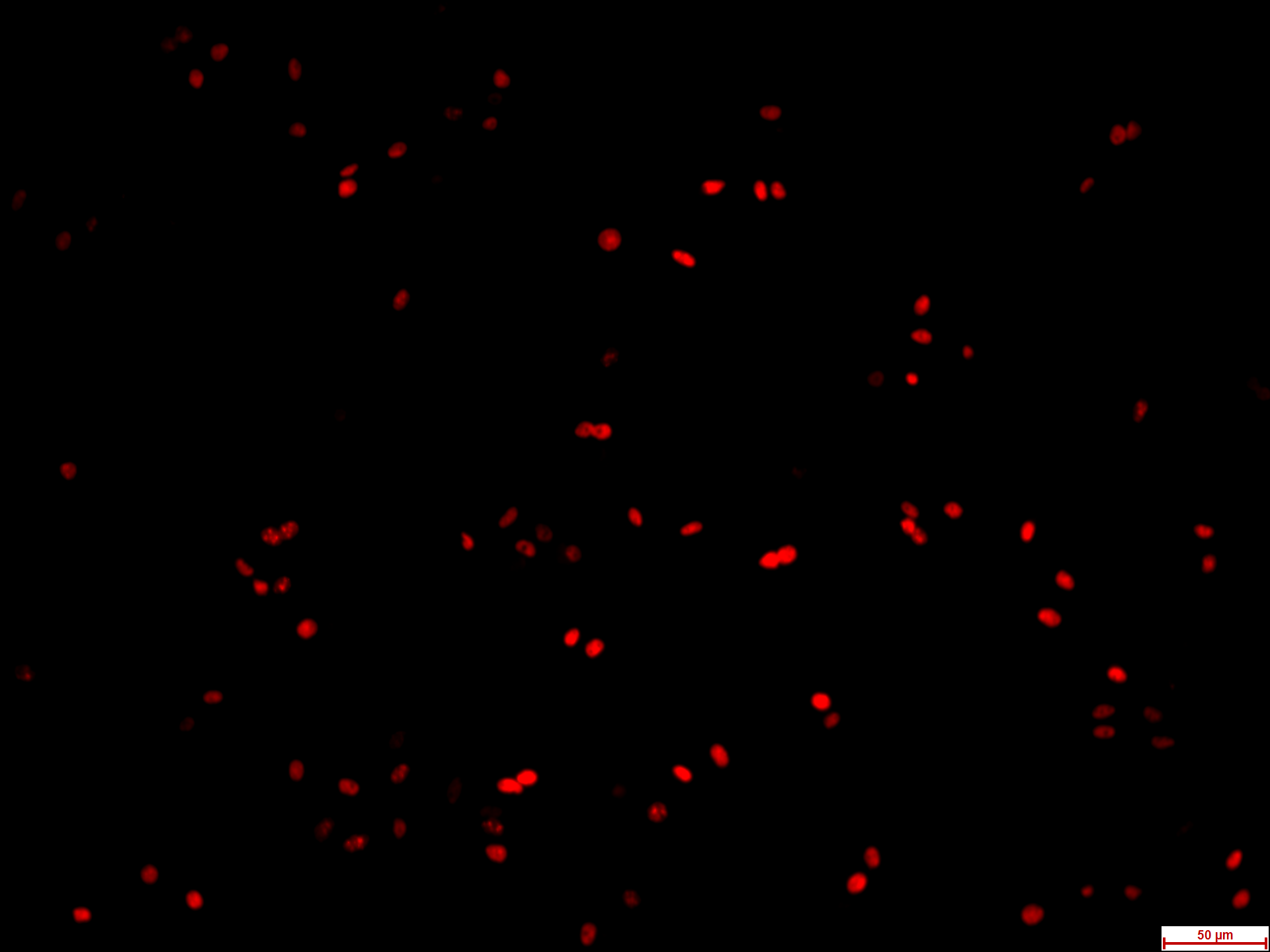

Supplement: Supplementary file 1 [file biomolecules-14-00677-s001.zip › Raw data/HepG/EDU/23.7.10-edu/rpn1/1.tif]

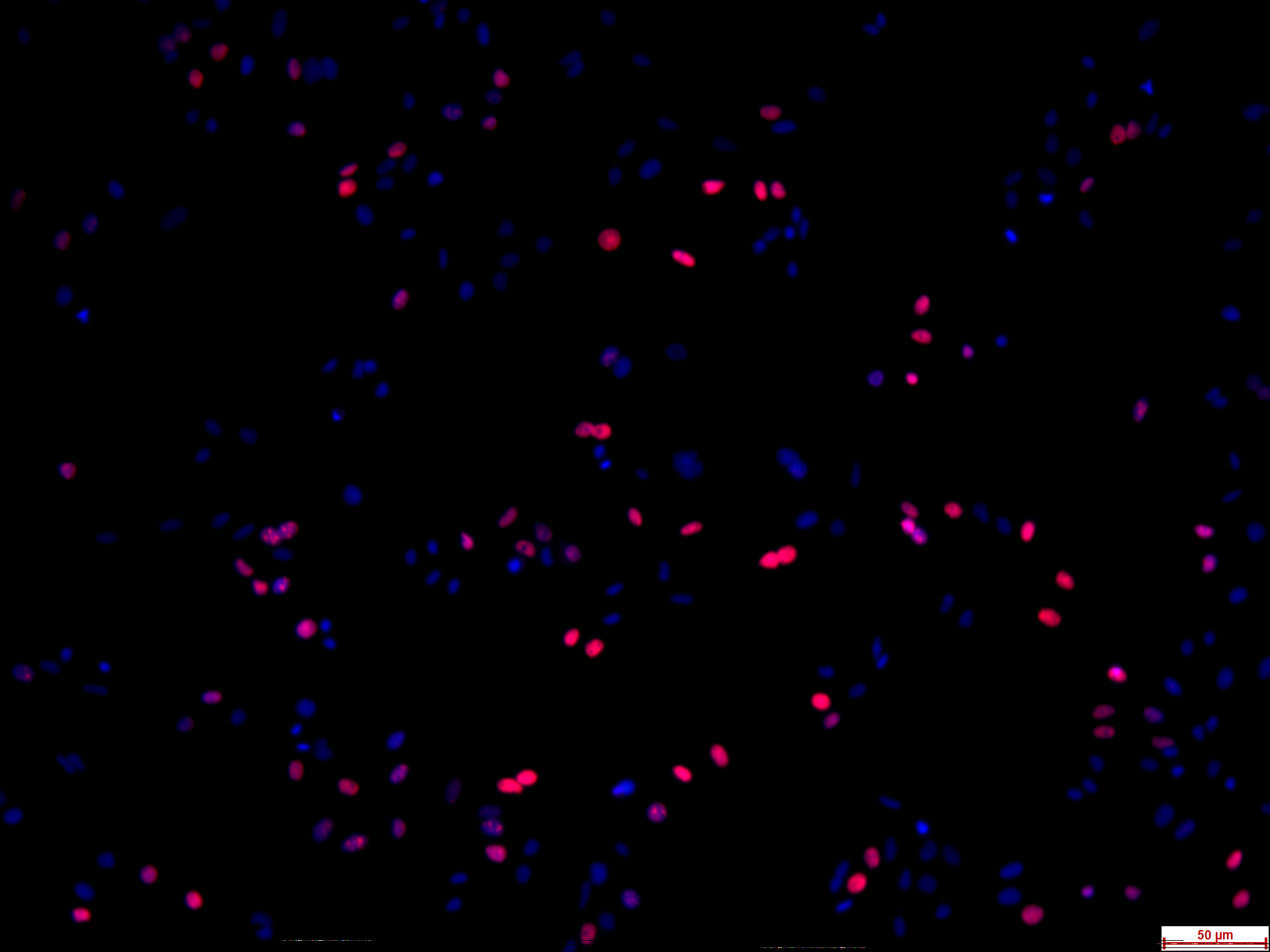

Supplement: Supplementary file 1 [file biomolecules-14-00677-s001.zip › Raw data/HepG/EDU/23.7.10-edu/rpn1/2.tif]

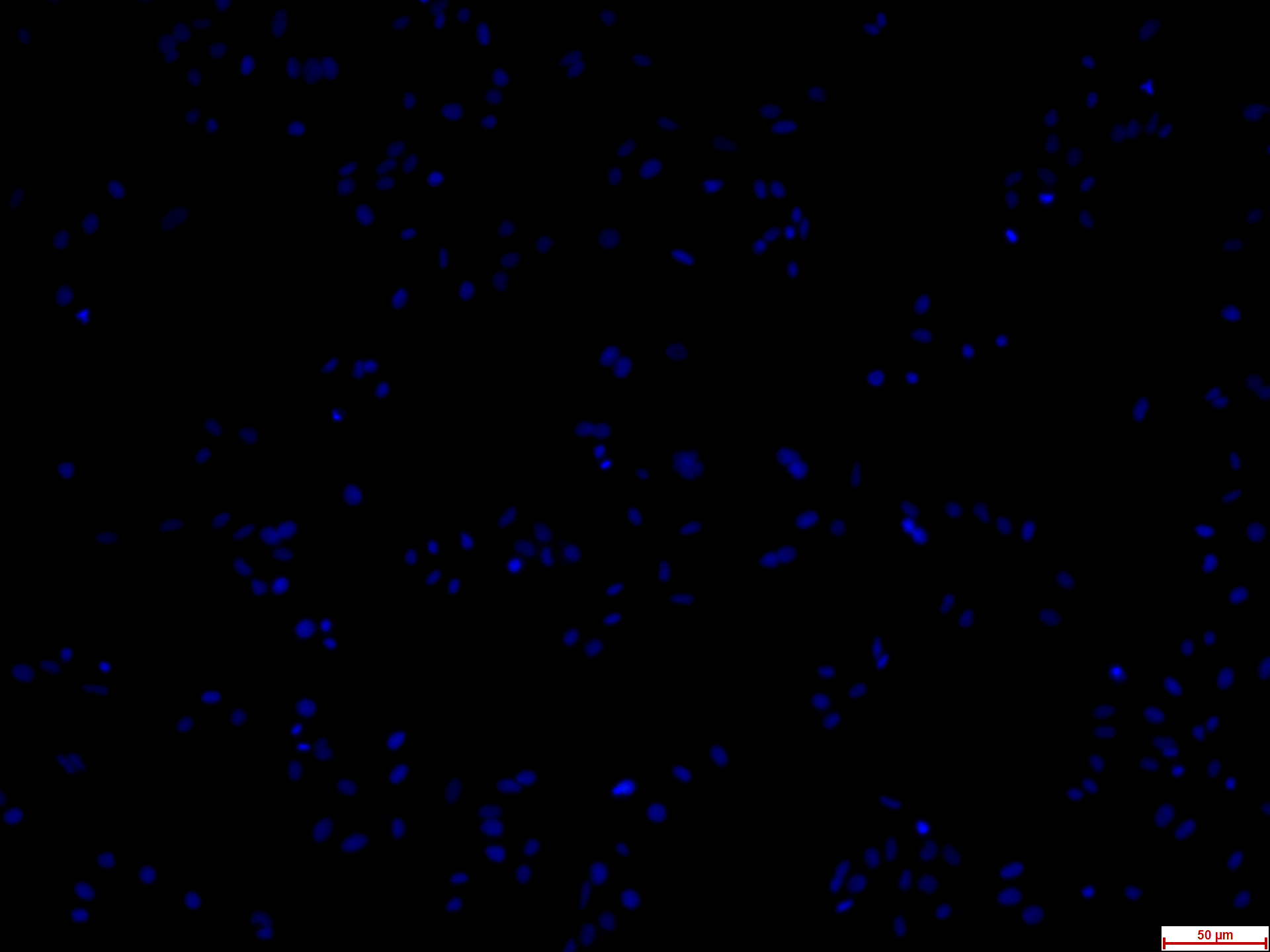

Supplement: Supplementary file 1 [file biomolecules-14-00677-s001.zip › Raw data/HepG/EDU/23.7.10-edu/rpn1/RN-1.tif]

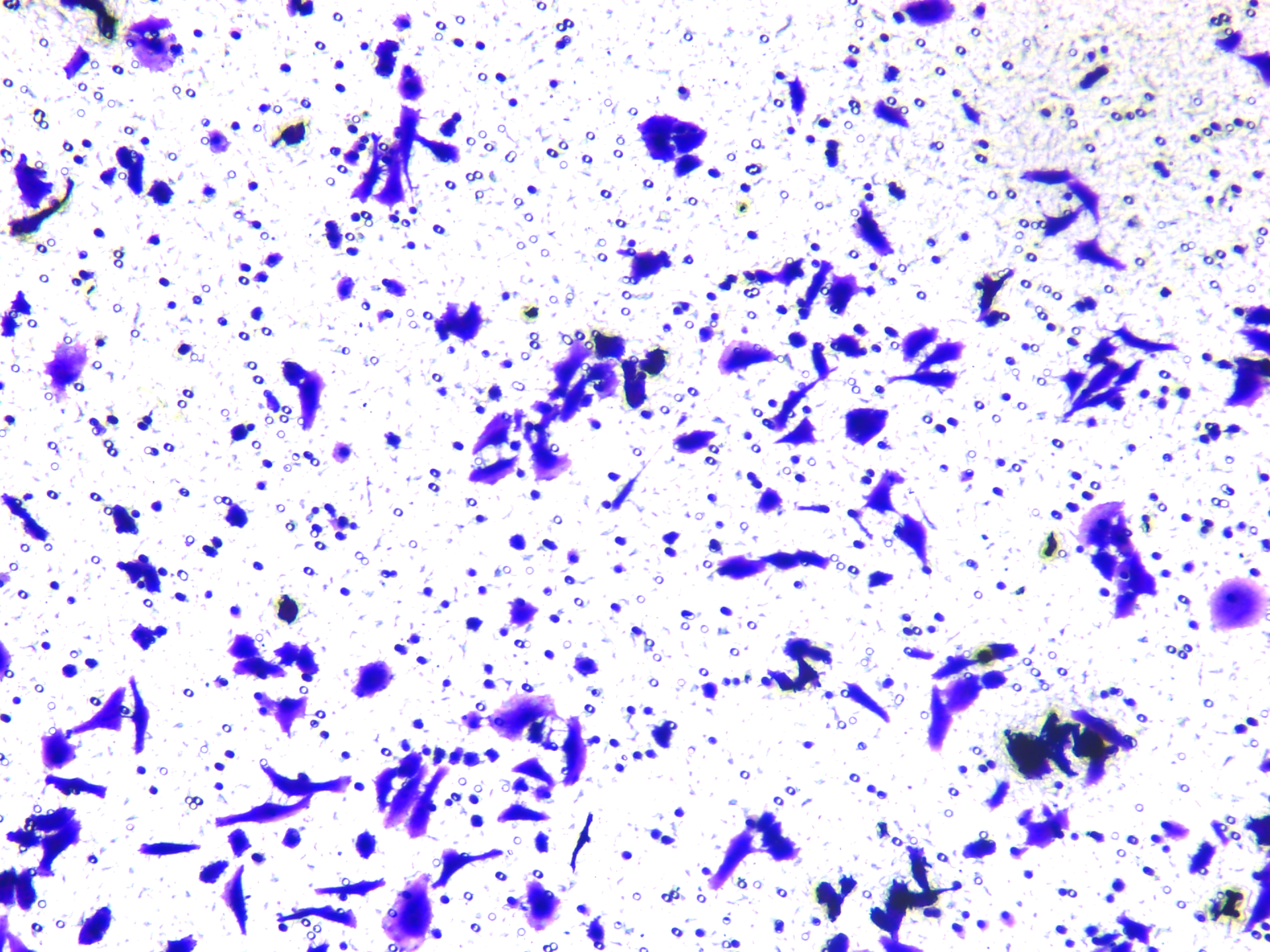

Supplement: Supplementary file 1 [file biomolecules-14-00677-s001.zip › Raw data/HepG/Transwell/G2-PK_RAW_ch00.tif]

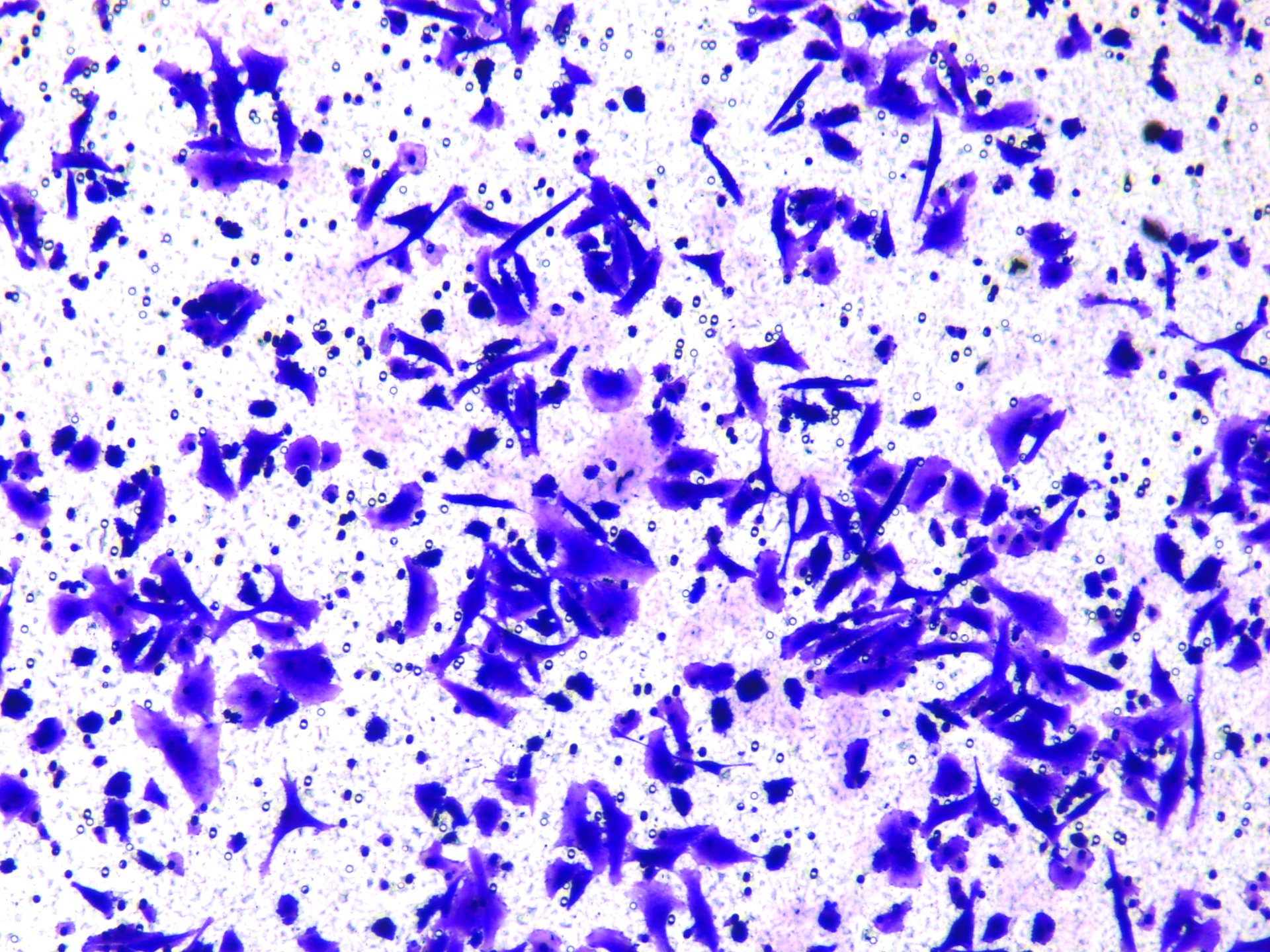

Supplement: Supplementary file 1 [file biomolecules-14-00677-s001.zip › Raw data/HepG/Transwell/G2-RPN1-1_RAW_ch00.tif]

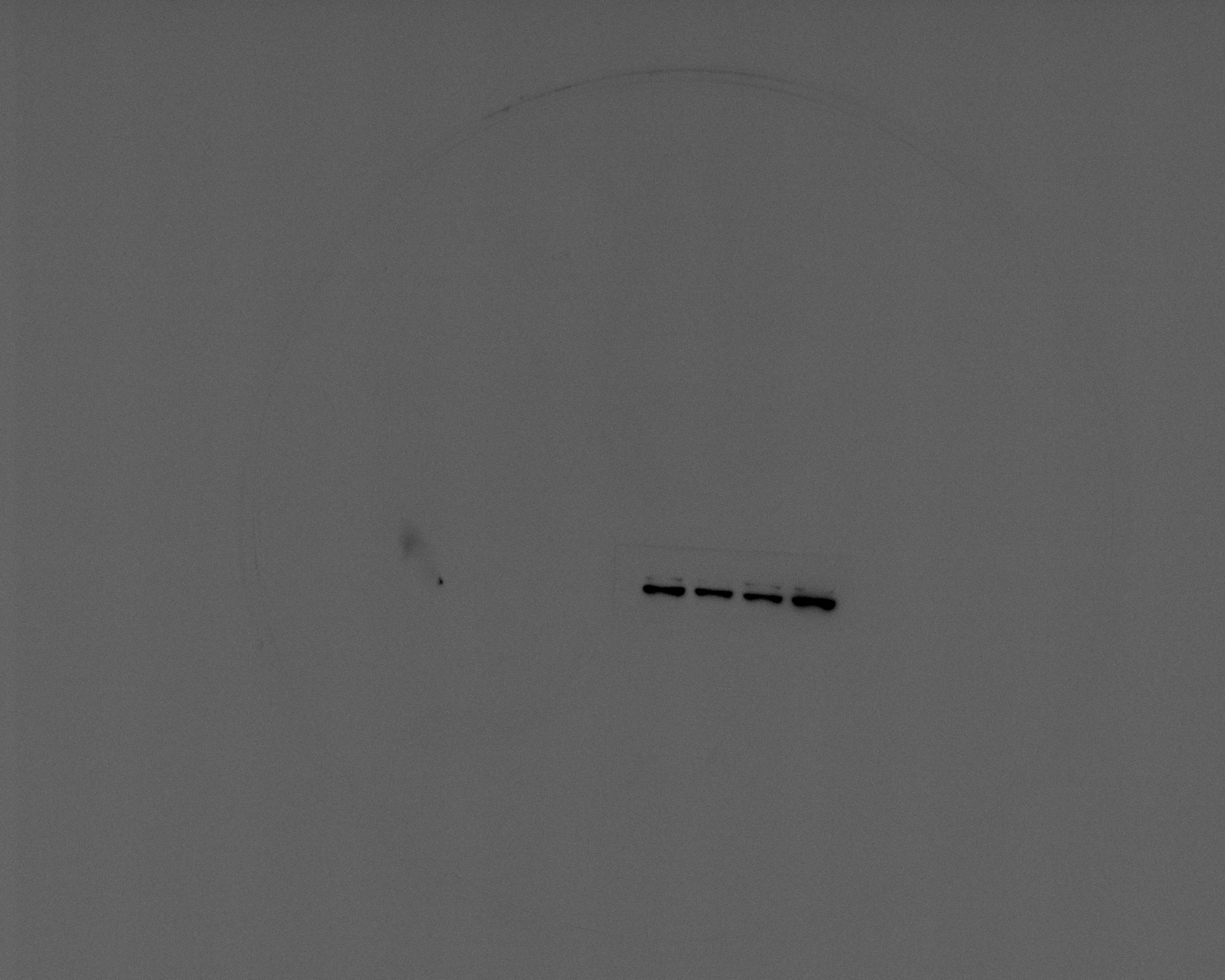

Supplement: Supplementary file 1 [file biomolecules-14-00677-s001.zip › Raw data/HepG/WB/FIGA-1.jpg]

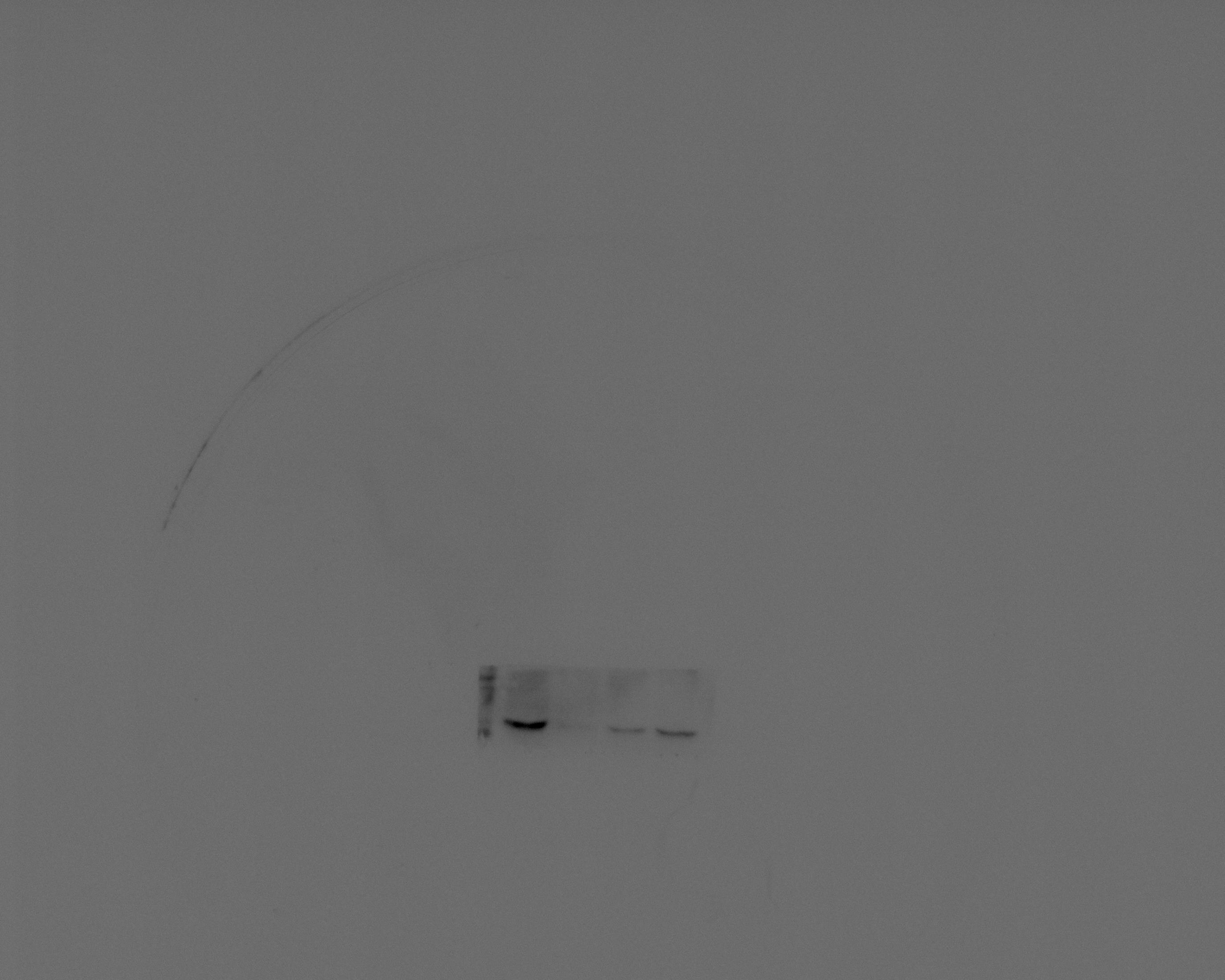

Supplement: Supplementary file 1 [file biomolecules-14-00677-s001.zip › Raw data/HepG/WB/FigA-2.jpg]

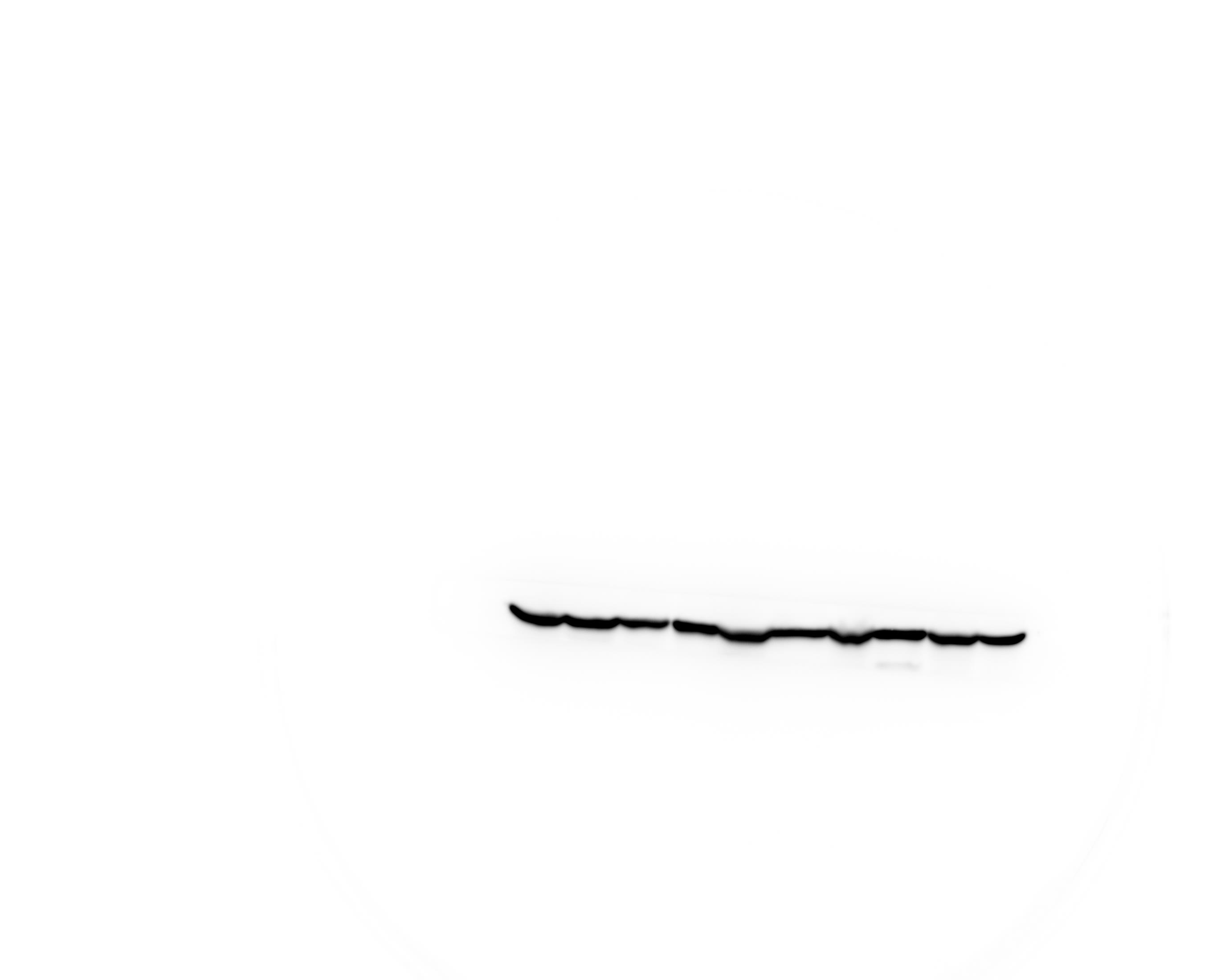

Supplement: Supplementary file 1 [file biomolecules-14-00677-s001.zip › Raw data/HepG/WB/FigB-1.jpg]

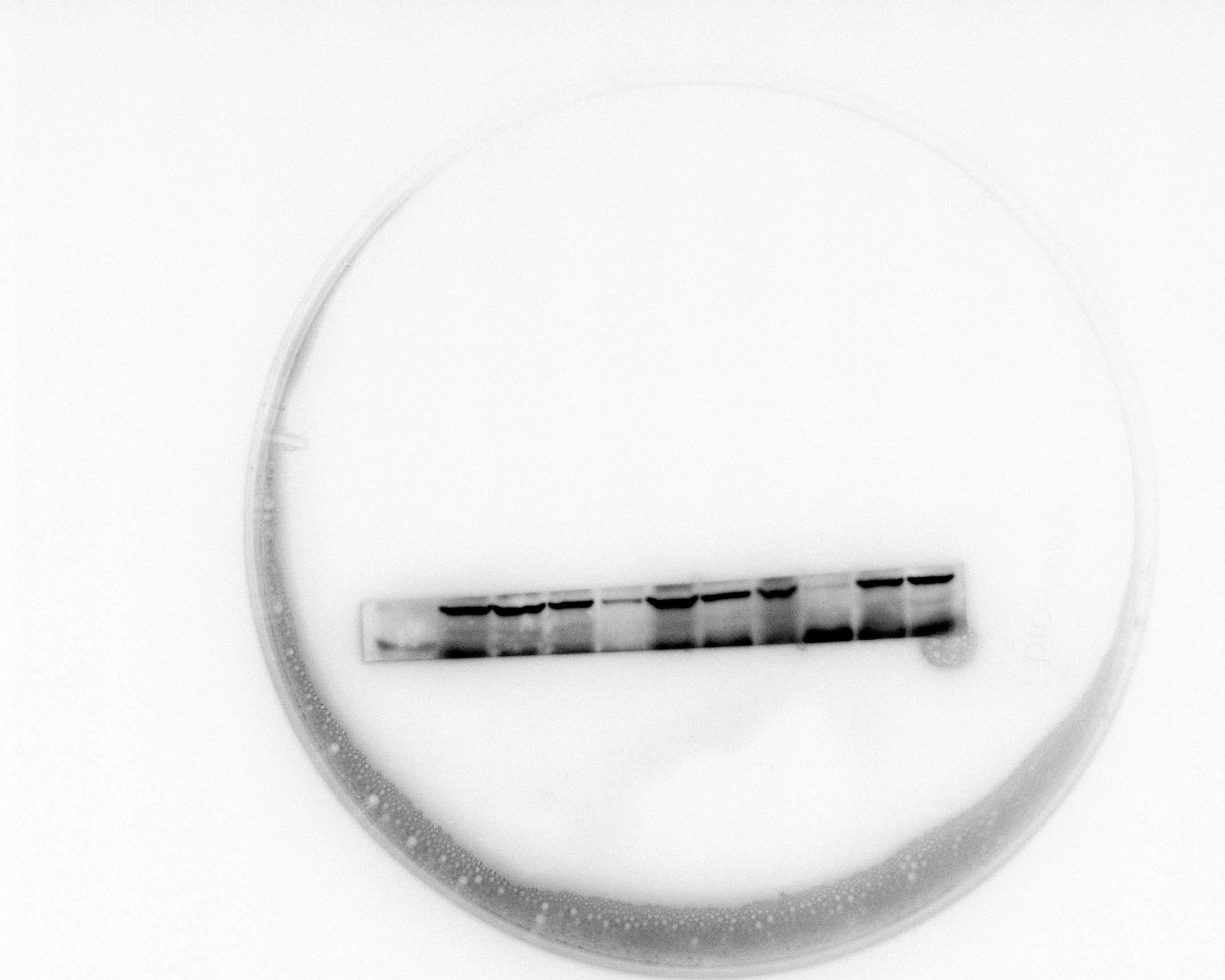

Supplement: Supplementary file 1 [file biomolecules-14-00677-s001.zip › Raw data/HepG/WB/FigB-2.jpg]

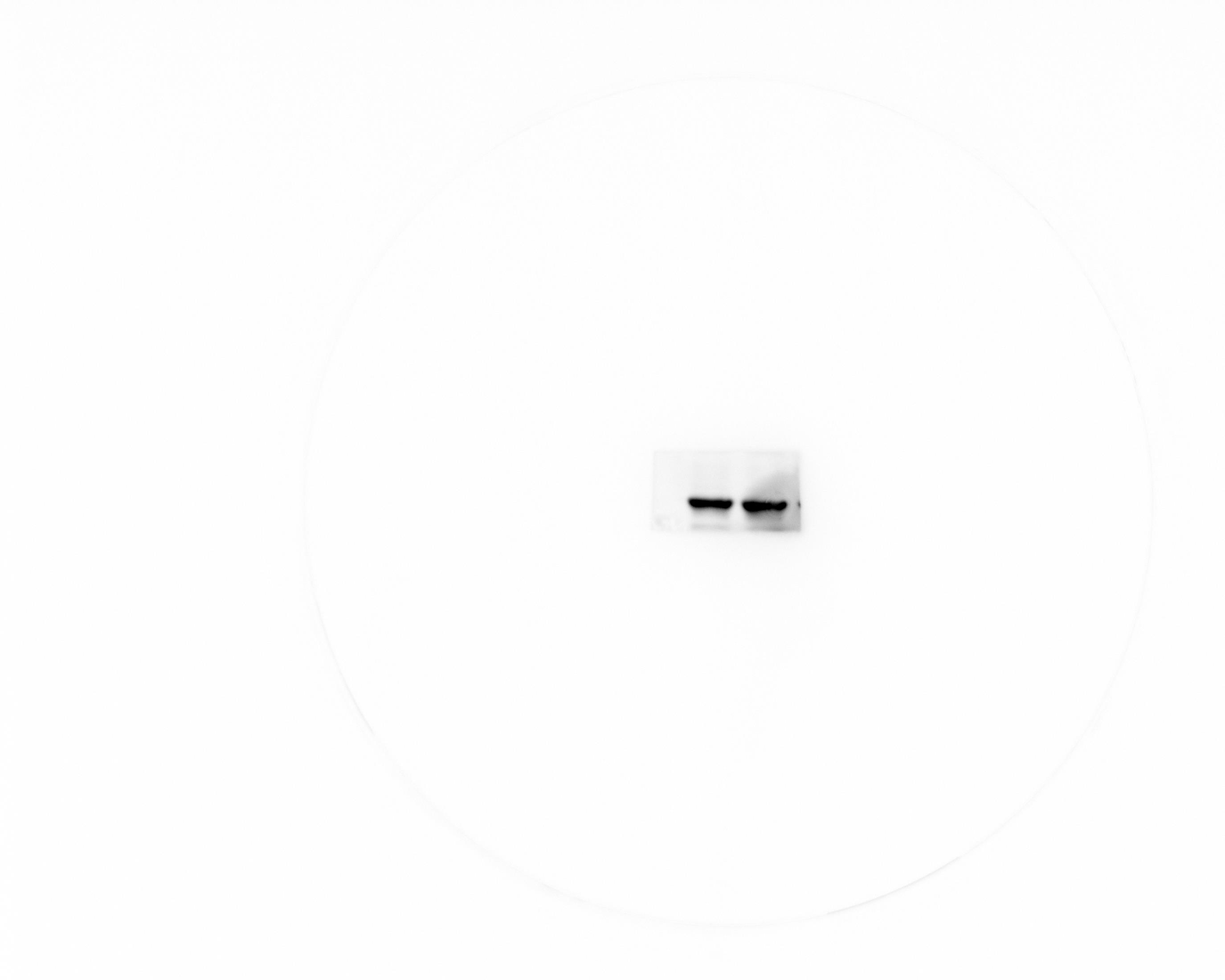

Supplement: Supplementary file 1 [file biomolecules-14-00677-s001.zip › Raw data/HepG/WB/FigD-1.jpg]

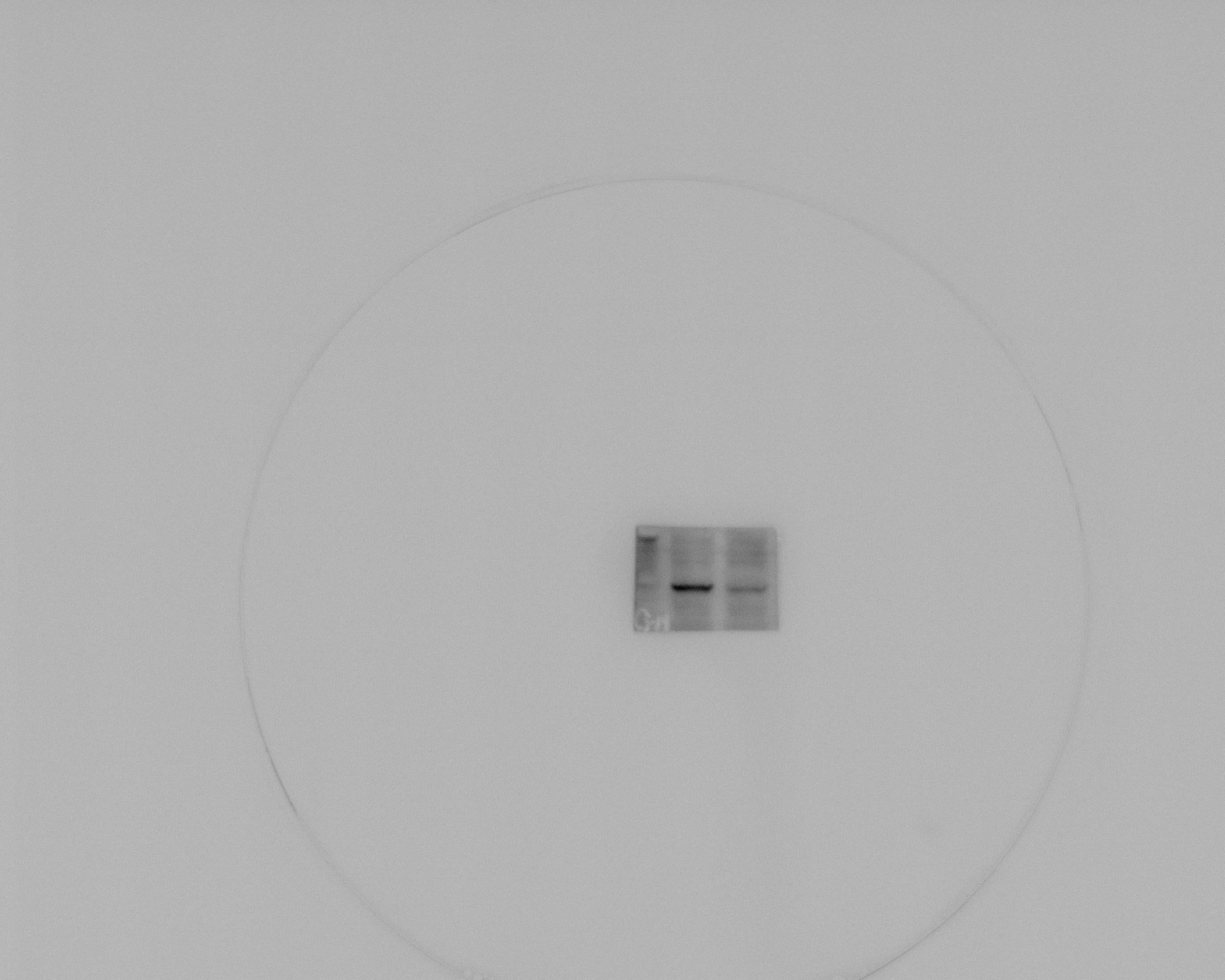

Supplement: Supplementary file 1 [file biomolecules-14-00677-s001.zip › Raw data/HepG/WB/FigD-2.jpg]

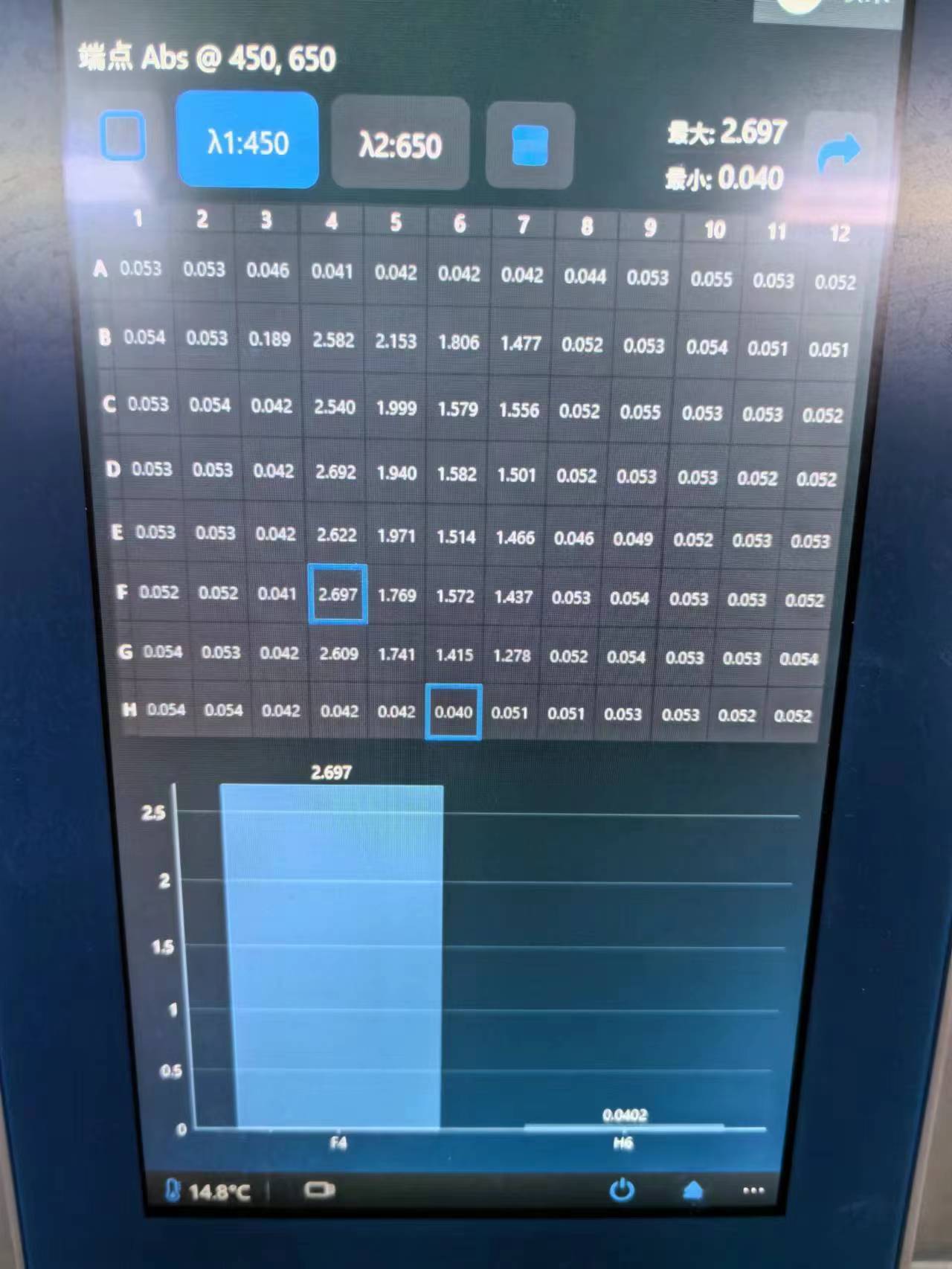

Supplement: Supplementary file 1 [file biomolecules-14-00677-s001.zip › Raw data/Huh 7/cck8-rpn1-Huh7/_20240110100129.jpg]

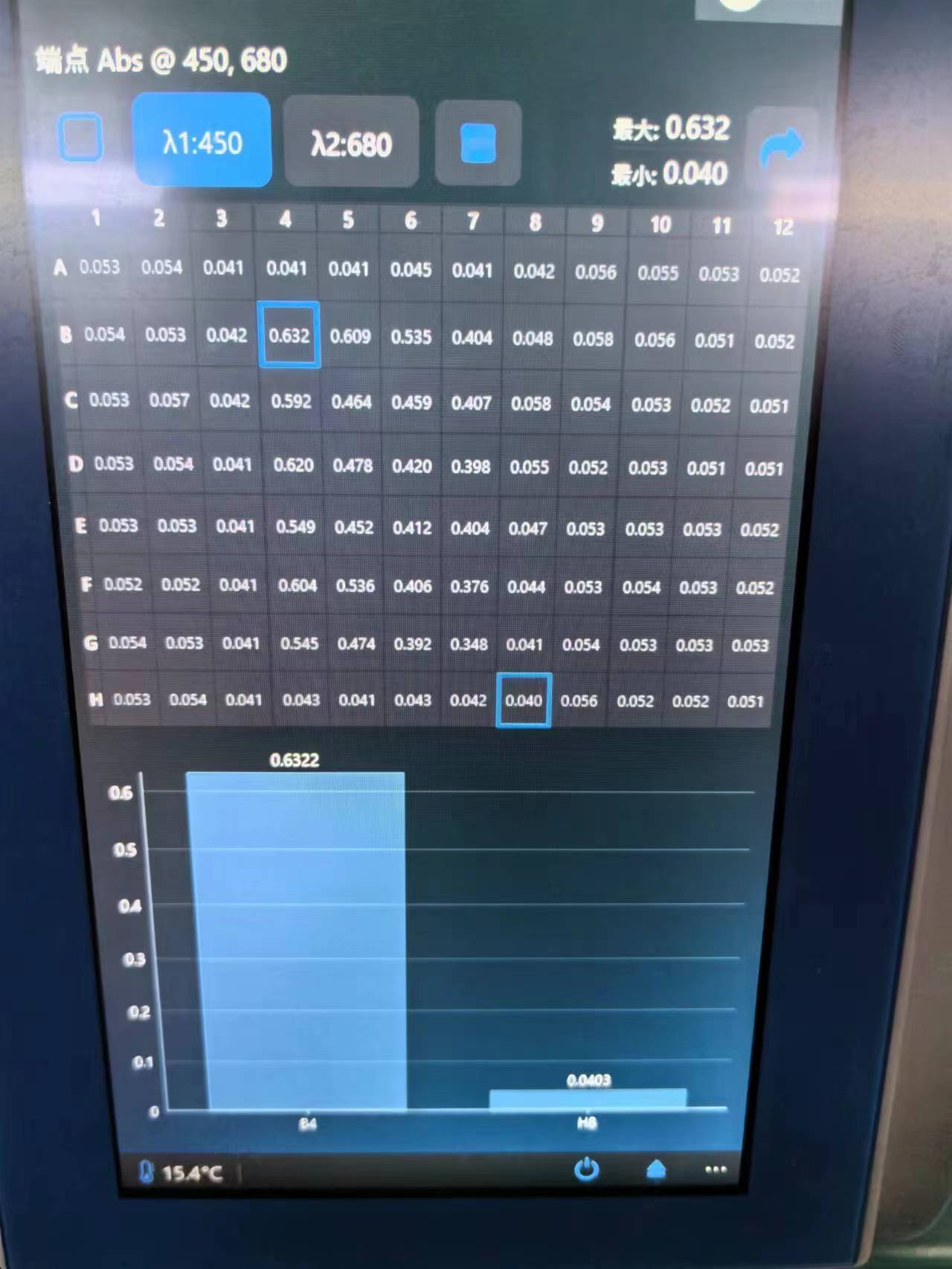

Supplement: Supplementary file 1 [file biomolecules-14-00677-s001.zip › Raw data/Huh 7/cck8-rpn1-Huh7/_20240110100142.jpg]

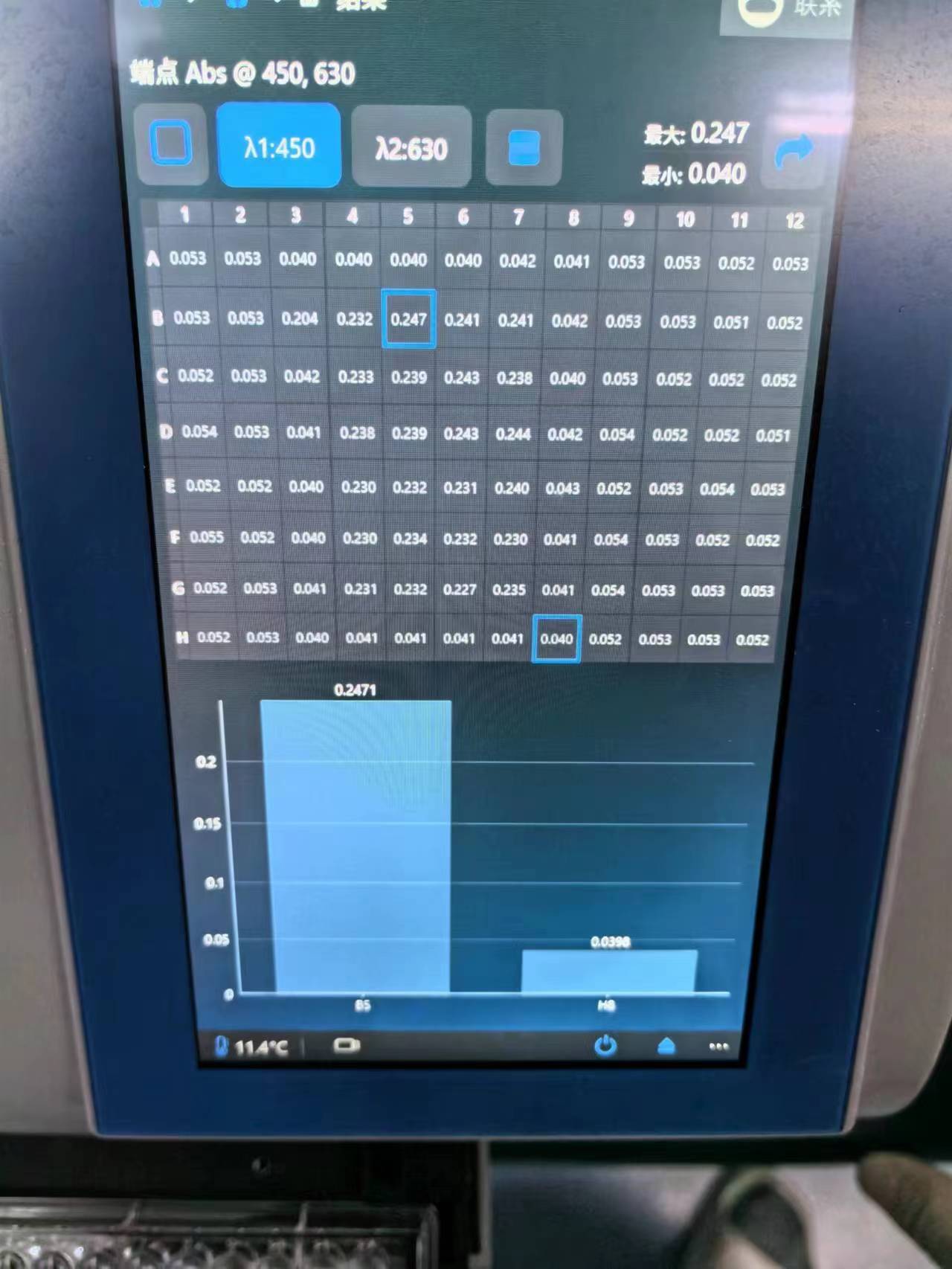

Supplement: Supplementary file 1 [file biomolecules-14-00677-s001.zip › Raw data/Huh 7/cck8-rpn1-Huh7/_20240110100149.jpg]

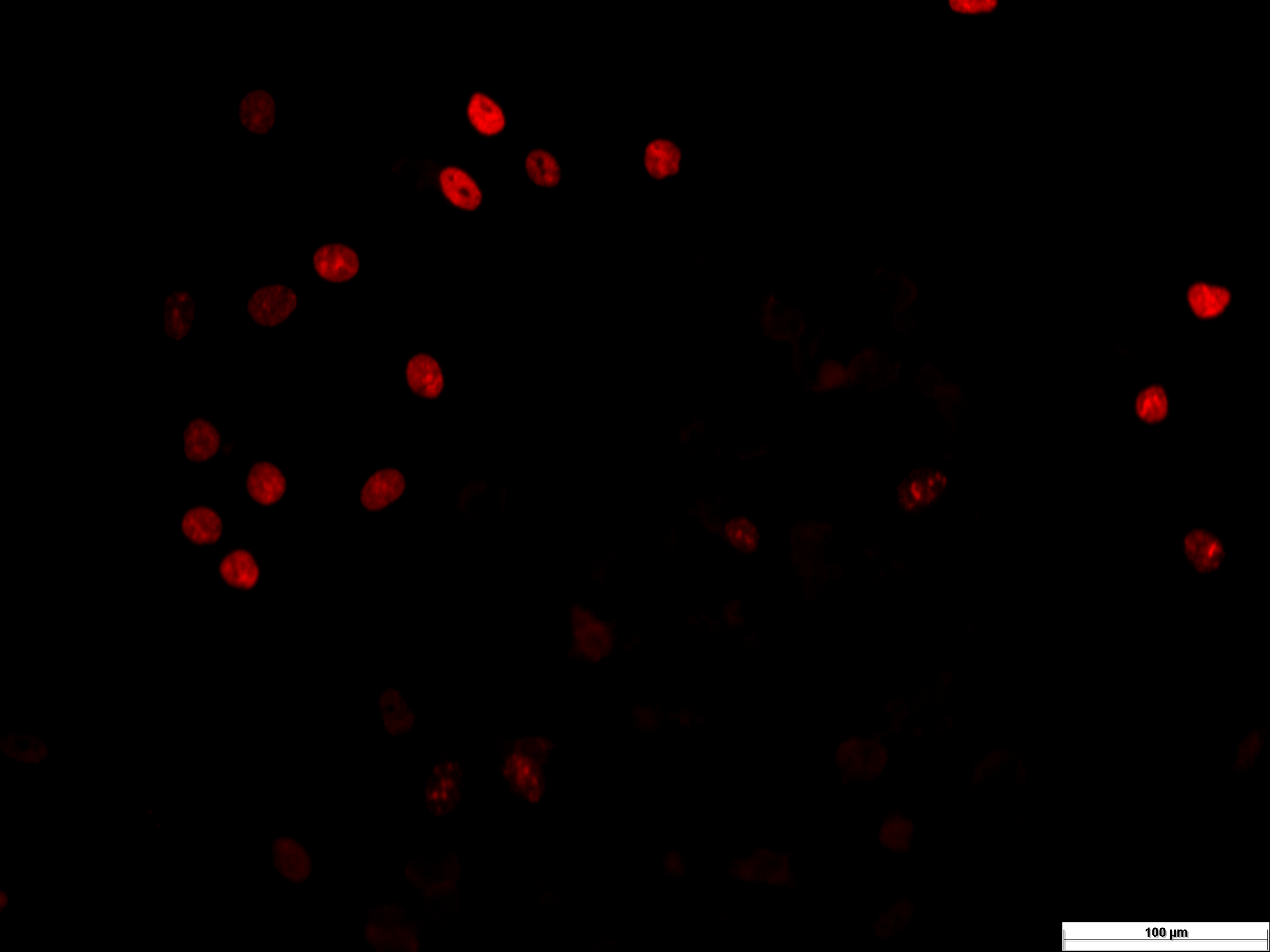

Supplement: Supplementary file 1 [file biomolecules-14-00677-s001.zip › Raw data/Huh 7/EDU/1/2.tif]

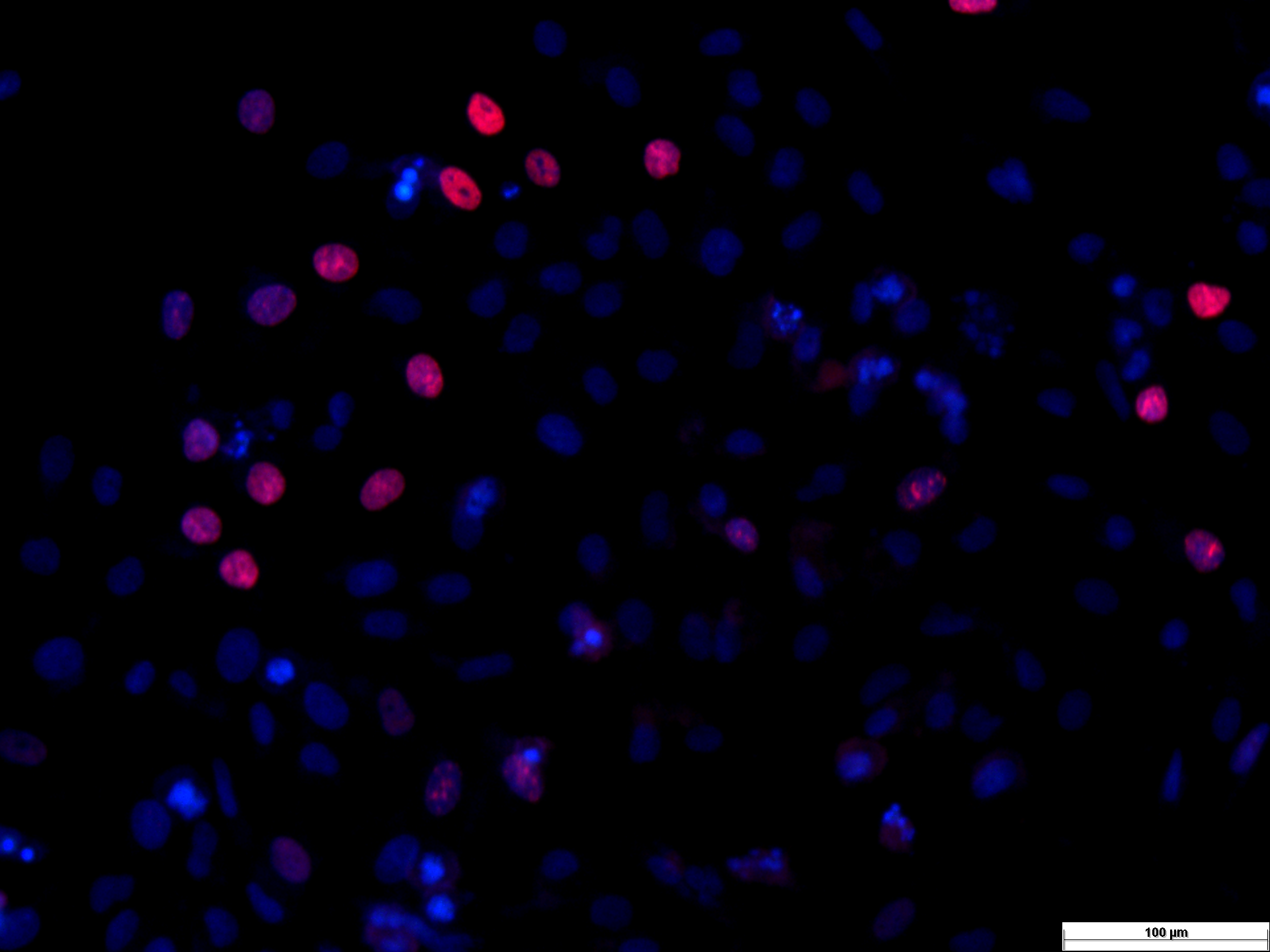

Supplement: Supplementary file 1 [file biomolecules-14-00677-s001.zip › Raw data/Huh 7/EDU/1/3.tif]

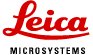

Supplement: Supplementary file 1 [file biomolecules-14-00677-s001.zip › Raw data/Huh 7/EDU/1/MetaData/LeicaLogo.jpg]

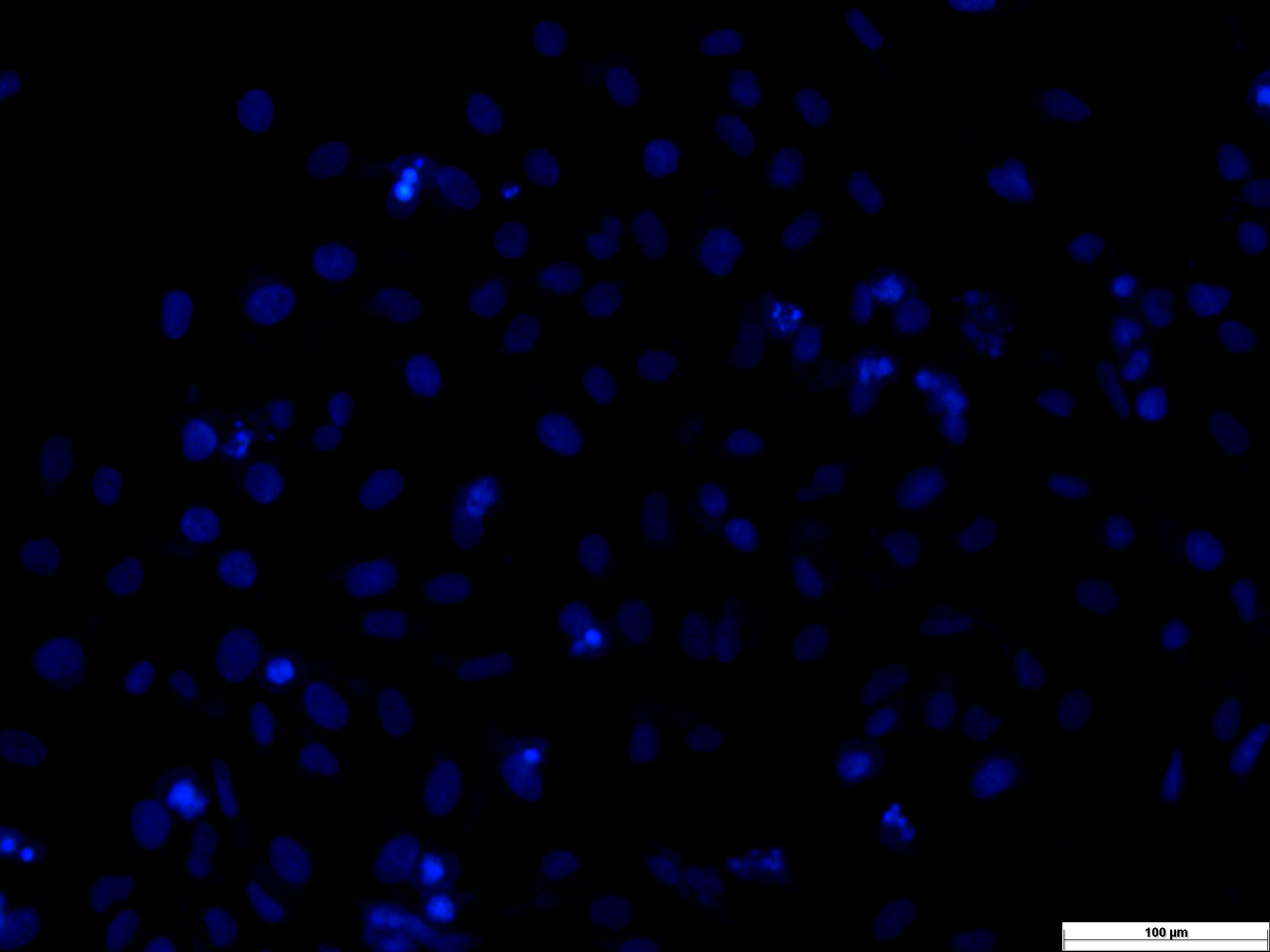

Supplement: Supplementary file 1 [file biomolecules-14-00677-s001.zip › Raw data/Huh 7/EDU/1/Overlay003.tif]

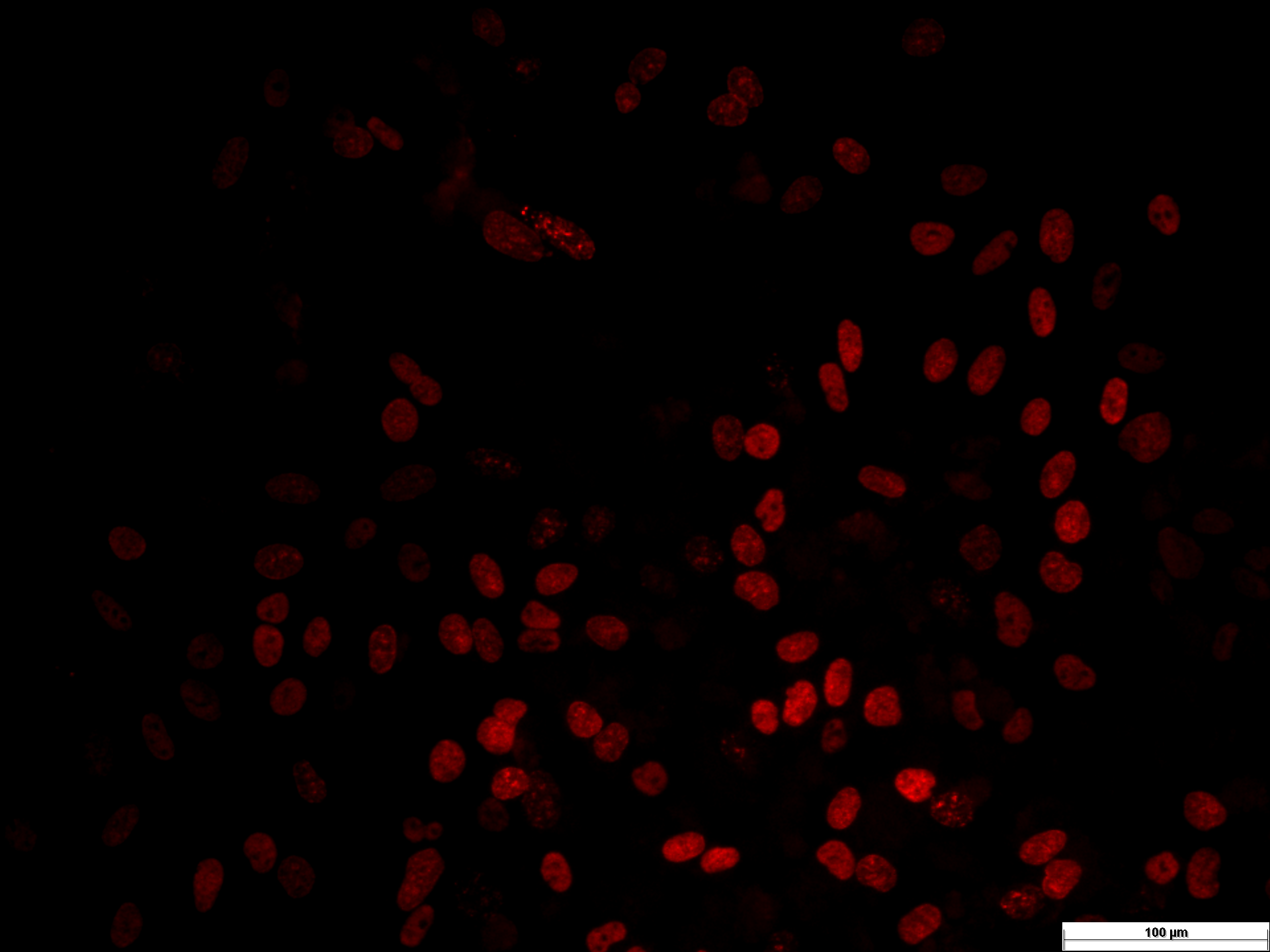

Supplement: Supplementary file 1 [file biomolecules-14-00677-s001.zip › Raw data/Huh 7/EDU/2/1.tif]

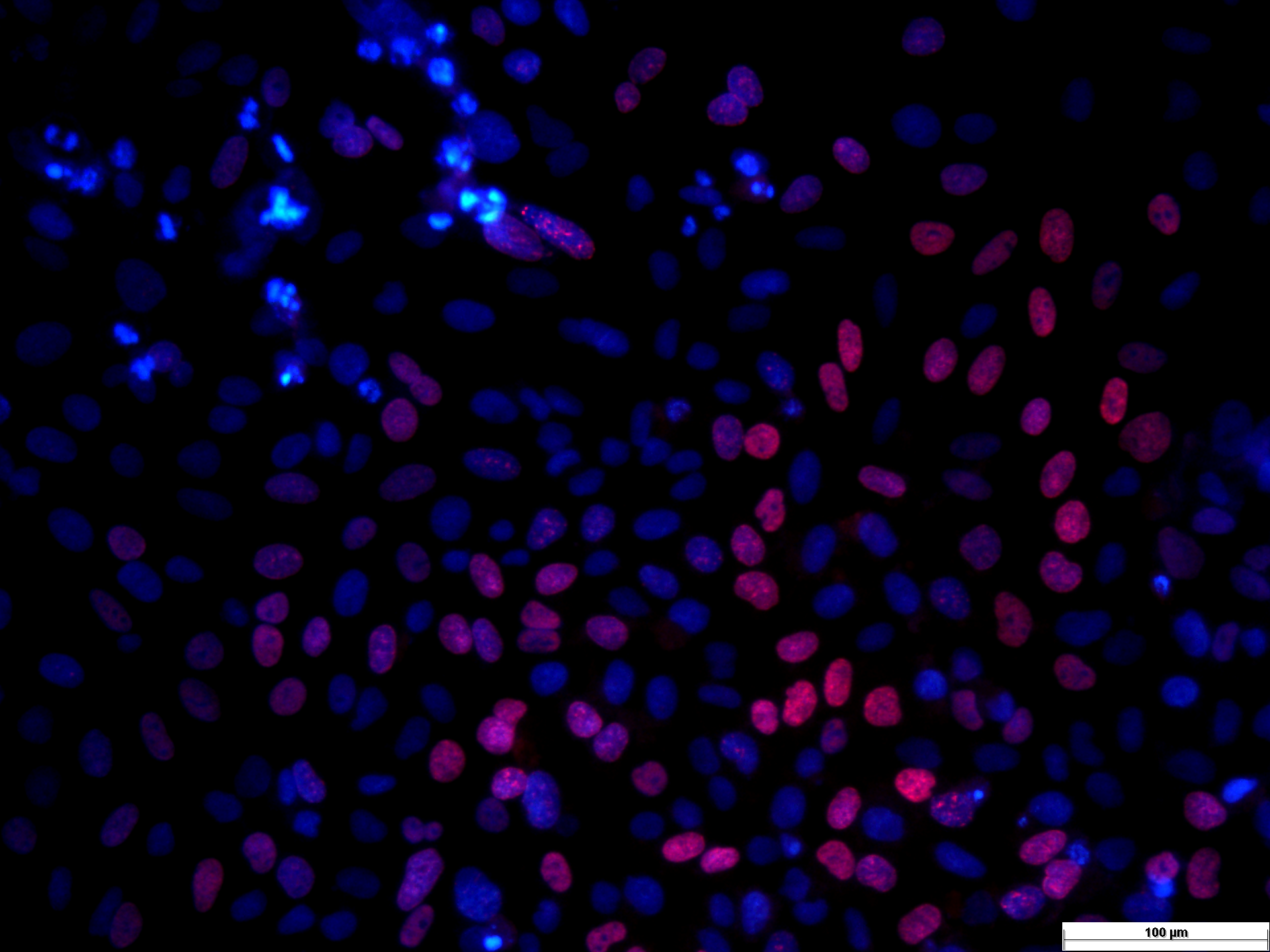

Supplement: Supplementary file 1 [file biomolecules-14-00677-s001.zip › Raw data/Huh 7/EDU/2/2.tif]

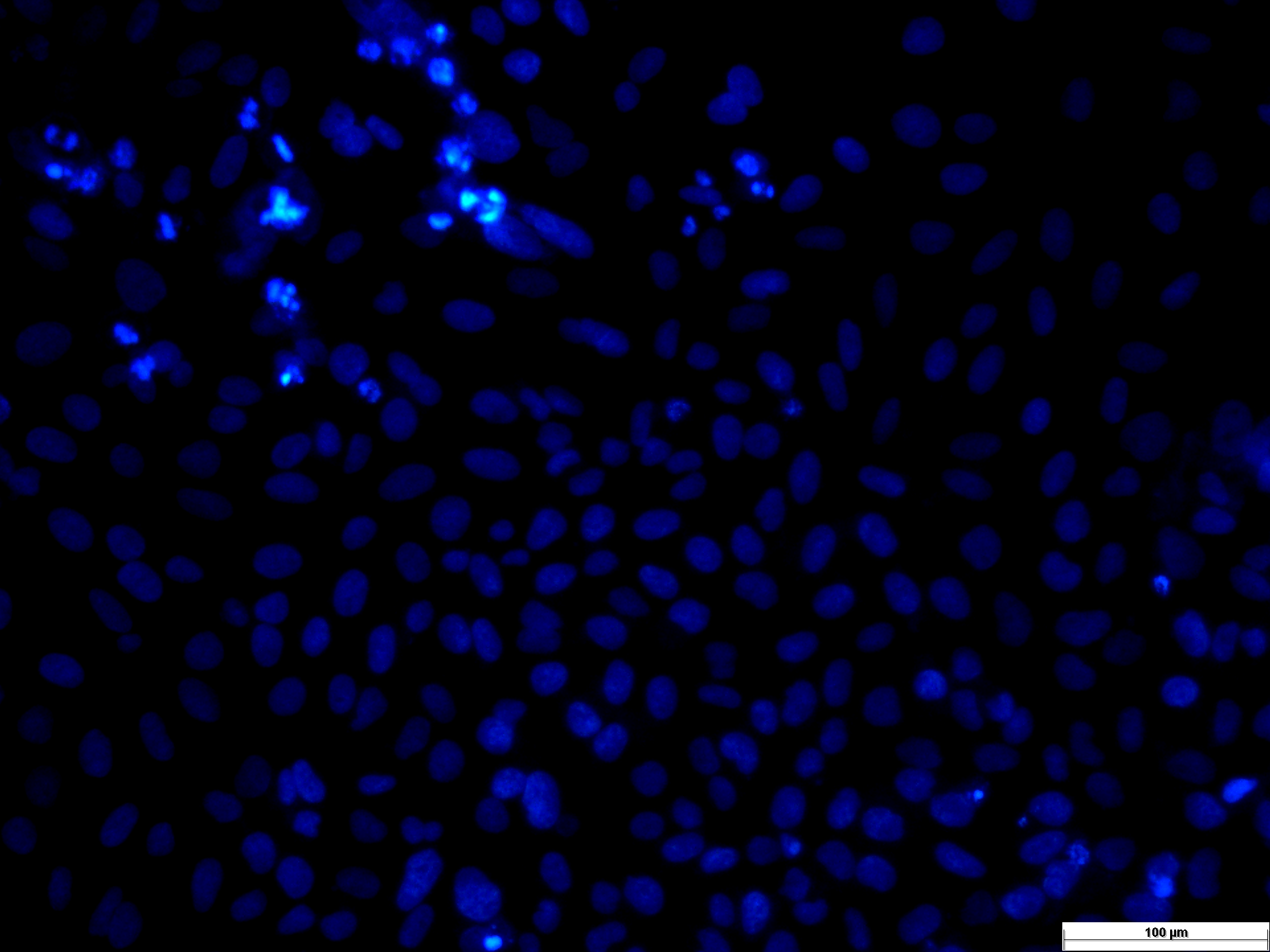

Supplement: Supplementary file 1 [file biomolecules-14-00677-s001.zip › Raw data/Huh 7/EDU/2/Overlay004.tif]

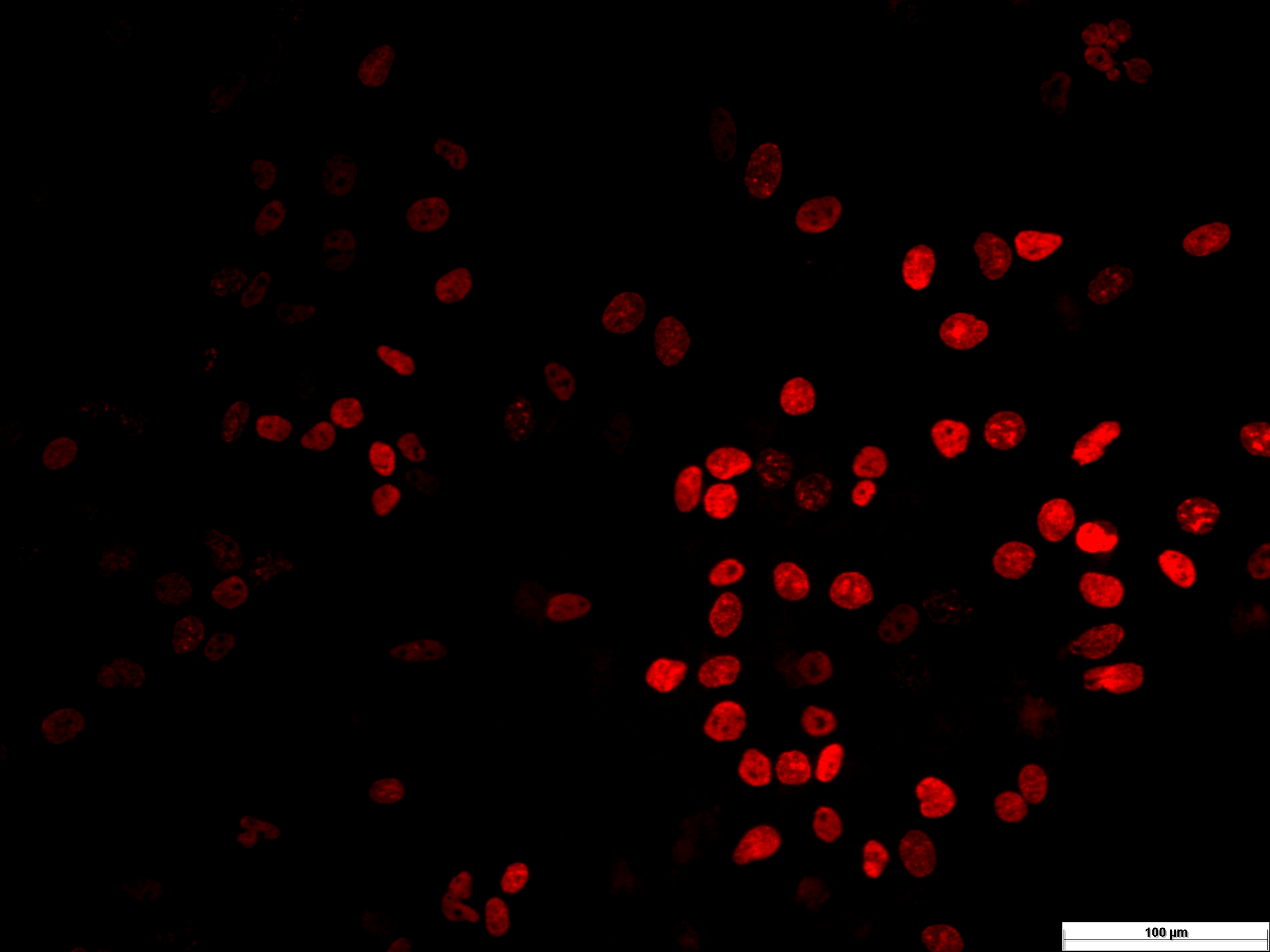

Supplement: Supplementary file 1 [file biomolecules-14-00677-s001.zip › Raw data/Huh 7/EDU/3/1.tif]

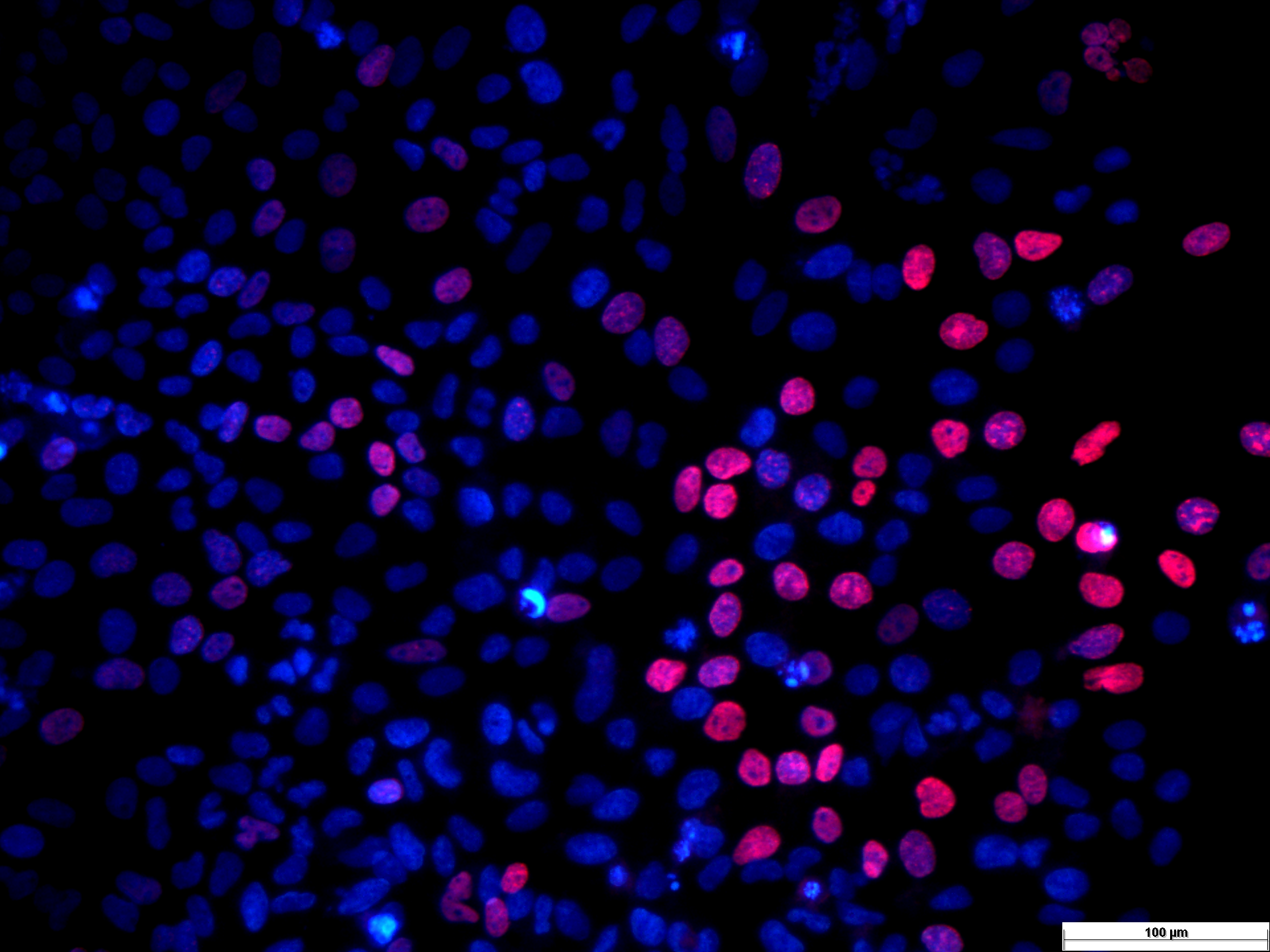

Supplement: Supplementary file 1 [file biomolecules-14-00677-s001.zip › Raw data/Huh 7/EDU/3/2.tif]

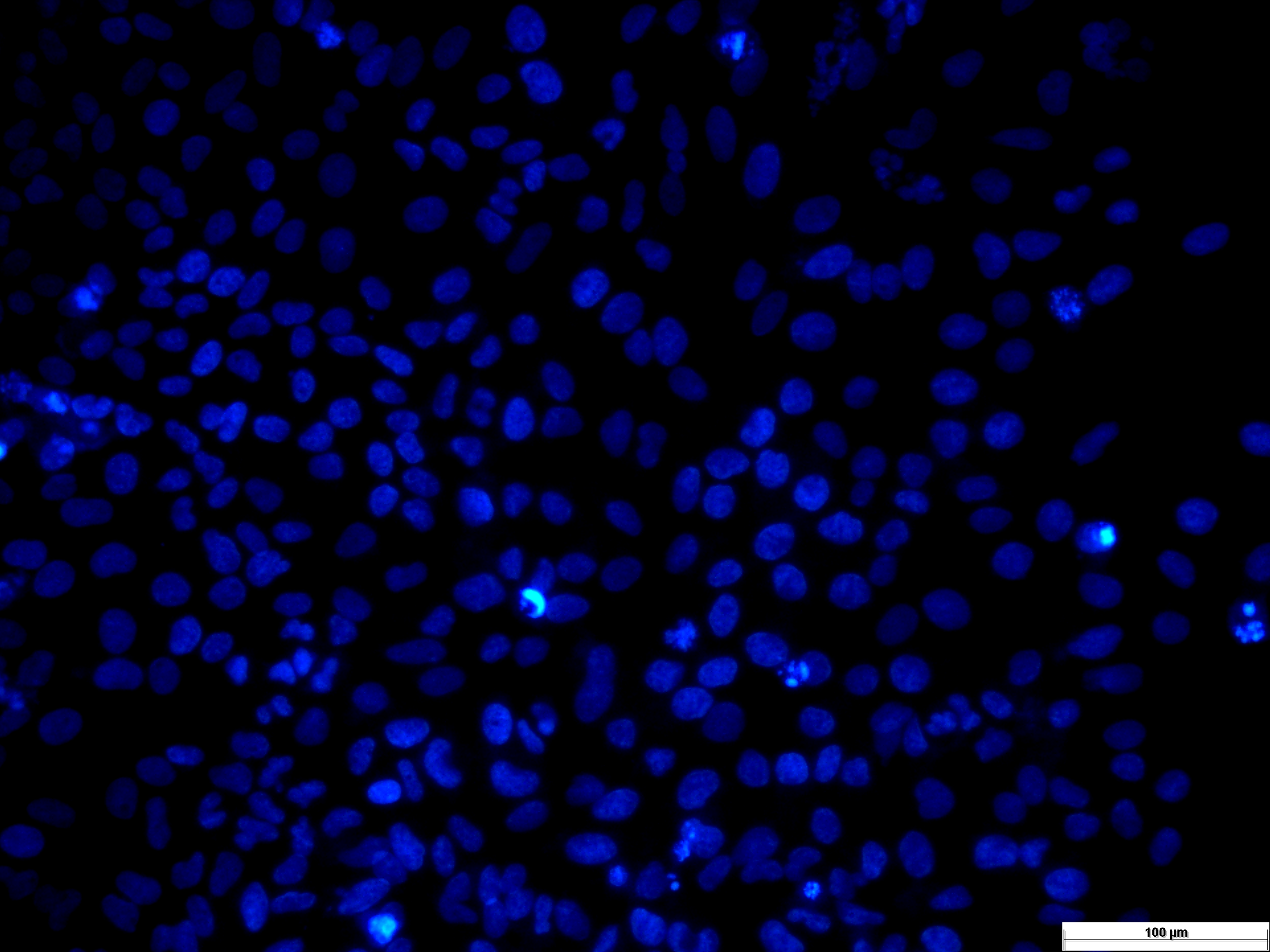

Supplement: Supplementary file 1 [file biomolecules-14-00677-s001.zip › Raw data/Huh 7/EDU/3/Overlay005.tif]

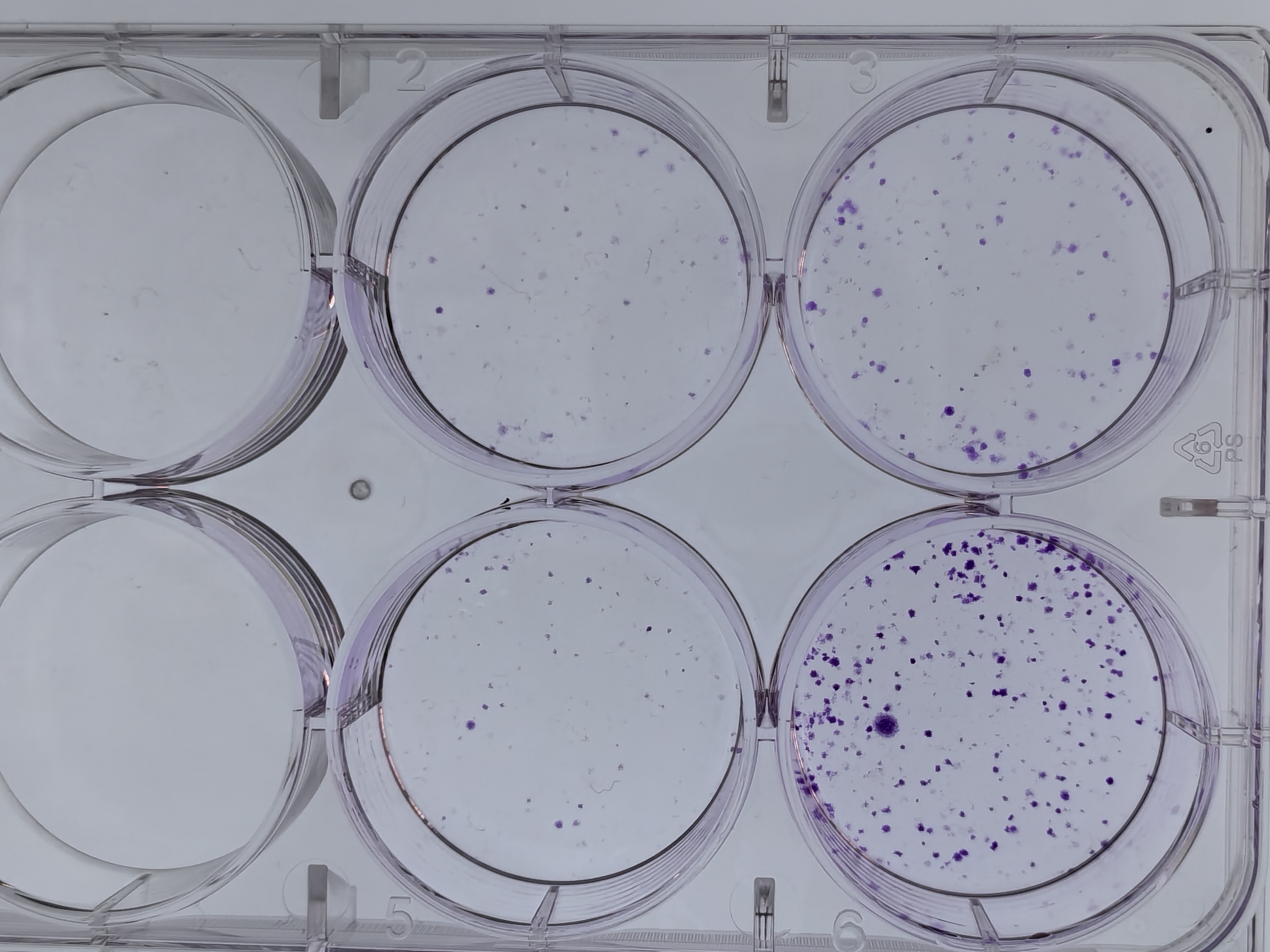

Supplement: Supplementary file 1 [file biomolecules-14-00677-s001.zip › Raw data/Huh 7/RPN1-Huh7-clone/_20240110101250.jpg]

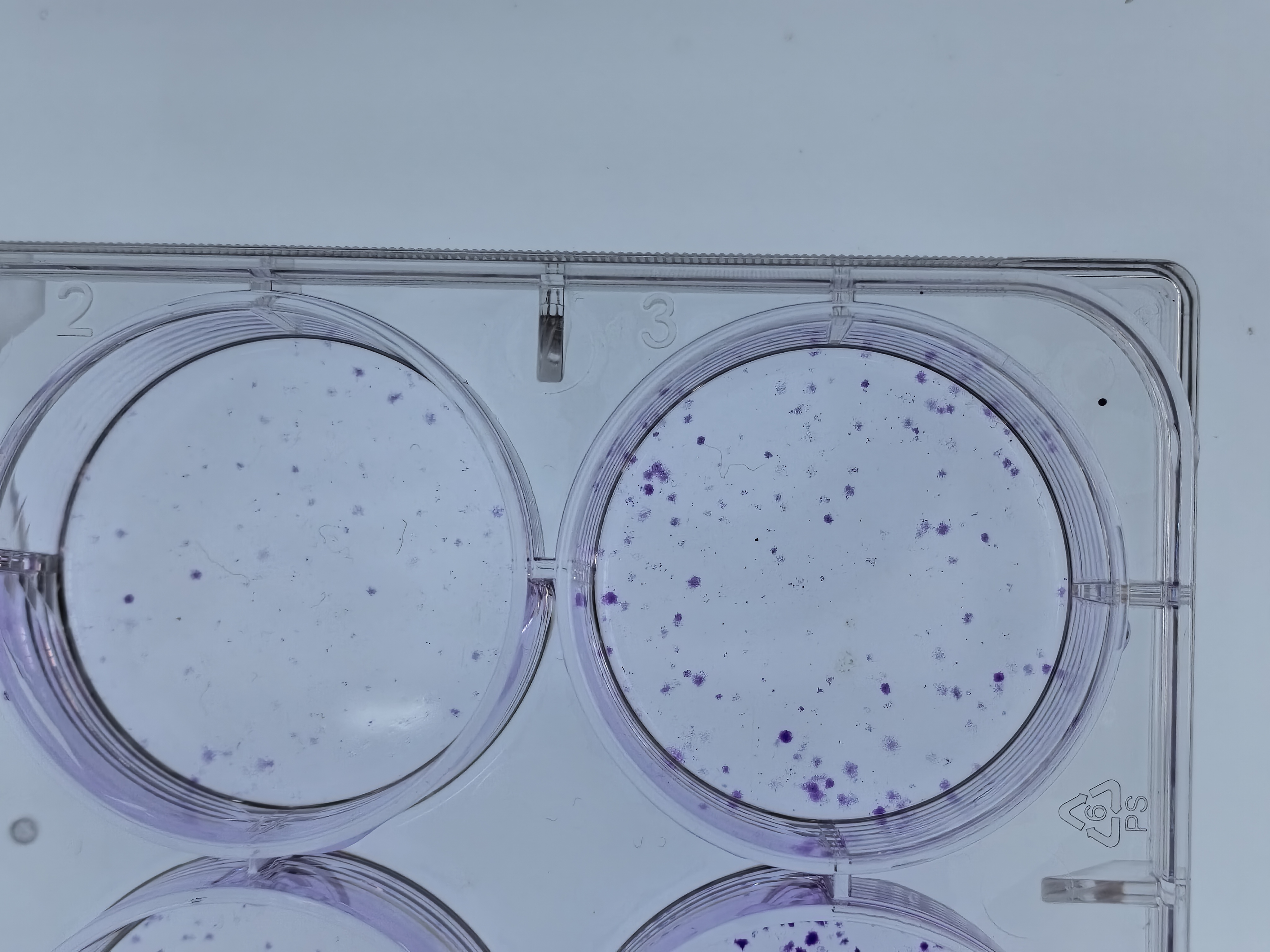

Supplement: Supplementary file 1 [file biomolecules-14-00677-s001.zip › Raw data/Huh 7/RPN1-Huh7-clone/_20240110101301.jpg]

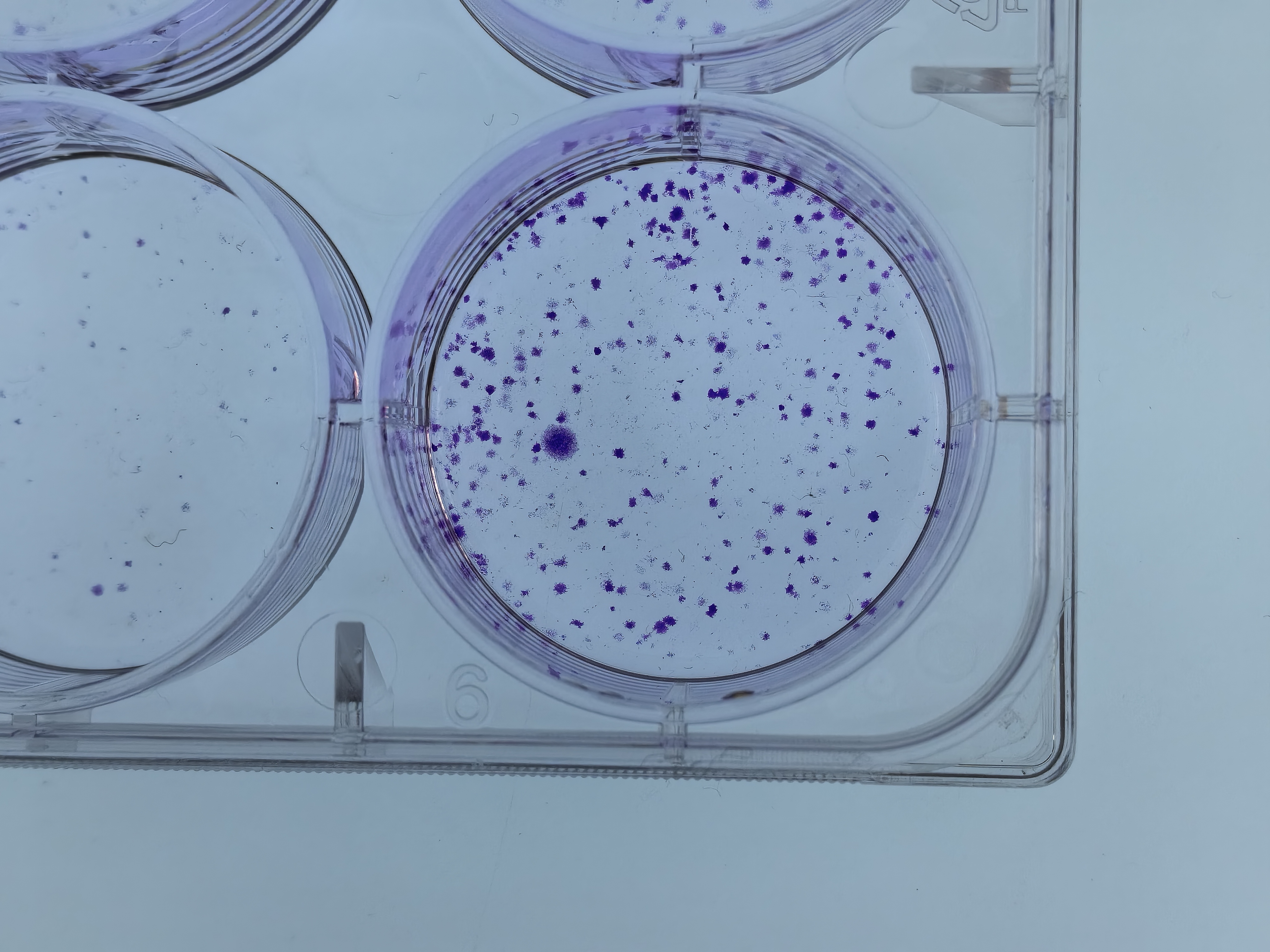

Supplement: Supplementary file 1 [file biomolecules-14-00677-s001.zip › Raw data/Huh 7/RPN1-Huh7-clone/_20240110101310.jpg]

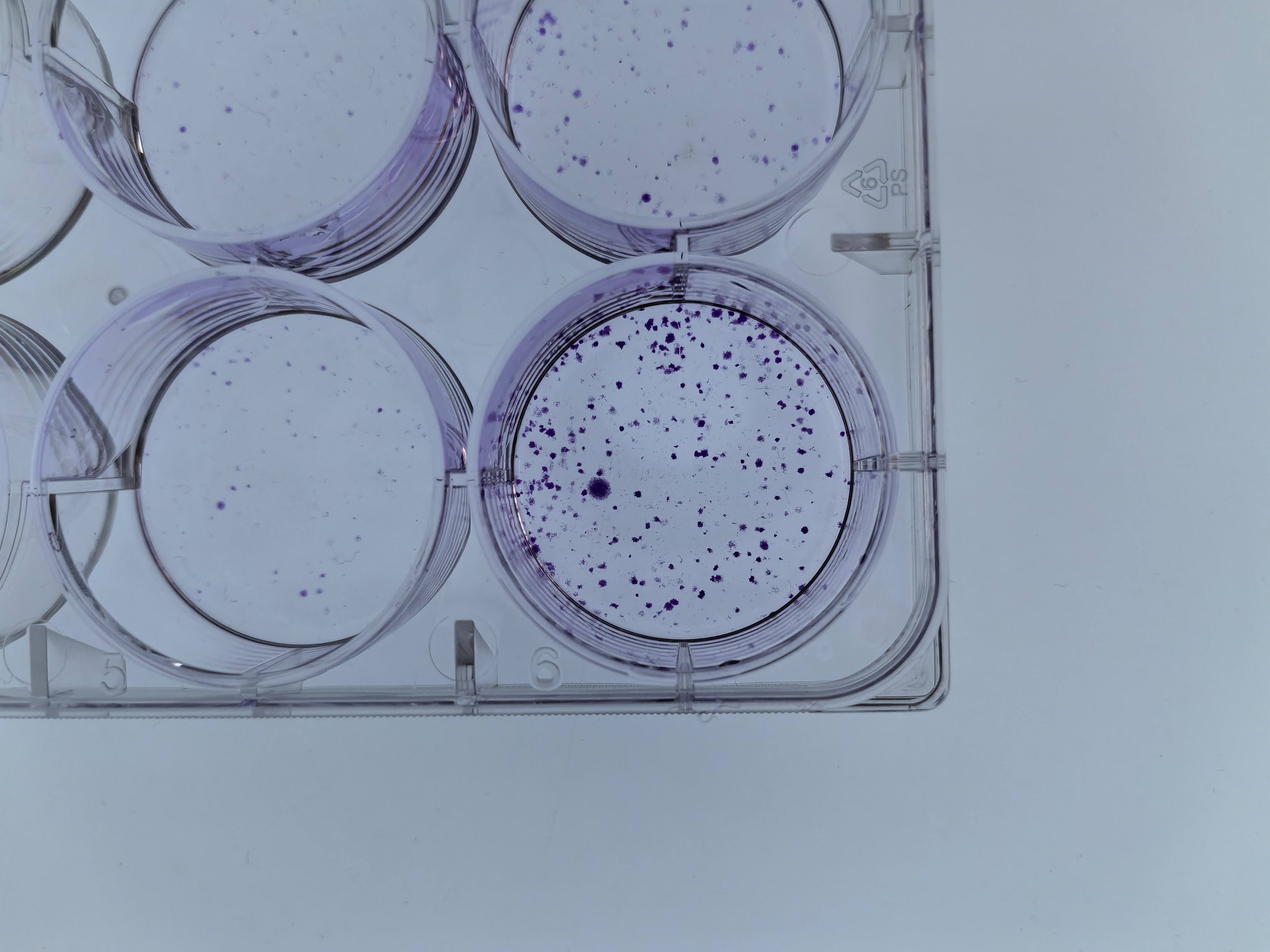

Supplement: Supplementary file 1 [file biomolecules-14-00677-s001.zip › Raw data/Huh 7/RPN1-Huh7-clone/_20240110101318.jpg]

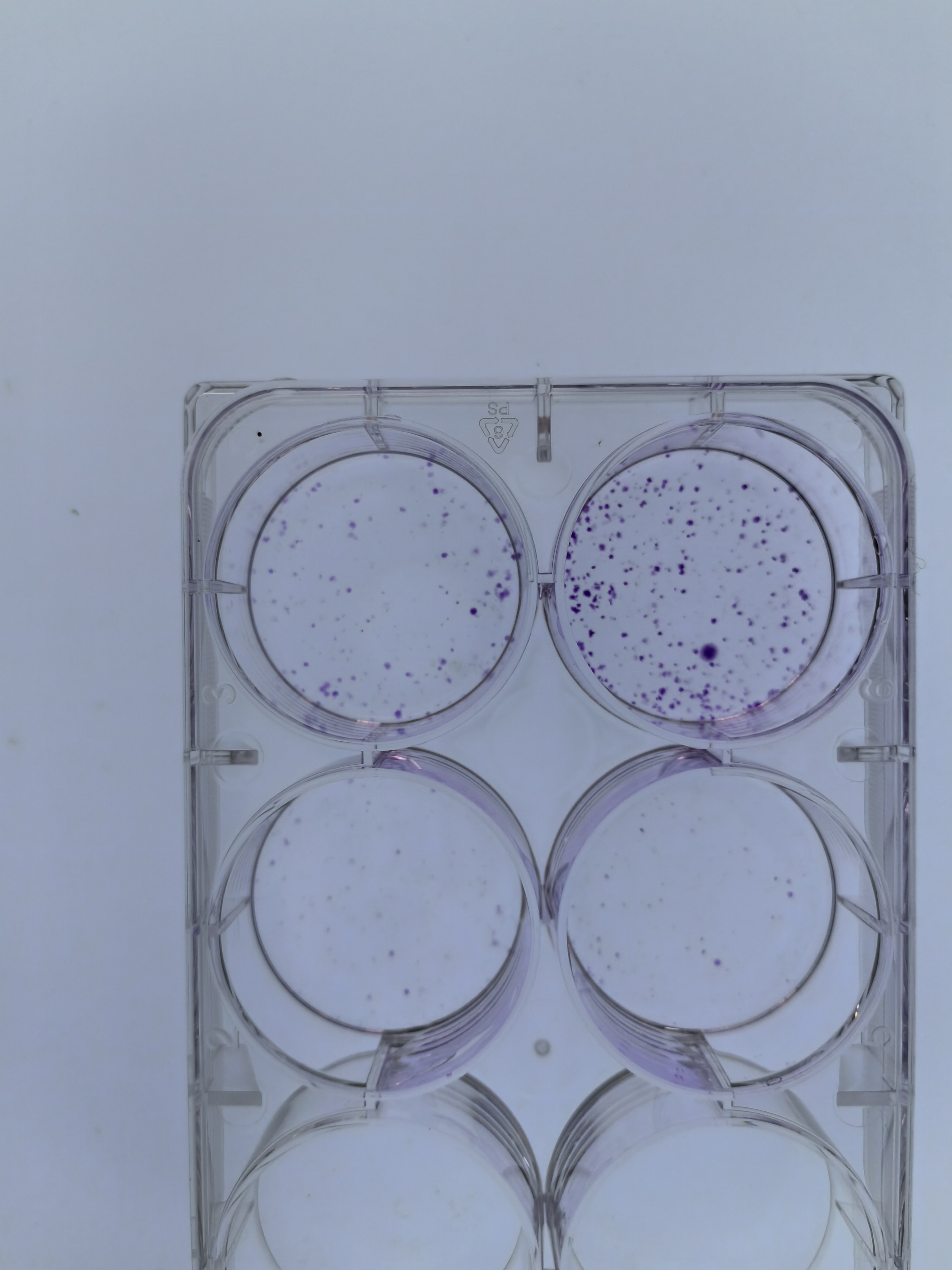

Supplement: Supplementary file 1 [file biomolecules-14-00677-s001.zip › Raw data/Huh 7/RPN1-Huh7-clone/_20240110101326.jpg]

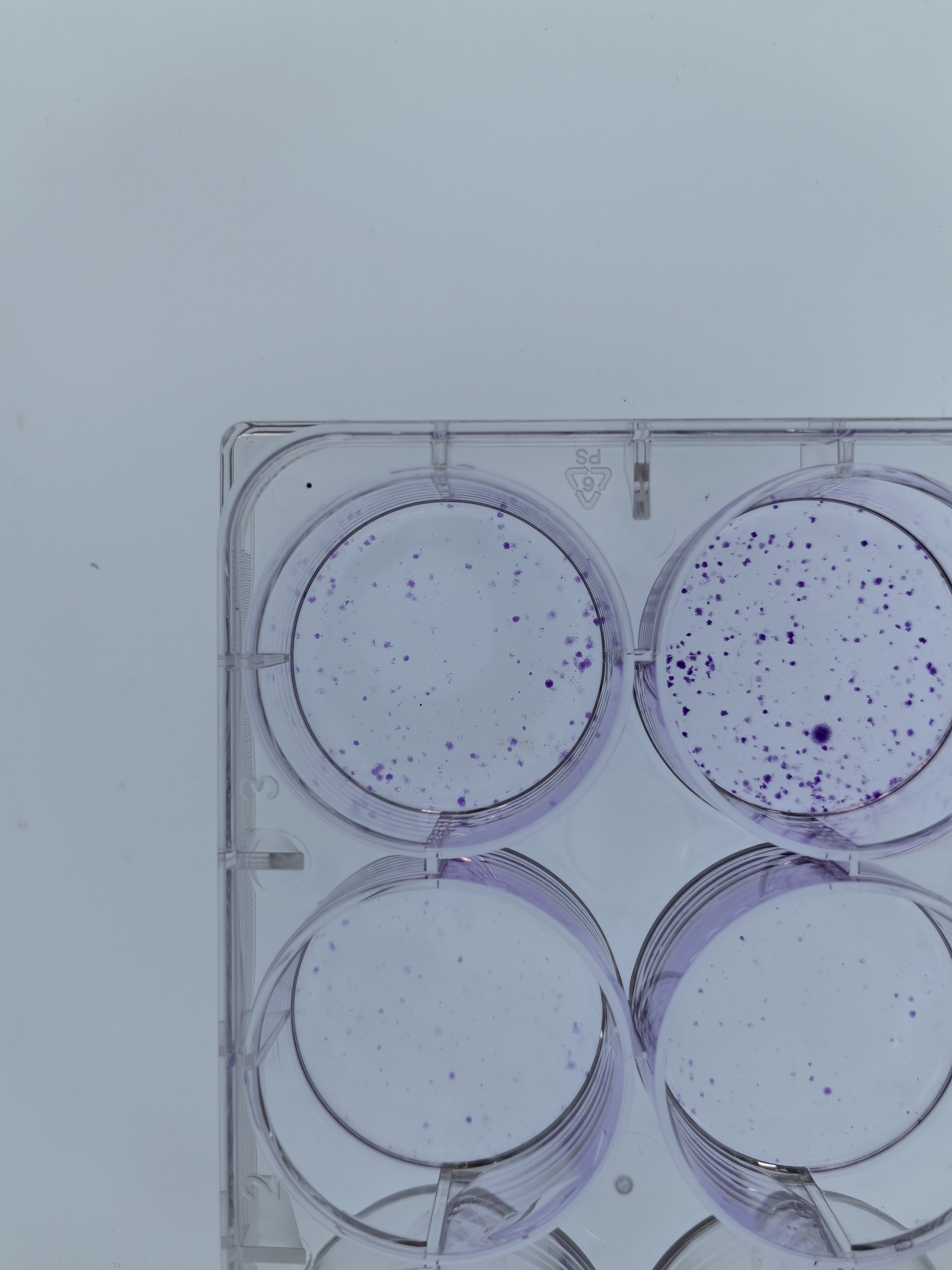

Supplement: Supplementary file 1 [file biomolecules-14-00677-s001.zip › Raw data/Huh 7/RPN1-Huh7-clone/_20240110101336.jpg]

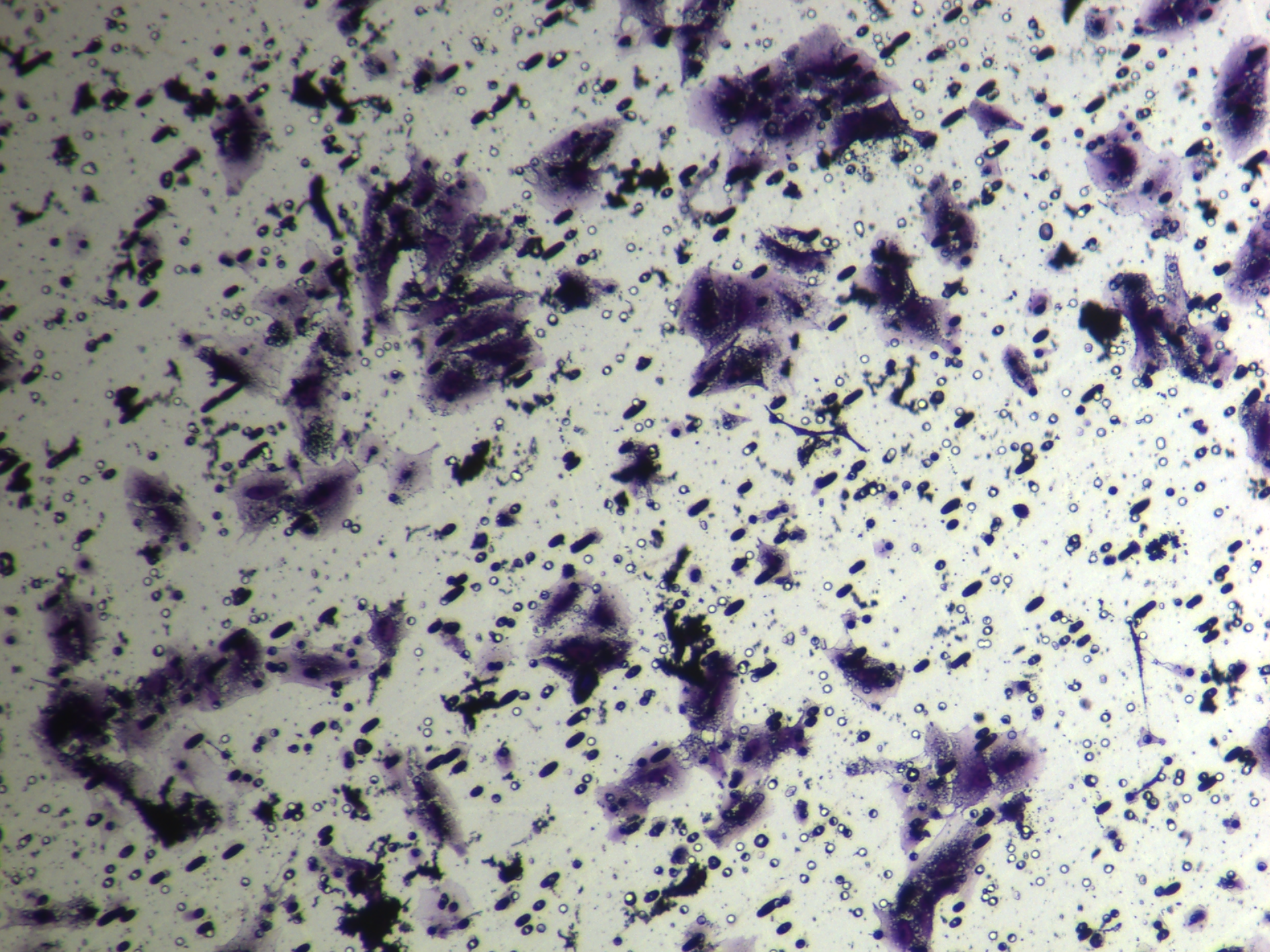

Supplement: Supplementary file 1 [file biomolecules-14-00677-s001.zip › Raw data/Huh 7/TRANSWELL-RPN1-CW/PLKO.1-HUH7.tif]

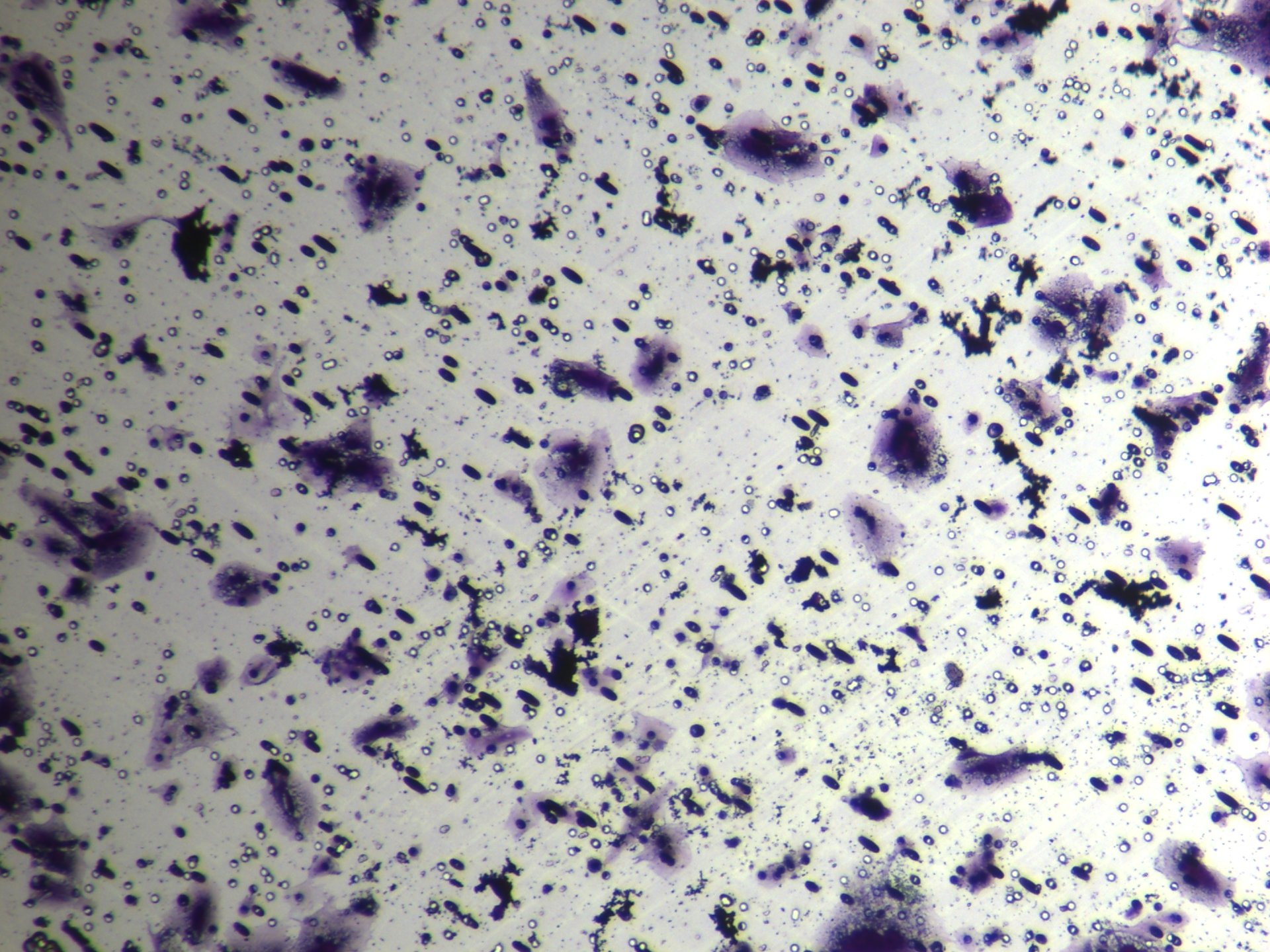

Supplement: Supplementary file 1 [file biomolecules-14-00677-s001.zip › Raw data/Huh 7/TRANSWELL-RPN1-CW/Series003_RAW_ch00.tif]

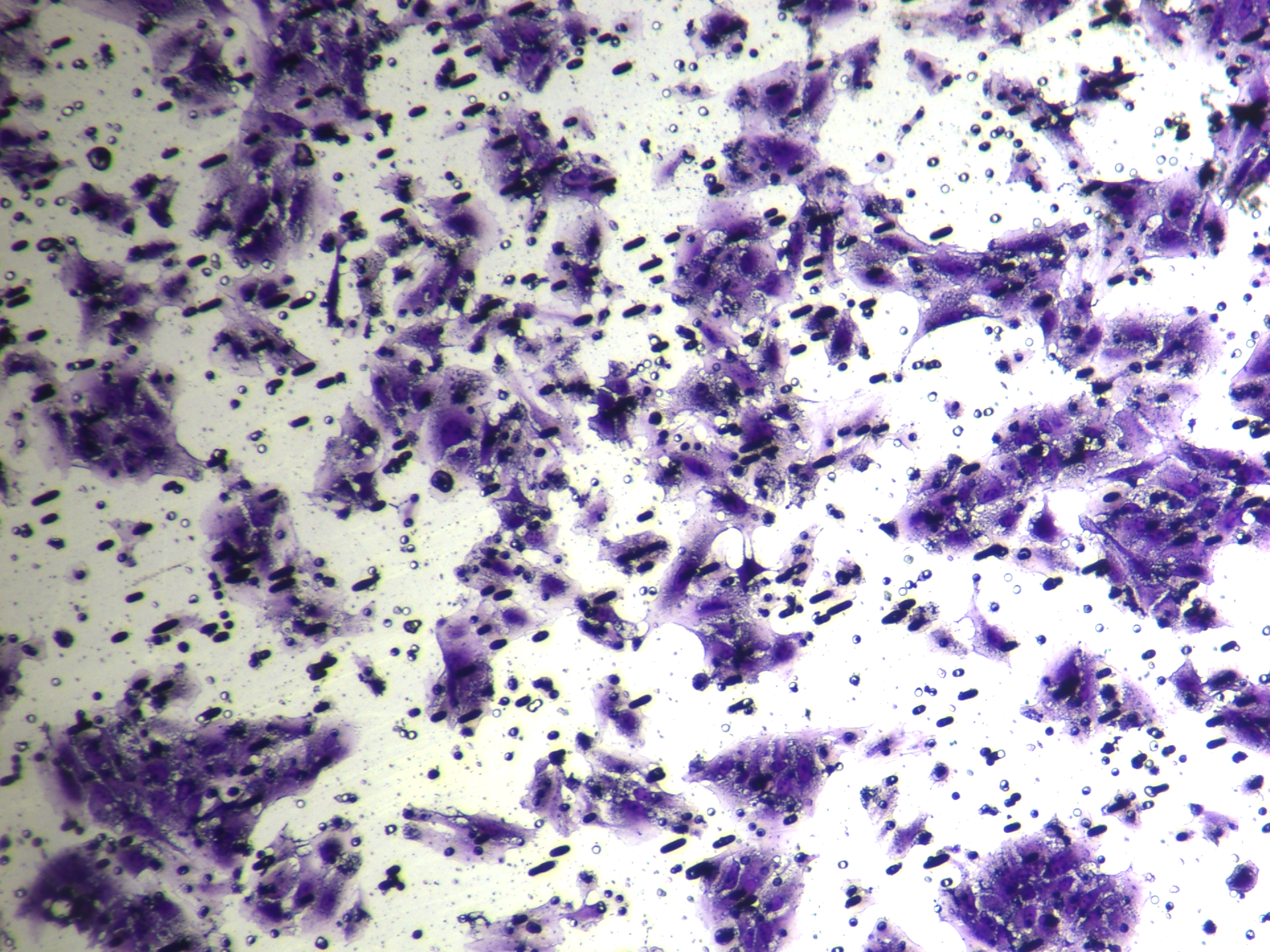

Supplement: Supplementary file 1 [file biomolecules-14-00677-s001.zip › Raw data/Huh 7/TRANSWELL-RPN1-CW/SH-RPN1-HUH7.tif]

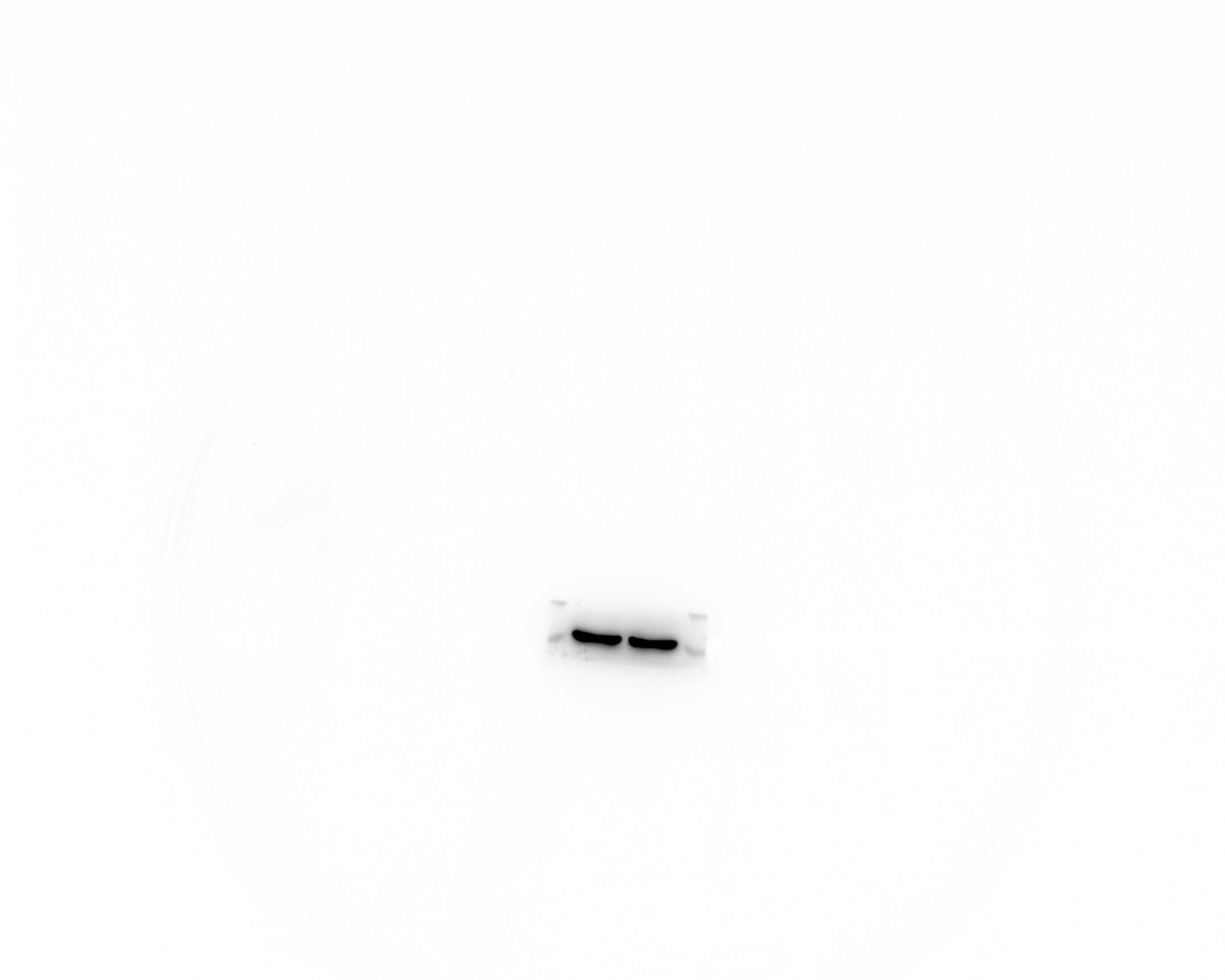

Supplement: Supplementary file 1 [file biomolecules-14-00677-s001.zip › Raw data/Huh 7/WB/GAPDH.jpg]

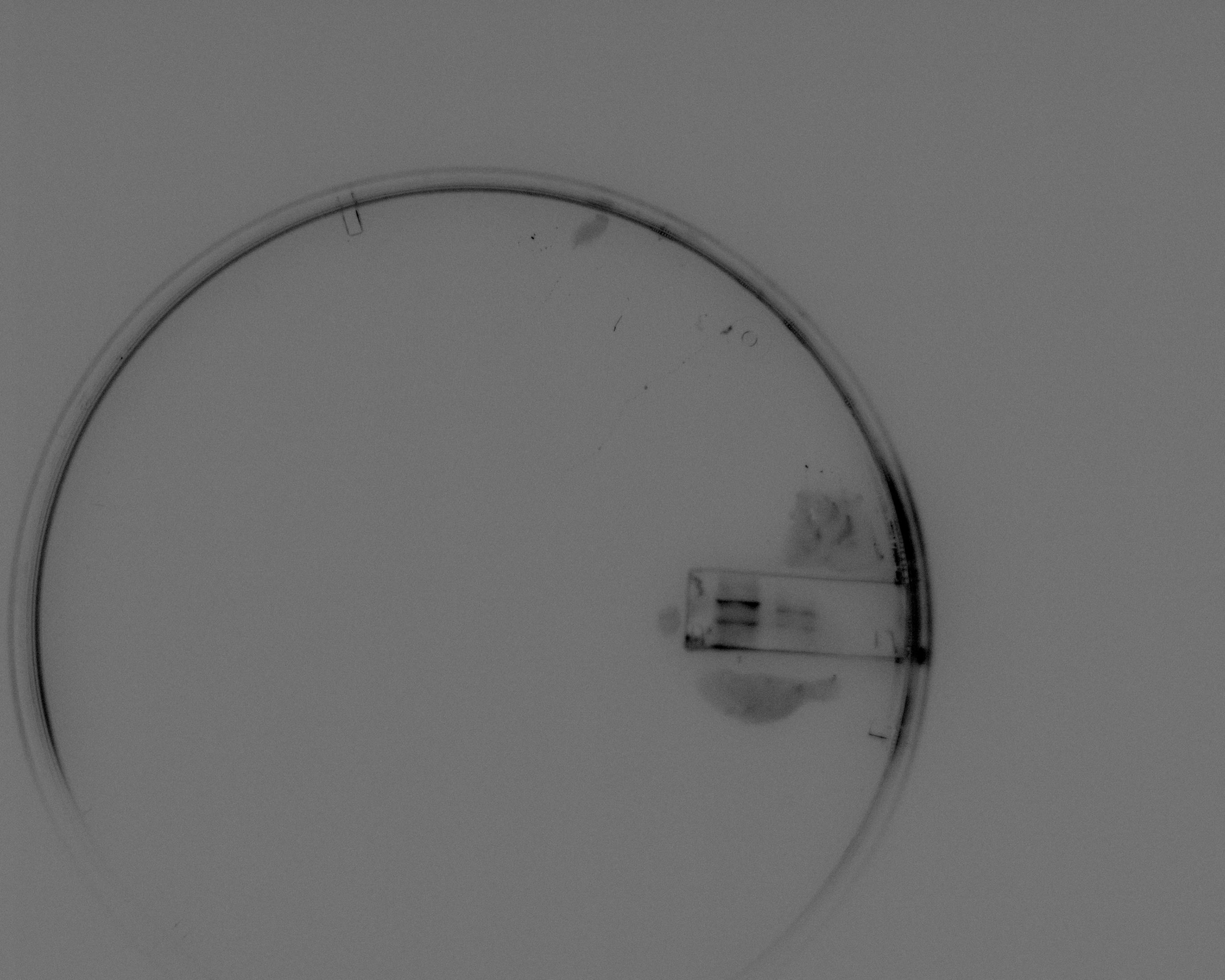

Supplement: Supplementary file 1 [file biomolecules-14-00677-s001.zip › Raw data/Huh 7/WB/rpn1-3.jpg]
