# Supplementary material for: Multi-Omics Insights into Disulfidptosis-Related Genes Reveal RPN1 as a Therapeutic Target for Liver Cancer
Source: Biomolecules. 2024 Jun 10;14(6):677. doi: 10.3390/biom14060677 (PMC11201601; doi:10.3390/biom14060677)
Supplement: Supplementary file 1 [file Figure S2.pdf › Figure S2.pdf]

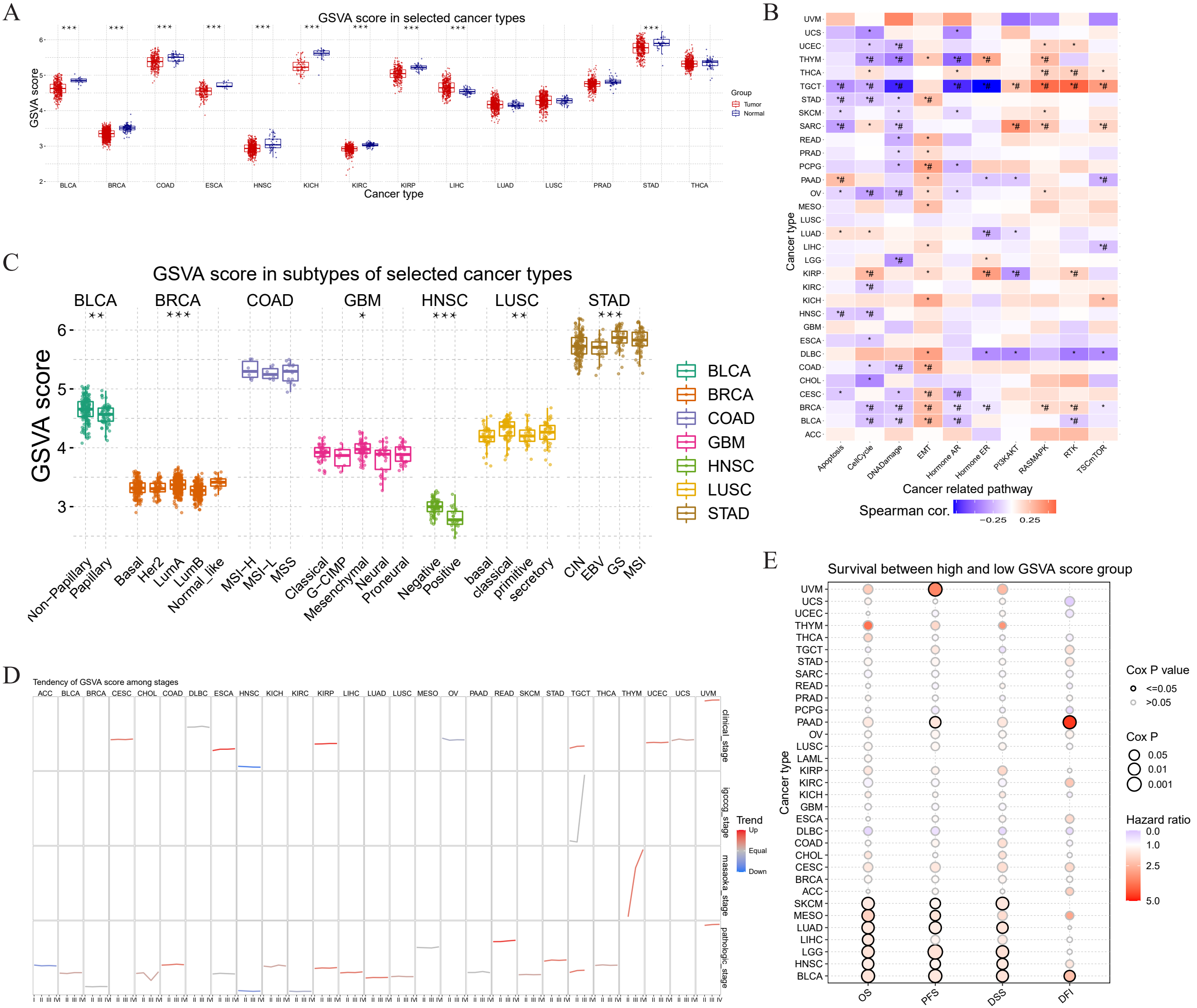
A
GSVA score in selected cancer types
GSVA score
Cancer type
BLCA BRCA COAD ESCA HNSC KICH KIRC KIRP LIHC LUAD LUSC PRAD STAD THCA
Group
Tumor
Normal
B
Cancer type
Cancer related pathway
Apoptosis CellCycle DNADamage EMT Hormone AR Hormone ER PI3KAKT RASMAPK RTK TSCmTOR
UVM UCS UCEC THYM THCA TGCT STAD SKCM SARC READ PRAD PCPG PAAD OV MESO LUSC LUAD LIHC LGG KIRP KIRC KICH HNSC GBM ESCA DLBC COAD CHOL CESC BRCA BLCA ACC
Spearman cor.
−0.25 0.25
C
GSVA score in subtypes of selected cancer types
BLCA BRCA COAD GBM HNSC LUSC STAD
GSVA score
Non−Papillary Papillary Basal Her2 LumA LumB Normal_like MSI−H MSI−L MSS Classical G−CIMP Mesenchymal Neural Proneural Negative Positive basal classical primitive secretory CIN EBV GS MSI
D
Tendency of GSVA score among stages
ACC BLCA BRCA CESC CHOL COAD DLBC ESCA HNSC KICH KIRC KIRP LIHC LUAD LUSC MESO OV PAAD READ SKCM STAD TGCT THCA THYM UCEC UCS UVM
clinical_stage
igcccg_stage
masaoka_stage
pathologic_stage
Trend
Up
Equal
Down
E
Survival between high and low GSVA score group
Cancer type
OS PFS DSS DFI
Cox P value
<=0.05
>0.05
Cox P
0.05
0.01
0.001
Hazard ratio
0.0
1.0
2.5
5.0
